# Cobalt Single‐Atom Nanozyme for Enhanced Intestinal Radioprotection and Tumor Radiosensitization via Bidirectional ROS Modulation

**DOI:** 10.1002/advs.76491

**Published:** 2026-07-07

**Authors:** Shengqi Yin, Junjie Li, Yishu Zou, Yang Liu, Yilin Zheng, Lu Yu, Wanying Zheng, Yajun Zou, Zehui Zhang, Tao Li, Peiqun Yin, Jianlin Zhang, Yuen Wu, Yi Ding

**Affiliations:** ^1^ Center of Radiation Oncology Department Nanfang Hospital Southern Medical University Guangzhou Guangdong P. R. China; ^2^ Guangdong Province Key Laboratory of Molecular Tumor Pathology Guangzhou Guangdong P. R. China; ^3^ School of Biomedical Engineering Research and Engineering Center of Biomedical Materials Anhui Medical University Hefei P. R. China; ^4^ The First Clinical Medical College of Jinan University The First Affiliated Hospital Jinan University Guangzhou Guangdong P. R. China; ^5^ Department of Emergency Surgery The First Affiliated Hospital of Anhui Medical University Hefei Anhui P. R. China; ^6^ Key Laboratory of Precision and Intelligent Chemistry University of Science and Technology of China Hefei P. R. China

**Keywords:** pH‐responsive, radioprotection, radiosensitization, ROS modulation, single‐atom nanozyme

## Abstract

Herein, we develop an orally administered cobalt single‐atom nanozyme (Co‐SAN) featuring pH‐responsive, bifunctional catalytic activity to enable simultaneous intestinal radioprotection and tumor radiosensitization. In the alkaline intestinal microenvironment, Co‐SAN effectively scavenges radiation‐induced reactive oxygen species (ROS)‐as validated by flow cytometry, thereby mitigating radiation‐induced intestinal injury (RIII). Mechanistically, RNA‐seq analysis reveals that beyond direct ROS elimination, Co‐SAN downregulates the ROS‐mediated PI3K/AKT signaling pathway, significantly suppressing the formation of detrimental neutrophil extracellular traps (NETs). Furthermore, this highly biocompatible nanozyme maintains gut microbiota homeostasis and preserves intestinal barrier integrity. In contrast, within the mildly acidic tumor microenvironment (TME), Co‐SAN undergoes a catalytic switch to promote ROS generation and ameliorate hypoxia, potently augmenting radiotherapeutic efficacy. Collectively, this study presents a bifunctional single‐atom nanozyme that resolves the spatial contradiction between normal tissue protection and targeted tumor sensitization, offering a promising paradigm to substantially widen the therapeutic window of radiotherapy.

## Graphical abstract

Orally administered, pH‐responsive Co‐SAN exerts dual functions: in the alkaline intestinal milieu, it scavenges reactive oxygen species (ROS) and suppresses the PI3K/AKT pathway and NETs formation, thus providing radioprotection; whereas in the acidic tumor microenvironment, it generates ROS and alleviates hypoxia, thereby enhancing radiosensitization.

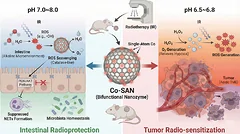

## 1 Introduction

Radiotherapy (RT) remains a cornerstone in the management of malignant tumors, inducing cell death through direct biomolecular destruction and indirect oxidative damage via the generation of reactive oxygen species (ROS) [1, 2]. However, the clinical efficacy of RT is often severely compromised by intrinsic tumor heterogeneity, particularly the hypoxic microenvironment, which confers significant radioresistance relative to normal tissues. Consequently, escalating the ionizing radiation (IR) dose to achieve tumor control inevitably inflicts irreversible damage on adjacent normal tissues, thereby dose‐limiting the curative potential of RT [3, 4]. The intestine is highly susceptible to IR; thus, radiation‐induced intestinal injury profoundly impairs the prognosis and quality of life in patients receiving abdominal or pelvic RT [5, 6]. Therefore, developing strategies that simultaneously protect normal intestinal tissues and augment the antitumoral efficacy of RT remains a critical but formidable challenge. Although amifostine, the only clinically approved radioprotectant, mitigates intestinal damage by enhancing crypt cell survival, its clinical utility is hindered by the requirement for intravenous administration and substantial systemic toxicity [7, 8, 9, 10, 11]. Alternative small‐molecule radioprotectants, such as vitamin E and astaxanthin, offer some protection by scavenging ROS [12]. Nevertheless, their poor aqueous solubility, short circulation half‐lives, and rapid metabolism severely impede the development of effective oral formulations [13, 14, 15]. More importantly, non‐selective ROS scavenging intended for intestinal protection inadvertently neutralizes the oxidative stress required for tumor eradication—a critical trade‐off that is frequently overlooked [11, 13, 16]. Consequently, there is an urgent need to engineer novel, orally administrable radioprotective agents with high stability and selective protective mechanisms.

Single‐atom nanozymes (SANs), characterized by well‐defined atomically dispersed active sites that mimic natural enzymes, synergistically integrate the advantages of both biocatalysts and conventional nanozymes, thereby exhibiting exceptional catalytic activity, robust stability, and cost‐effectiveness [17, 18, 19, 20]. The field of SANs has advanced rapidly in recent years; specifically, multienzyme‐like activities—including peroxidase (POD), superoxide dismutase (SOD), and catalase (CAT) mimics—have been systematically realized through meticulous atomic‐level engineering [21, 22, 23]. Consequently, SANs hold immense translational promise as natural enzyme surrogates and have achieved significant milestones in biomedical applications [24]. However, fabricating bifunctional SANs with different enzymatic activities in response to a dynamic biological microenvironment to employ in diverse biomedical applications presents a formidable challenge. Furthermore, the underlying biological mechanisms governing their in vivo efficacy remain largely elusive [25].

Herein, we report the development of an orally administered, pH‐responsive cobalt single‐atom nanozyme (Co‐SAN) exhibiting distinct multienzyme‐mimicking activities, which can fulfill dual outcomes encompassing the enhancement of radiotherapeutic efficacy and intestinal protection against radiation‐induce injury for abdominal/pelvic tumor management. Initially, Co‐SAN demonstrates remarkable stability within the acidic gastric environment, ensuring its successful transit and prolonged retention in the intestinal tract. Notably, within the mildly alkaline intestinal microenvironment, Co‐SAN exhibits the antioxidant properties of CAT, SOD, and GPx to eradicate excess ROS, strongly and rapidly attenuating adiotherapy‐associated toxicity and preserves intestinal integrity (Scheme 1). Conversely, in the mildly acid tumor microenvironment, Co‐SAN shifts to exhibit OXD, NOX, GSHOx and CAT activities, which synergistically augment radiotherapeutic efficacy and accelerate tumor cell death.

**SCHEME 1:**
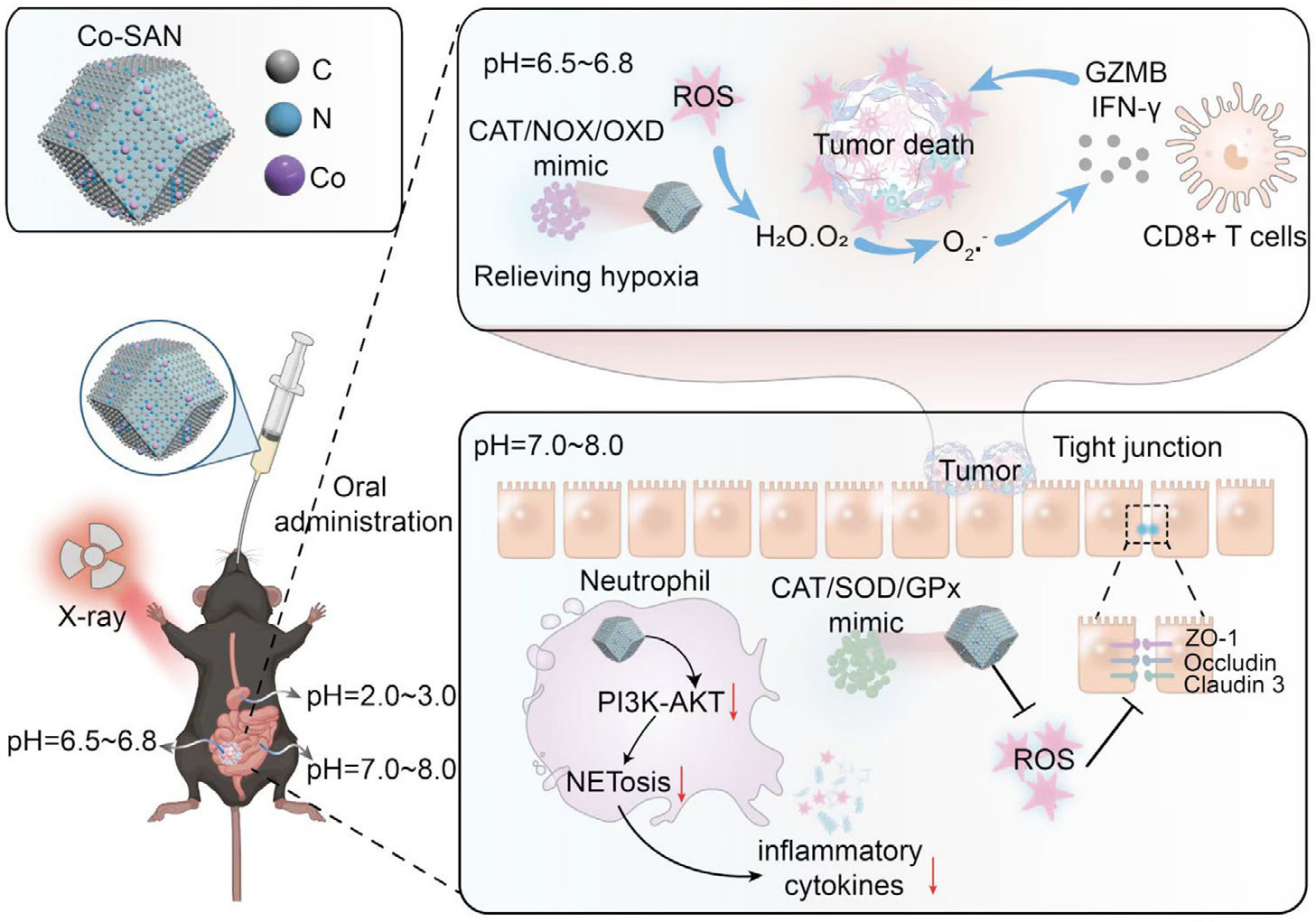
The schematic illustration of an oral‐administrated Co‐SAN for intestinal protection and the enhancement of cancer radiotherapy.

Particularly, a novel mechanism is also unraveled for its prevention of radioactive intestinal injury. As we know, neutrophils are one of the earliest cells recruited to the tissues of acute injury after radiation, thus leading to a series of inflammatory reactions [26, 27, 28, 29], and NETs exacerbate normal cell apoptosis and tissue damage [30, 31, 32]. Transcriptomic and flow cytometry analyses reveal that Co‐SAN shields the intestine not only by directly neutralizing ROS but also by suppressing excessive NET formation via the down‐regulation of the ROS‐mediated PI3K/AKT signaling pathway. Collectively, these findings present a promising paradigm for the simultaneous optimization of tumor radiotherapy and the mitigation of acute gastrointestinal toxicity.

## 2 Results and Discussion

### 2.1 Synthesis and Characterization of Co‐SAN

Herein, we successfully synthesized a Co single‐atom nanozyme (Co‐SAN) by a general pyrolysis strategy using ZnCo‐MOF as the precursor, exhibiting multi‐enzyme activities via pH‐response. As shown in representative transmission electron microscopy (TEM) and scanning electron microscopy (SEM) images, the morphology of Co‐SAN maintained the initial dodecahedral shape of ZnCo‐MOF (Figure S1a,b), and Co nanoparticles (NPs) were absent in the obtained sample compared with the counterpart of Co‐NPs derived from ZIF‐67 (Figure 1a and Figures S1c,d and S2). X‐ray diffraction (XRD) was also conducted to characterize the obtained samples. The XRD pattern of Co‐SAN showed no discernible diffraction peaks originating from crystalline Co NPs, confirming that Co species are predominantly dispersed as isolated single atoms without crystalline cobalt. Meanwhile, only a broad shoulder peak ranging from 20° to 30° (2θ) was detected, which is ascribe to graphitic carbon (Figure S4b) [33]. The high‐angle annular dark‐field scanning transmission electron microscopy (HAADF‐STEM) images further confirmed the uniform dispersion of single Co atoms rather than Co nanoparticles through the carbon matrix, as depicted in Figure 1c–e. The corresponding elemental mapping distinctly demonstrated the homogeneous distribution of Co, C, N elements throughout the whole domain, further indicating the successful generation of Co‐SAN (Figure 1f). The mass loading of Co for Co‐SAN was identified to be 2.57 wt.% by inductively coupled plasma atomic emission spectrometry (ICP‐AES), which was consistent with the result of EDS analysis (2.48 wt.%). The sufficient mass loading is favorable for the available active sites to achieve superior catalytic activities (Table S1). Furthermore, the Brunauer‐Emmett‐Teller (BET) measurements and the corresponding pore size distribution are displayed in Figure S5 and Table S2, revealing that Co‐SAN inherited the high specific surface area and porous structure of ZnCo‐MOF, compared with Co‐NPs. The high specific surface area and porous construction are beneficial for the exposure of active Co sites to achieve superior catalytic performance. X‐ray photoelectron spectroscopy (XPS) was performed to elucidate the chemical states of Co, C, and N (Figure S6). The C1s spectrum of Co‐SANs could be assigned to four peaks, including C═O, C─C, C─O. Apart from pyridinic N, pyrrolic N, Graphitic N, and oxidized N configurations were presented in high‐resolution N 1s XPS, Co─N bond could also be sighted, indicating sufficient sites to anchor Co single atoms. Importantly, the peak of binding energy of Co 2p could be deconvoluted into nonzero valence Co, demonstrating the presence of Co oxidation state (Figure S6c). Furthermore, the X‐ray absorption fine structure (XAFS) measurements were carried out to elaborate on the binding states of Co species. As revealed by Co K‐edge X‐ray absorption near‐edge structure (XANES) spectra in Figure 1g, the absorption edge position of the Co‐SAN closely resembles that of CoO, indicating an approximate +2 oxidation state of the Co species, consistent with the Co 2p XPS analysis. The Fourier‐transformed extended X‐ray absorption fine structure (FT‐EXAFS) provides more detailed information about the coordination conformation at the atomic level. For Co‐SAN, a prominent peak around 1.4 Å was observed, corresponding to the first‐shell Co─C/N/O coordination (Figure 1h). Notably, no Co‐Co scattering peak around 2.4 Å was observed, confirming the absence of Co nanoparticles in Co‐SAN, coinciding with the XRD result. Quantitative EXAFS fitting determined a Co‐C/N/O coordination number of N = 4.1 at an interatomic distance of R = 1.91 Å (Table S3, Figure 1j,k). Given the inherent limitation of EXAFS in distinguishing backscattering atoms with similar atomic numbers, complementary XANES and theoretical studies were employed to resolve the precise local structure. These results demonstrate that the principal coordination structures of Co single atoms in Co‐SAN are pyridinic Co‐N_4_ (see Discussion in Figures S31 and S32). To further identify the coordination surrounding of Co, wavelet transform (WT) of Co K‐edge was utilized as a complementary analysis to the EXAFS analysis. As displayed in Figure S11, the single signal from WT maxima around 5 Å could be assigned to the Co─N bond. Compared with Co foil, no maximum intensity peak at 7 Å associated with the Co─Co interaction was detected in Co‐SAN, which was coinciding with EXAFS analysis. Thus, the above results suggested that the Co‐SAN featuring with atomically dispersed Co‐N_4_ configurations was successfully achieved.

**FIGURE 1:**
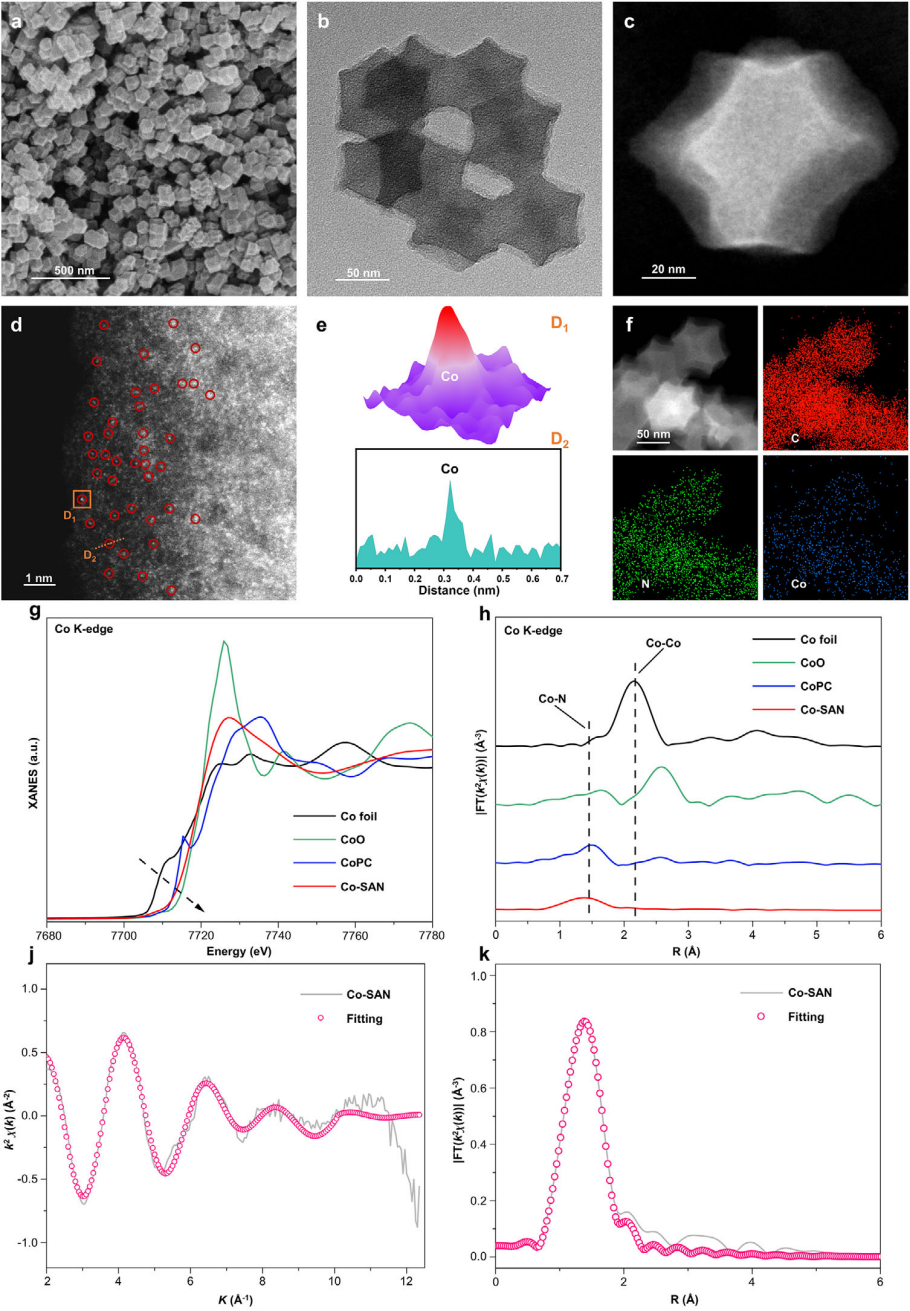
Characterization of the obtained Co‐SAN. (a) SEM image. (b) TEM image. (c) A representative image of HAADF‐STEM image. (d) Aberration corrected HAADF‐STEM with sub‐angstrom resolution image. (e) the corresponding intensity of surface profile (D_1_) and line profile (D_2_) taken from (d). (f) HAADF‐STEM image and the corresponding EDS elemental mappings of Co, N, C. (g) XANES spectra at Co k‐edge of Co foil, CoO, CoPc, and Co‐SAN. (h) the corresponding Fourier transform of EXAFS spectra of Co k‐edge. (j,k) The corresponding EXAFS fitting curves of Co‐SAN at k space (j) and R space (k).

### 2.2 Enzyme‐Mimicking Activities of Co‐SAN

Inspired by the structural similarities with natural enzymes, we investigated the multiple enzyme‐mimicking activity of Co‐SAN. The pH‐responsive catalytic profiles of Co‐SAN were illustrated in Figure 2a. Initially, the reactive oxygen species (ROS)‐scavenging activity of Co‐SAN was evaluated by ABTS and DPPH free radical scavenging assay. Compared to Co‐NPs and N/C, the best ABTS^·+^ and DPPH• scavenging activities were achieved by Co‐SAN, as evidenced by the UV–vis absorbance spectra shown in Figure S12a,b. Notably, the ABTS radical scavenging ratio of Co‐SAN was modest at pH = 7.4, and the activity of Co‐SAN increased gradually by elevating the pH value, indicating a significant pH‐dependence of the antioxidative capacity of Co‐SAN (Figure S12c). To investigate the chemical stability of Co‐SAN, it was dispersed in a buffer at pH = 1.2 and aged for 3 h. Remarkably, the ability of scavenging ABTS^·+^ remained superior and was positively correlated with the concentrations of Co‐SAN, implying the robust stability of Co‐SAN in harsh acid conditions (Figure S12d). Encouraged by this phenomenon, the enzyme‐like activities of Co‐SAN under various conditions were further assessed. Co‐SAN demonstrated pronounced catalase (CAT)‐mimicking activity, catalyzing O_2_ generation from H_2_O_2_ far more efficiently than Co‐NPs and N/C (Figure 2b). Intriguingly, Co‐SAN exhibited high H_2_O_2_ decomposition efficiency under wide ranges of pH, implying its outstanding CAT‐like property in a broad practical employment (Figure 2c). Additionally, Co‐SAN effectively eliminated H_2_O_2_ in the presence of glutathione (GSH) in a pH‐dependence manner, suggesting its favorable glutathione peroxidase (GPx)‐mimicking activity (Figure S13 and Figure 2d). Notably, to determine the O_2_•^−^ elimination efficiency (SOD‐mimicking activity) of Co‐SAN, a total SOD activity assay was performed across various concentrations of Co‐SAN (Figure 2e). As confirmed, Co‐SAN exhibits a robust, concentration‐dependent scavenging capacity, whereby its superoxide dismutase (SOD)‐like activity scales proportionally with catalyst dosage ranging from 5 to 80 µg/mL. To elucidate the underlying mechanisms governing its enzyme‐mimicking properties and pH‐responsive behavior, density functional theory (DFT) calculations were conducted to map the reaction pathways and free energy profiles in both acidic (pH = 0) and alkaline (pH = 14) environments (Figure S19e,f). In the context of the SOD catalytic pathway, although an acidic milieu thermodynamically stabilizes the reaction intermediates and lowers the overall free energy landscape, it concurrently induces an over‐binding of the ^*^H_2_O‐O_2_ species. This excessive thermodynamic stabilization deepens the potential energy well of the intermediate, thereby severely impeding the rate‐determining step (the conversion of ^*^H_2_O‐O_2_ to ^*^H_2_O). Consequently, the activation energy barrier is elevated from 0.79 eV under alkaline conditions to 0.80 eV under acidic ones, culminating in a dampened intrinsic catalytic efficiency in low‐pH media. Conversely, within the neutral‐to‐alkaline environment characteristic of the intestinal tract, this over‐binding phenomenon is mitigated, and the activation barrier is correspondingly reduced to facilitate highly efficient SOD‐like activity. Ultimately, exploiting this pH‐dependent mechanism, the Co‐SAN can eliminate H_2_O_2_ and O_2_•^−^ effectively by mimicking the activities of SOD, CAT, and GPx, demonstrating exceptional reactive ROS scavenging performance under alkaline conditions. The oxidation of 1,2‐diaminobenzene (OPD) was employed as a typical catalytic reaction to investigate the oxidase (OXD)‐like activities of Co‐SAN. As illustrated in Figure S14, the yellow‐colored reaction solution with the characteristic absorption peak was located at 417 nm, indicating the optimal oxidase (OXD)‐like activity, compared with Co‐NPs and N/C. Moreover, the OXD‐mimicking activity of Co‐SAN was improved with the decrease of the pH value, while remaining negligible at neutral pH (Figure 2f). Subsequently, the POD‐like catalytic activity of Co‐SAN was further explored. Analogous to the OXD‐like activity, Co‐SAN demonstrated an excellent POD‐like catalytic activity only in an acidic environment (Figure S17). These experiment observations are strongly corroborated by our density functional theory (DFT) simulations. As mapped out in the free energy diagrams for both the oxidase (OXD) and peroxidase (POD) pathways, the reaction thermodynamics are markedly more favorable at pH = 0 relative to pH = 14. The theoretical calculations unambiguously validate the pronounced preference of the Co‐SAN to manifest its OXD‐ and POD‐like catalytic activities within acidic microenvironments (Figure S19a–d). Given that NAD(P)H oxidase (NOX)‐mimicking activity is closely associated with the intra‐tumor metabolism [34]. Therefore, the oxidase‐mimicking activity toward NAD(P)H of Co‐SAN was explored by UV–vis absorption spectroscopy. Notably, a prominent decrease of the typical absorption peak of NADH at 340 nm and an increased absorption peak of NAD^+^ at 260 nm were evident with prolonged time (Figure 2g), demonstrating the high NOX‐mimicking activity of Co‐SAN through the gradual oxidation of NADH into NAD^+^. Meanwhile, in the NADH oxidation reaction involving Co‐SAN, H_2_O_2_ production was clearly detected with the increase of NADH concentration, which was conducive to the generation of •OH during POD‐mimicking activity (Figure S16a). Additionally, owing to the similarity to NADH, Co‐SAN showed significantly oxidase‐like activity toward NADPH as well (Figure 2h). To determine the influence of pH values, which similarly displayed a distinct pH‐dependency (Figure S16b). The consumption of NAD(P)H exhibited a significant decline when the pH values increased, suggesting the optimal condition for an enhanced NADH oxidase‐like activity of Co‐SAN was acidic surroundings. To further elucidate the ROS species, electron spin resonance (ESR) was employed by using 5,5‐dimethy1‐1‐pyrroline N‐oxide (DMPO) as a trapping agent. The generation of O_2_•^−^ in the reaction system was verified by the characteristic signal of the DMPO‐OOH adduct (Figure 2i). As expected, due to this NOX‐mimicking catalytic property of Co‐SAN, the signal intensities of O_2_•^−^ showed a slightly increase when the Co‐SAN underwent NADPH treatment, in comparison with Co‐SAN alone, concurrently suggesting the superior NOX and OXD‐mimicking activities. As illustrated in Figure 2j, a distinct peak with an intensity ratio of 1:2:2:1 was significantly detected in the presence of Co‐SAN mixed with H_2_O_2_, ascribed to the production of •OH, indicating that Co‐SAN can be a potential nanozyme to substitute native POD. Besides, Co‐SAN induced prominent the effective consumption of glutathione (GSH) in a concentration‐dependent manner, compared with the counterparts of N/C and Co‐NPs, thereby confirming its excellent glutathione oxidase (GSHOx) activity (Figure 2k, Figure S15). In terms of the acidic nature of the tumor microenvironment, Co‐SAN can exert these oxidase properties to improve the generation of ROS and relieve hypoxia in tumor, underscoring the significant potential of Co‐SAN to enhance the efficacy of radiotherapy. Furthermore, to quantitatively evaluate the enzymatic activity of Co‐SAN, Michaelis‐Menten kinetic analysis was conducted to determine the key enzymatic parameters, including Michaelis‐Menten constant (*K_m_*) and maximum reaction velocity (*V_max_*) for CAT, OXD, and POD activities (Figure S18). All reactions followed typical Michaelis‐Menten kinetics. Notably, Co‐SAN exhibited lower *K_m_* and higher *V_max_* values compared to most reported single‐atom nanozymes, demonstrating its superior substrate affinity and enhanced catalytic efficiency (Tables S5–S7).

**FIGURE 2:**
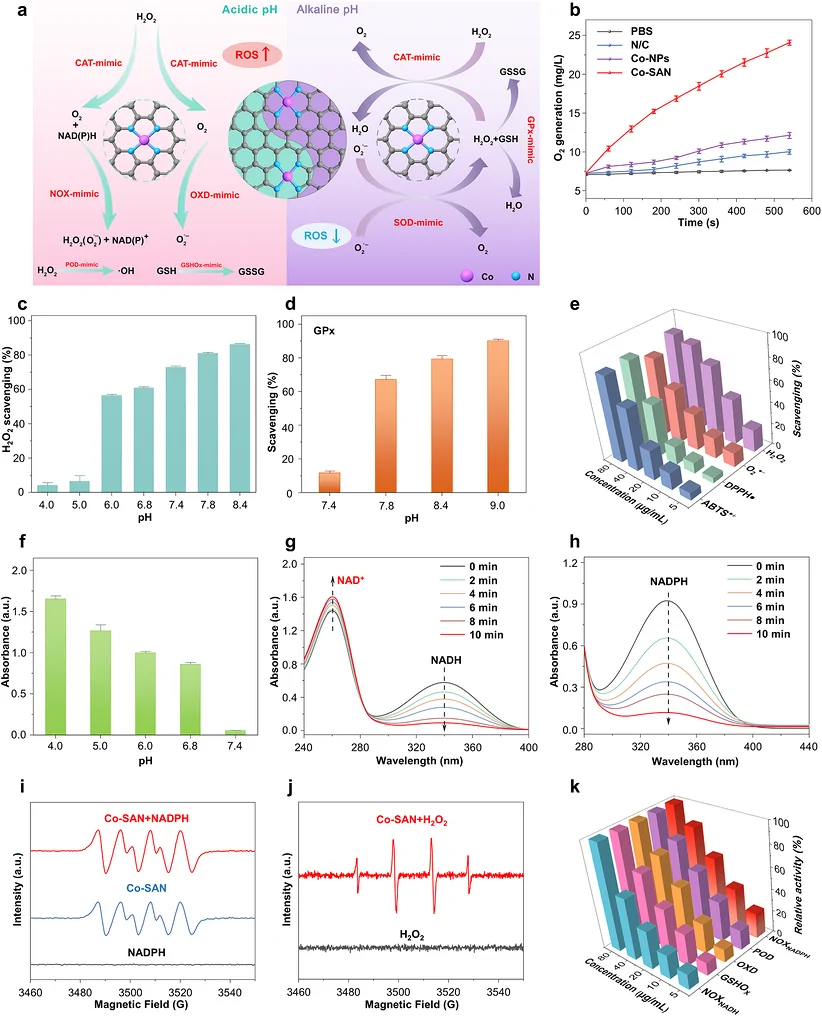
Multi‐enzyme‐mimicking properties of the Co‐SAN. (a) Schematic illustration of pH‐responsive multi‐enzyme‐mimicking activity of Co‐SAN. (b) The time‐dependent O_2_ generation efficacy when 10 mM H_2_O_2_ mixed with PBS, N/C, Co‐NPs, and Co‐SAN. (n = 3 independent experiments and data are presented as mean ± SD.) (c) pH‐dependent study of H_2_O_2_ scavenging efficacy of Co‐SAN. (n = 3 independent experiments and data are presented as mean ± SD.) (d) GSH consumption of Co‐SAN under different pH. (n = 3 independent experiments and data are presented as mean ± SD.) (e) ABTS^·+^, DPPH^·^, O_2_^·−^, H_2_O_2_ scavenging efficacy of Co‐SAN with various concentrations. (f) OXD‐mimicking activity of Co‐SAN under different pH conditions. (n = 3 independent experiments and data are presented as mean ± SD.) (g) UV–vis spectra of NADH oxidation catalyzed by Co‐SAN. (h) UV–vis spectra of NADPH oxidation catalyzed by Co‐SAN. (i) ESR spectra of O2^·−^ detected by a trapping agent of DMPO. (j) ·OH detection by ESR spectra. (k) The investigation of NOX, OXD, POD, GSHOx‐mimicking activities of Co‐SAN with various concentrations.

In summary, the Co‐SAN exhibits CAT, POD, OXD, and NOX‐mimicking activities in acid environment while CAT, SOD, and GPx‐mimicking activities in an alkaline environment. This unique enzymatic plasticity provides a robust theoretical rationale for its dual application in tumor management. Specifically, within the mildly alkaline intestinal tract, Co‐SAN acts as a potent radioprotectant by aggressively scavenging radiation‐induced ROS to prevent normal tissue oxidative damage. Conversely, in the acidic tumor microenvironment, it functions as an effective radiosensitizer by simultaneously alleviating hypoxia and amplifying intratumoral ROS generation. Ultimately, this paradigm holds great potential to augment the curative efficacy of radiotherapy and effectively alleviate off‐target radiation toxicity. To validate this dual‐therapeutic hypothesis, comprehensive in vitro and in vivo evaluations were subsequently conducted.

### 2.3 In Vitro Radiation Protection

Since Co‐SAN possessed exceptional multi‐enzyme activities and could play a critical role in scavenging ROS under alkaline environments efficiently, we first investigated the cytotoxicity of Co‐SAN and subsequently studied its application and effect in radiobiology. In IEC‐6 cells, we explored the effect of Co‐SAN, Co‐NPs, and N/C with various concentrations on cell viability by CCK8 experiments, and found that Co‐SAN was almost non‐cytotoxic and negligibly affected cell viability (Figure 3a). Radiation induces cellular damage through both direct macromolecular destruction and indirect oxidative stress. To assess the mitigation of radiation‐induced DNA double‐strand breaks, we monitored γ‐H2AX, a standard biomarker for DNA damage. Compared with Co‐NPs and N/C, the Co‐SAN showcased the least fluorescence counts, highlighting the best protective ability on radiation damage (Figure 3b). We conducted clonogenic survival assays to evaluate the radioprotective efficacy of Co‐SAN on normal (IEC‐6) and tumor (CT‐26) cells across varying radiation doses (Figure 3c,d). Co‐SAN treatment dramatically remained the good cell survival rates on IEC‐6 under radiation with different doses, indicating the outstanding radioprotective effect on normal cells (Figure 3e). Conversely, it conferred negligible protection to CT‐26 tumor cells under identical conditions, confirming that Co‐SAN selectively safeguards normal tissues without compromising the efficacy of tumor radiotherapy (Figure 3f). Additionally, ROS is also a critical factor for normal tissue damage, and endogenous antioxidant enzymes are overwhelmed or inactivated post‐irradiation [11, 35]. Subsequently, we verified the ROS scavenging effect of Co‐SAN in vitro by fluorescence staining and flow cytometry, the fluorescence intensity of DCF in the Co‐SAN group was the lowest compared with the other groups, and was comparable to the normal level (Figure 3g,h and Figure S20a). All these results demonstrated the excellent ROS scavenging and radiation protection ability of Co‐SAN in vitro.

**FIGURE 3:**
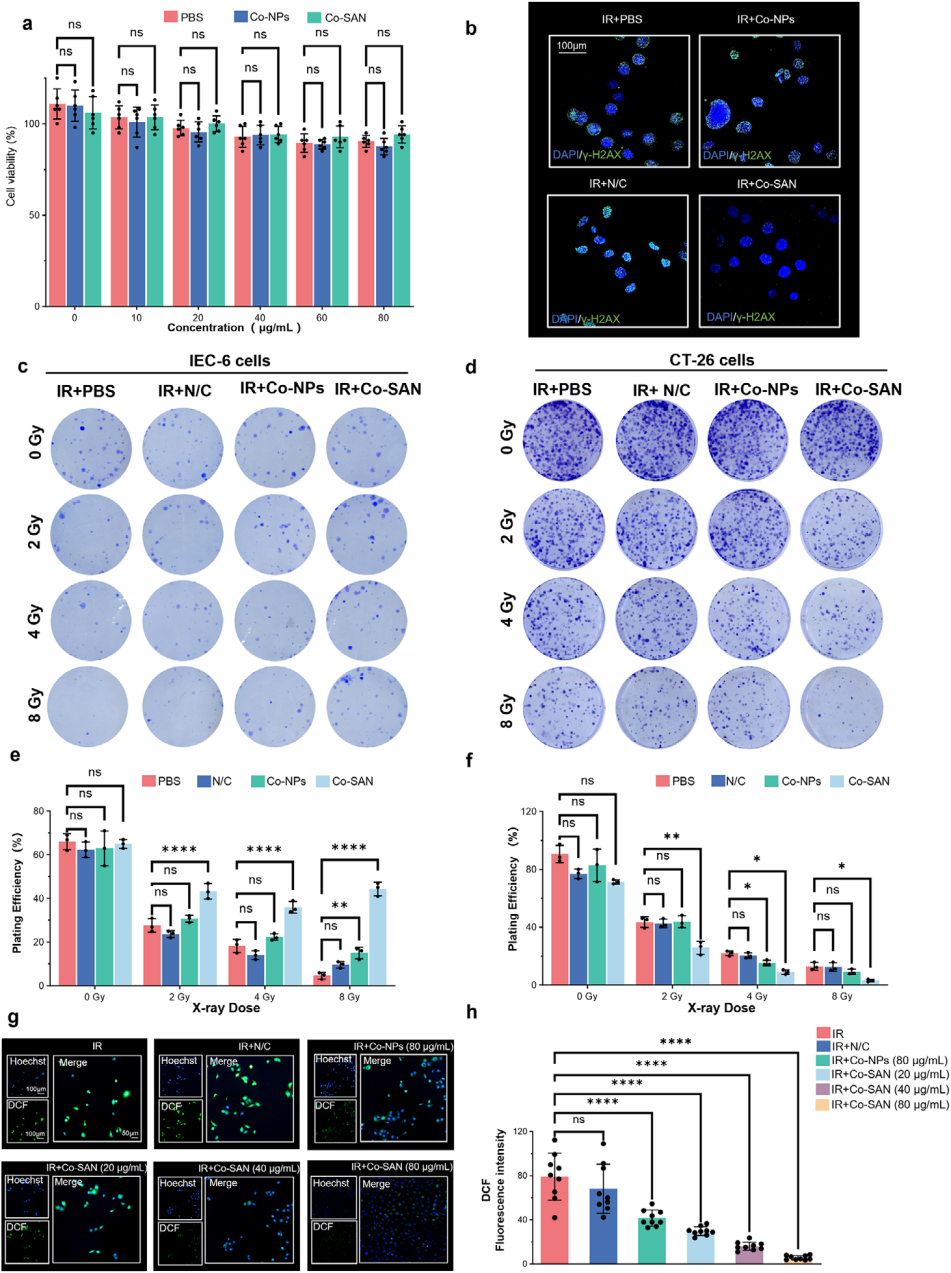
In vitro toxicity and radioprotective effects of Co‐SAN. (a) Viabilities of the IEC‐6 cells (small intestinal epithelium cells) after incubating with various concentrations of PBS, Co‐NPs, and Co‐SAN for 24 h. The viabilities were determined by an CCK8 assay kit (*n* = 6 biologically independent cells). The data show means + SD. *P* was calculated using two‐tailed *t*‐test. (b) γ‐H2AX immunofluorescence images (green, γ‐H2AX; blue, cell nucleus) of the NCM460 cells irradiated by 8 Gy X‐ray (IR) after 4 h of incubation with the renewed medium in different groups. Scale bar = 100 µm. The experiment was repeated five times independently with similar results. (c–f) Crystal violet staining (c, d) and quantification (e, f) of the surviving colonies of IEC‐6 cells and CT26 cells (colorectal cancer cells) irradiated by 0, 2, 4, and 8 Gy X‐ray in different treatment groups (PBS, N/C, Co‐NPs, and Co‐SAN) (*n* = 3 biologically independent cells). The data show means ± SD. “Data were analyzed by one‐way ANOVA followed by Dunnett's multiple comparisons test (vs. IR+Co‐SAN group), ^***^ *p* <0.001”. (g,h) Confocal fluorescent images of ROS levels (green, DCF; blue, Hoechst) in the ICE‐6 cells irradiated by 8 Gy X‐ray (IR) after 2 h of incubation with the renewed medium in different groups. Scale bar = 200 µm. Experiment was repeated nine times independently with similar results. The data show means ± SD. “Data were analyzed by one‐way ANOVA followed by Dunnett's multiple comparisons test (vs. IR+Co‐SAN (80 ug/mL) group). ^***^ *p* <0.001”.

### 2.4 Oral Administration of Co‐SAN Protects Radiation‐Induced Injury In Vivo

Prolonged intestinal retention is a prerequisite for Co‐SAN to effectively exert its radioprotective functions. Following oral administration, the nanozyme must initially endure the harsh gastrointestinal (GI) environment, particularly the highly acidic gastric fluid. To evaluate its GI stability, Co‐SAN was sequentially incubated in simulated gastric fluid (SGF) for 1 h and simulated intestinal fluid (SIF) for 12 h. As detailed in Table S4, Co leaching was virtually negligible following both SGF and SIF treatments. Furthermore, the representative TEM and HAADF‐STEM images (Figure S7) demonstrated that the cobalt active sites retained their atomic dispersion without any detectable aggregation post‐exposure. These results collectively verify the robust structural integrity of Co‐SAN under harsh physiological conditions. Subsequently, we chose C57BL/6J mice to assess the intestinal retention ability of Co‐SAN in vivo by fluorescent imaging after oral administration with the fluorescent dye Cy7 affixed to Co‐SAN. Ex vivo fluorescence imaging of the excised intestinal tract revealed intense and sustained signals for up to 12 h post‐administration, unambiguously substantiating its excellent intestinal retention capacity (Figure 4a and S21a). The prolonged intestinal retention time observed throughout the entire intestinal tract is predominantly attributed to electrostatic interactions between the positively charged Co‐SAN surface and the negatively charged intestinal mucosa [36], as evidenced by Zeta potential analysis of Co‐SAN (Figure S8).

**FIGURE 4:**
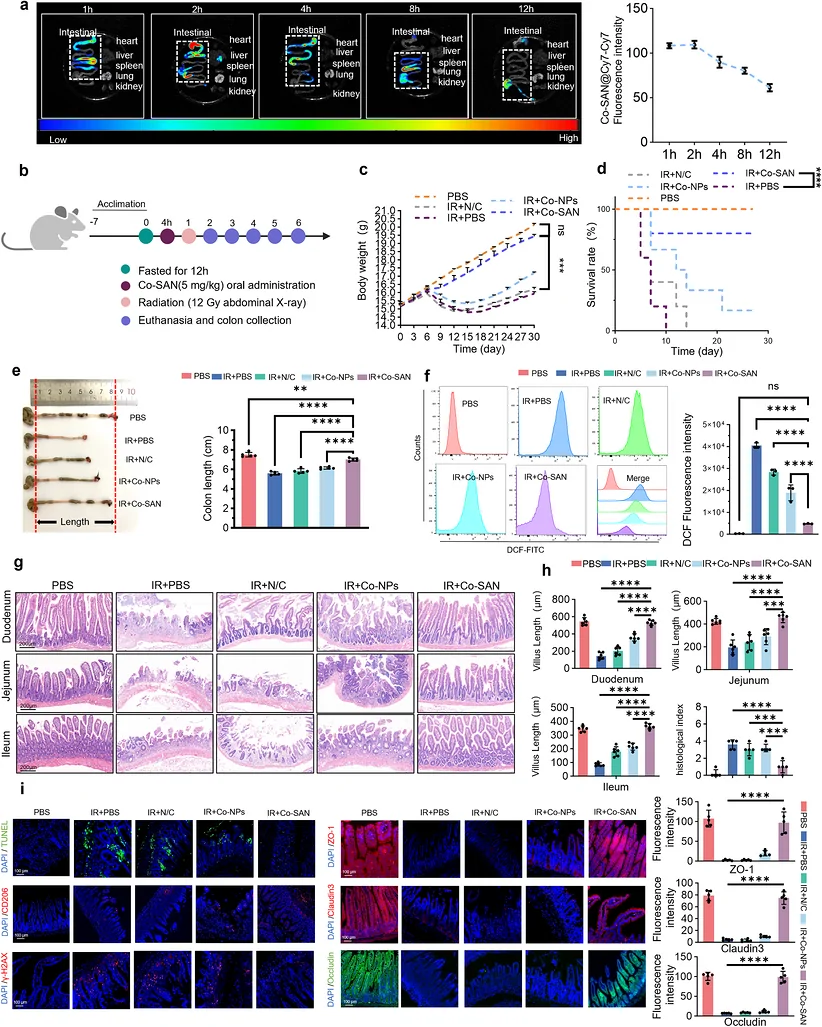
Oral Co‐SAN shows comprehensive protective effect overall intestine against radiation‐induced injury. (a) Fluorescence images of the gastrointestinal tract of mice at 1, 2, 4, 8, and 12 h after the oral administration of Co‐SAN@Cy7 (with an equal amount of Cy7). Cy7 channel: Ex, 740 nm; Em, 770 nm. The white dotted circle indicates the analyzed area for the fluorescence intensity quantification. (b) Schematic illustration of the experiment protocol. (c) Body weight of the mice (n = 3 biologically independent animals). The data show means ± SD. Data were analyzed by one‐way ANOVA followed by Dunnett's multiple comparisons test (vs. IR+Co‐SAN group). ^***^ *p* <0.001. (d) Survival curves of mice exposed to a fatal dose of abdominal 16 Gy abdominal X‐ray (n = 5 biologically independent animals). Median survival: PBS, undefined (>50 days); IR+PBS, 6 days; IR+N/C, 12 days; IR+Co‐NPs, 17 days; IR+Co‐SAN, undefined (>50 days). *P* was calculated using Log‐rank (Mantel–Cox) test. (e) Colon length of mice in each group after IR (n = 5 biologically independent animals). The data show means ± SD. “Data were analyzed by one‐way ANOVA followed by Dunnett's multiple comparisons test (vs. IR+Co‐SAN group). ^***^ *p* <0.001”. (f) Detection of ROS content in intestinal tissues between each group by flow cytometry (n = 3 biologically independent animals). The data show means ± SD. Data were analyzed by one‐way ANOVA followed by Dunnett's multiple comparisons test (vs. IR+Co‐SAN group). ^***^ *p* <0.001. (g,h) Represented HE images (g) and the (h) of the Villus Length and histologic scores in the small intestine (duodenum, jejunum, and ileum) after being treated by IR+PBS, IR+N/C, IR+Co‐NPs, and IR+Co‐SAN (n = 6 biologically independent animals). Scale bar = 200 µm. The experiment was repeated three times independently with similar results. (i) Representative IF images showing the expression of Claudin‐3 (One of the transmembrane core components of tight junctions (TJs)), Occludin (tetraspanin tight junction protein, regulate TJs.), ZO‐1(Tight junction (TJ) cytoplasmic scaffolding proteins), F4/80, γ‐H2AX, and Tunnel in the intestine in each group after IR. Scale bar = 100 µm (n = 5 biologically independent animals). The data show means ± SD. “Data were analyzed by one‐way ANOVA followed by Dunnett's multiple comparisons test (vs. IR+Co‐SAN group). ^***^ *p* <0.001”.

The radioprotective ability of Co‐SAN was evaluated using a mice acute radiation intestinal injury model (Figure 4b). Following ionizing radiation (IR) exposure, untreated mice exhibited severe weight loss and significantly shortened survival. In contrast, prophylactic administration of Co‐SAN markedly ameliorated IR‐induced weight loss and extended survival rates to levels comparable to the PBS group (Figure 4c,d). Furthermore, the radiation‐induced shortening of the colon was robustly rescued in the IR+Co‐SAN group, restoring colorectal lengths to near‐normal baseline levels (Figure 4e). Flow cytometric analysis of intestinal tissues with DCF staining demonstrated that Co‐SAN efficiently eliminated irradiation‐triggered in vivo ROS, returning ROS levels to those of the non‐irradiated PBS cohort (Figure 4f and Figure S21b). Hematoxylin‐eosin (HE) staining showed that Co‐SAN treatment preserved small intestinal villus architecture, with villus lengths and histological scores approaching those of the PBS group (Figure 4g,h). These radioprotective effects were further corroborated by Periodic acid‐Schiff (PAS) staining, which confirmed the structural preservation of mucin‐producing goblet cells (Figure S21c). To assess intestinal epithelial barrier integrity, the expression of critical tight junction proteins (ZO‐1, Occludin, and Claudin‐3) was evaluated via immunofluorescence (IF) staining. Obviously, a sharp reduction in fluorescence intensity of IR group was observed for the intestinal barrier function index, accompanied by impaired function. In contrast, the fluorescence intensity of the IR+Co‐SAN group was comparable to that of the PBS group, indicating the maintenance of excellent intestinal barrier function. Furthermore, IF staining for γ‐H2AX and TUNEL revealed a significant reduction in DNA double‐strand breaks and apoptosis, respectively, while CD206 expression suggested a favorable modulation of the local immune microenvironment, collectively halting the progression of intestinal injury (Figure 4i). Finally, enzyme‐linked immunosorbent assay (ELISA) analyses of peripheral blood and intestinal tissues demonstrated a marked downregulation of pro‐inflammatory cytokines (IL‐6, TNF‐α, and IL‐1β) in the IR+Co‐SAN cohort (Figure S21d). These comprehensive in vivo findings unequivocally establish the superior radioprotective efficacy of Co‐SAN against acute radiation enteritis.

### 2.5 Oral Administration of Co‐SAN vs. AMF in Protecting Against RIII

To further evaluate the clinical applicability of Co‐SAN, a head‐to‐head comparison with Amifostine (AMF), a clinically approved radioprotective agent, is essential. Subsequently, C57BL/6J mice were employed in our study to compare the protective efficacy of orally administered Co‐SAN vs. intraperitoneally injected AMF against acute RIII (Figure 5a). Body weight changes and survival time of mice were monitored post‐irradiation. The results indicated that both Co‐SAN and AMF significantly improved the body weight and survival rate of irradiated mice, with no statistically significant difference observed between the two groups (Figure 5b,c).

**FIGURE 5:**
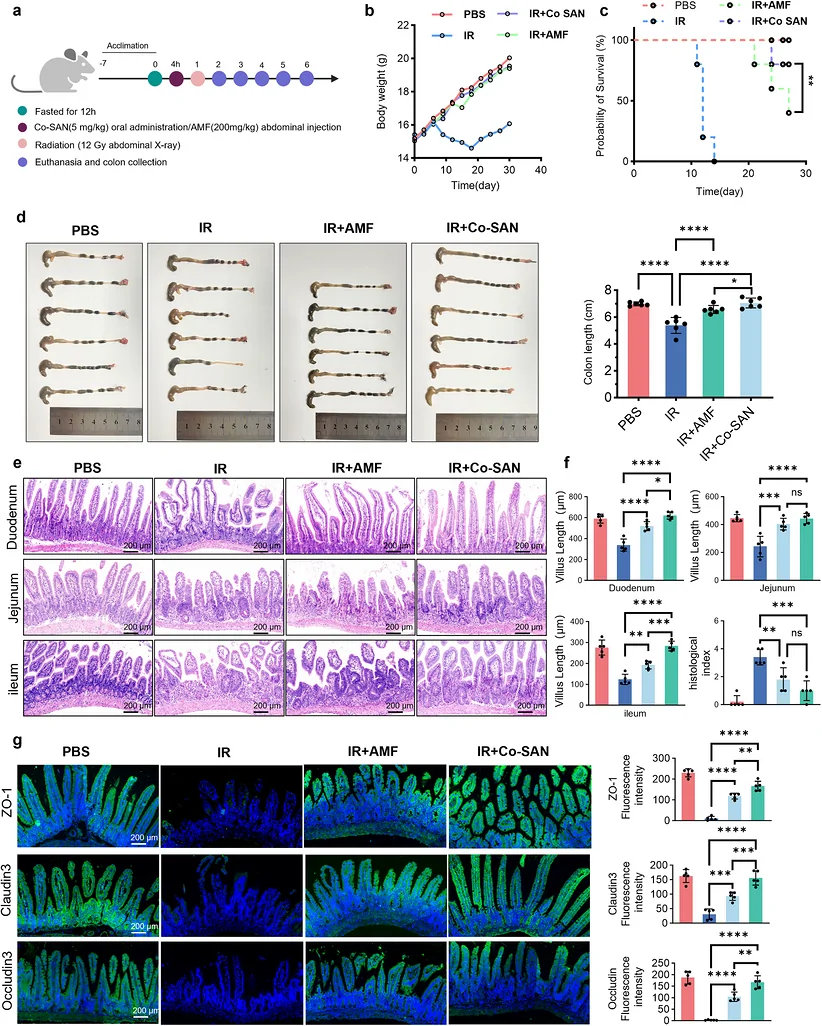
Comparison of Radioprotective Performance Between Co‐SAN and Amifostine (AMF). (a) Schematic illustration of the experiment protocol. (b) Body weight of the mice (n = 3 biologically independent animals). The data show means ± SD. Data were analyzed by one‐way ANOVA followed by Dunnett's multiple comparisons test (vs. IR+Co‐SAN group). ^***^ *p* <0.001. (c) Survival curves of mice exposed to a fatal dose of abdominal 16 Gy abdominal X‐ray (n = 5 biologically independent animals). Median survival: PBS, undefined (>50 days); IR+PBS, 12 days; IR+AMF, undefined (>50 days); IR+Co‐SAN, undefined (>50 days). *P* was calculated using Log‐rank (Mantel‐Cox) test. (d) Colon length of mice in each group after IR (n = 5 biologically independent animals). The data show means ± SD. “Data were analyzed by one‐way ANOVA followed by Dunnett's multiple comparisons test (vs. each group). ^***^ *p* <0.001”. (e,f) Represented HE images (e) and the (f) of the Villus Length and histological scores in the small intestine (duodenum, jejunum, and ileum) after being treated by IR+PBS, IR+AMF, and IR+Co‐SAN (n = 6 biologically independent animals). Scale bar = 200 µm. The experiment was repeated three times independently with similar results. (g) Representative IF images showing the expression of Claudin‐3 (One of the transmembrane core components of tight junctions (TJs)), Occludin (tetraspanin tight junction protein, regulate TJs.), ZO‐1(Tight junction (TJ) cytoplasmic scaffolding proteins), F4/80, γ‐H2AX, and Tunnel in the intestine in each group after IR. Scale bar = 100 µm. (n = 5 biologically independent animals). The data show means ± SD. “Data were analyzed by one‐way ANOVA followed by Dunnett's multiple comparisons test (vs. IR+Co‐SAN group). ^***^ *p* <0.001”.

In addition, colon length was restored in both the IR+Co‐SAN and IR+AMF groups. Notably, the degree of colon length recovery in the IR+Co‐SAN group was slightly better than that in the IR+AMF group, with statistical significance (Figure 5d). HE staining results showed that, compared with other groups, the histological scores of small intestinal tissues in the Co‐SAN group were close to those in the PBS group (Figure 5e,f). Further comparison of villus length between the Co‐SAN and AMF groups confirmed that Co‐SAN exhibited a slightly superior radioprotective effect to AMF.

At the molecular level, immunofluorescence (IF) staining was employed to assess the expression of critical tight junction proteins (ZO‐1, Occludin, and Claudin‐3), which govern intestinal barrier integrity (Figure 5g). Although irradiation precipitously downregulated these markers, both interventions effectively rescued their expression. Notably, Co‐SAN promoted a significantly more robust restoration of these barrier proteins than AMF, underscoring its enhanced capacity to maintain mucosal homeostasis.

Collectively, these compelling in vivo outcomes establish that Co‐SAN equals or outperforms the clinical standard AMF in mitigating RIII. Crucially, whereas the clinical utility of AMF is fundamentally hindered by the necessity for restrictive parenteral injections and associated systemic toxicities, the oral bioavailability of Co‐SAN offers a highly favorable, non‐invasive administration route. This paradigm‐shifting advantage promises to significantly enhance patient compliance, highlighting the tremendous potential of Co‐SAN for clinical translation in radiotherapy management.

### 2.6 Co‐SAN Affect Radiation‐Induced Neutrophil Recruitment and NETs Formation

To explore the underlying mechanism of radiation‐induced intestinal injury (RIII), single‐cell RNA (scRNA‐seq) sequencing was performed on intestinal tissues from irradiated (IR) and vehicle‐treated (PBS) mice (Figure 6a and Figure S22a). Subpopulation analysis of the immune compartment revealed that macrophages and neutrophils are the primary effectors driving RIII pathogenesis (Figure 6b). Given that ROS serve as pivotal instigators of radiation‐triggered tissue damage, neutrophils are rapidly recruited to the injury site. Upon infiltration, they secrete chemokines that exacerbate the recruitment of macrophages and other immune cells, ultimately triggering a hyperinflammatory cascade [28, 37, 38]. While neutrophil extracellular traps (NETs) perform vital physiological functions in pathogen clearance, excessive NETosis during acute inflammation exacerbates epithelial and endothelial barrier disruption, accelerating tissue injury and late‐stage fibrogenesis [39, 40, 41]. Aligning with this paradigm, Gene Ontology (GO) enrichment analysis of the neutrophil cluster demonstrated a robust enrichment in pathways associated with NET formation, inflammatory responses, and macrophage chemotaxis (Figure 6c). Furthermore, Gene Set Enrichment Analysis (GSEA) confirmed that the transcriptional signatures for NETs and inflammation [42] were markedly upregulated following irradiation (Figure 6d), exhibiting a positive correlation within the irradiated microenvironment (Figure S22b). This association was further independently corroborated by the public dataset GSE308064 [43], which revealed a significant positive correlation between ROS activity and NET formation, reinforcing our experimental findings (Figure S22c). To precisely delineate the interventional mechanisms of Co‐SAN, bulk transcriptome sequencing was subsequently conducted on the intestinal tissues. Transcriptomic profiling of neutrophil chemokine‐associated genes demonstrated a dramatic radiation‐induced upregulation, which was effectively abrogated by Co‐SAN treatment (Figure S22d,e). Consistently, Kyoto Encyclopedia of Genes and Genomes (KEGG) pathway analysis revealed that Co‐SAN significantly downregulated classical inflammatory signaling cascades, notably the TNF and NF‐κB pathways, while concurrently restoring the balance of key apoptosis‐ and metabolism‐related networks (Figure S22f,g).

**FIGURE 6:**
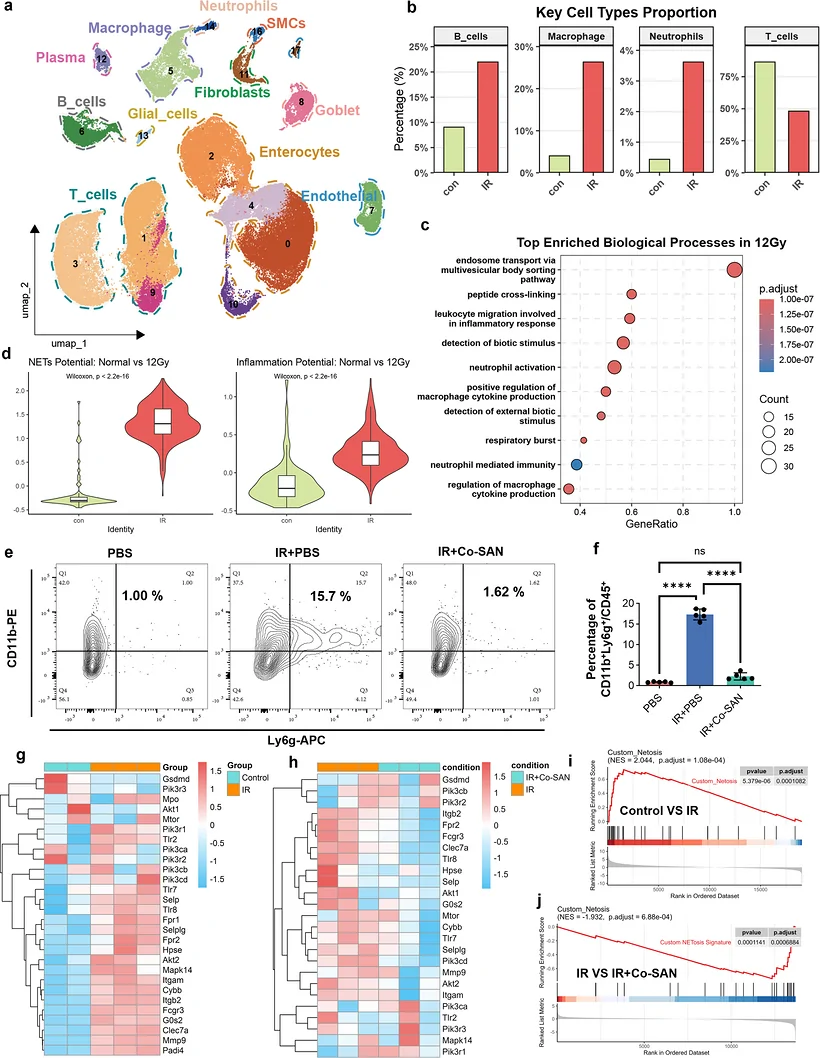
Co‐SAN affect neutrophil chemotaxis and NETs formation to attenuate injury. (a) UMAP dimensionality reduction plot of single‐cell sequencing in intestinal tissues, with cells colored by cell type annotation. (b) Single‐cell sequencing visualization dissects the composition of key immune cells in intestinal tissues. (c) GO enrichment analysis of intestinal neutrophils in mice from PBS (con) and IR groups via single‐cell analysis. (d) Violin plots of GSEA scores reflecting NETs potential and inflammatory potential of neutrophils based on single‐cell sequencing. (e,f) Flow cytometry of intestinal tissue in different groups after IR 3 days (n = 5 biologically independent animals). The data show means ± SD. Data were analyzed by one‐way ANOVA followed by Dunnett's multiple comparisons test (vs. IR group). ^***^ *p* <0.001. (g) Relative abundance heatmap of the NETs genes of the samples in different groups in genus level (data source: GSE145131). (h) Relative abundance heatmap of the NETs genes of the samples in different groups in genus level. (i) Gene Set Enrichment Analysis (GSEA) of NETs coraltion genes between the Control group and the IR group. Normalized Enrichment Score (NES) and *P* value are presented in the analysis. (j) Gene Set Enrichment Analysis (GSEA) of NETs coraltion between the IR group and the IR+Co‐SAN group. Normalized Enrichment Score (NES) and *p* value are presented in the analysis.

To further elucidate the in vivo immunomodulatory effects of Co‐SAN on neutrophils, flow cytometry was performed on intestinal tissues from the IR and IR + Co‐SAN cohorts. Co‐SAN treatment significantly curtailed radiation‐induced neutrophil recruitment (Figure 6e,f and Figure S23a). Because the co‐localization of myeloperoxidase (MPO) and citrullinated histone H3 (CitH3) serves as a canonical hallmark of NETosis, we performed immunofluorescence staining on tissue sections to evaluate this process. Compared to the irradiated controls, the IR + Co‐SAN group exhibited a dramatic reduction in MPO/CitH3 co‐localization, demonstrating that Co‐SAN effectively suppresses post‐irradiation NET formation to mitigate tissue damage (Figure S23b). Importantly, these in vivo protective mechanisms were consistently recapitulated in vitro, as corroborated by Western blot analysis of irradiated neutrophils (Figure S23c,d).

Finally, to cross‐validate our transcriptional regulatory findings, we analyzed an independent RNA‐seq dataset (GSE145131) [44]. The expression profiling of NET‐associated genes was highly consistent with our experimental observations, revealing broad upregulation following irradiation (Figure 6g). Crucially, Co‐SAN intervention successfully downregulated the majority of these pathogenic transcripts relative to the untreated IR group (Figure 6h). This confirmed that NET‐related signaling pathways were significantly enriched in irradiated intestinal tissues (Figure 6i), whereas this pathological enrichment was reversed following Co‐SAN treatment (Figure 6j).

### 2.7 The Molecular Mechanism of Co‐SAN for Preventing Radiation Damage

Having established the in vivo and in vitro radioprotective efficacy of Co‐SAN, we sought to delineate the molecular mechanisms governing its suppression of NET formation. The KEGG pathway enrichment analysis of the transcriptomic data revealed a significant enrichment of the PI3K/AKT signaling pathway (Figure 7a). GSEA results indicated the PI3K/AKT pathway was significantly up‐regulated in the IR group (Figure 7b), while it was significantly down‐regulated after Co‐SAN treatment (Figure 7c). To extrapolate the clinical relevance of this axis, we analyzed transcriptomic data from patients with systemic lupus erythematosus (SLE; GSE65391) [45], a prototypic NET‐driven autoimmune disease. This analysis revealed a strong positive correlation between PI3K/AKT pathway activation and NET‐related gene signatures (Figure 7d).

**FIGURE 7:**
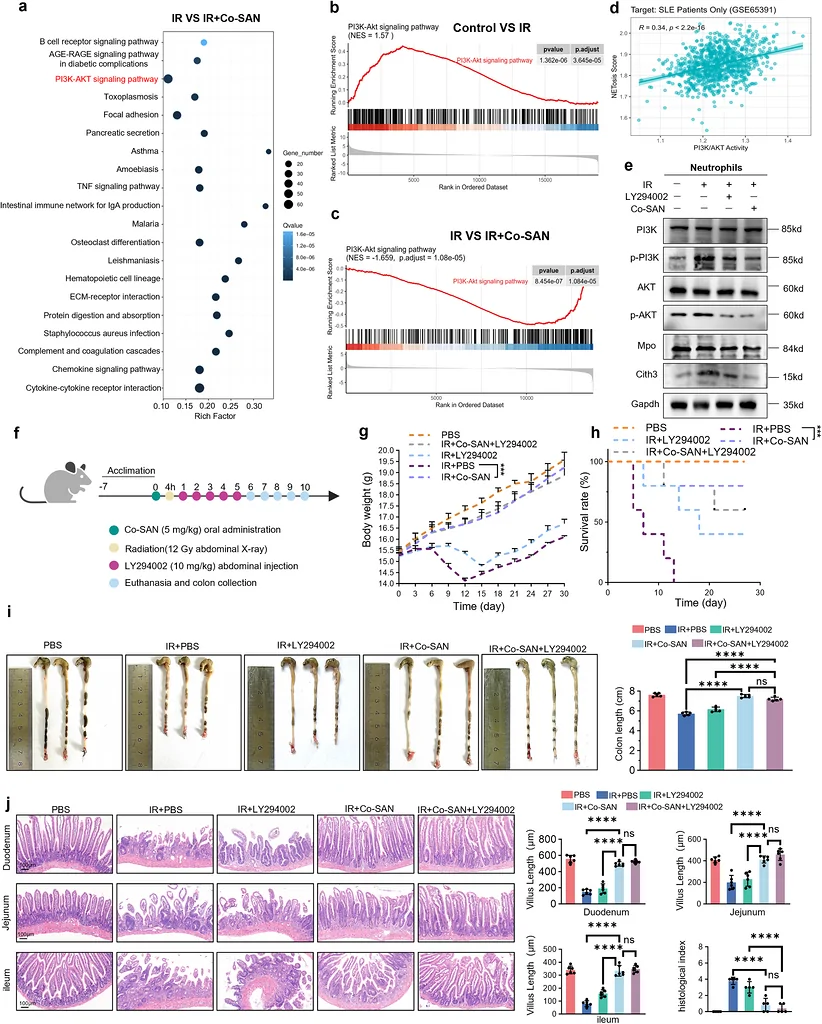
Co‐SAN ameliorate radiation induce intestinal injury by affecting the PI3K/AKT pathway. (a) Mice were orally administrated Co‐SAN with 12 Gy abdominal X‐ray (IR) and then subjected to RNA‐seq analysis. KEGG analysis shows the top 20 enriched pathways with significant differences. (b) GSEA of the changed genes in the IR group compared with the Control group. NES (normalized enrichment score) and *P* value were shown (data source: GSE145131). (c) GSEA of the changed genes after IR and Co‐SAN treatment. NES (normalized enrichment score) and *P* value were shown. (d) Correlation Analysis between PI3K/AKT Pathway Activity and NETs (Data source: GSE65391). (e) neutrophils (bone marrow‐derived cells) with 8 Gy X‐ray after treated with Co‐SAN (80 µg/ml) for 24 h or LY294002(10 µM) for 24 h, then analyzed by immunoblotting. (f) Schematic illustration of the experiment protocol. (g) Body weight of the mice (n = 3 biologically independent animals). The data show means ± SD. Data were analyzed by one‐way ANOVA followed by Dunnett's multiple comparisons test (vs. IR+Co‐SAN group). ^***^ *p* <0.001. (h) Survival curves of mice exposed to a fatal dose of abdominal 16 Gy abdominal X‐ray (n = 5 biologically independent animals). Median survival: PBS, undefined (>50 days); IR+PBS, 6 days; IR+LY294002, 15 days; IR+Co‐SAN, undefined (>50 days); IR + Co‐SAN+LY294002, undefined (>50 days). *P* was calculated using Log‐rank (Mantel–Cox) test (i) Colon length of mice in each group after IR (n = 5 biologically independent animals). The data show means ± SD. Data were analyzed by one‐way ANOVA followed by Dunnett's multiple comparisons test (vs. IR+Co‐SAN group). ^***^ *p* <0.001. (j) Represented HE images and the Villus Length in the small intestine (duodenum, jejunum, and ileum) after being treated by PBS, IR+PBS, IR+LY294002, IR+Co‐SAN, and IR+Co‐SAN+LY294002 (n = 6 biologically independent animals). Scale bar = 100 µm. The data show means ± SD. Data were analyzed by one‐way ANOVA followed by Dunnett's multiple comparisons test (vs. IR+Co‐SAN group). ^***^ *p* < 0.001.

To validate the above results, LY294002, the most widely used PI3K inhibitor, was employed in the following experiments. Western blot analysis of bone marrow‐derived neutrophils (BMDNs) confirmed that abrogating PI3K/AKT phosphorylation directly curtailed NET formation (Figure 7e and Figure S24a). Furthermore, phorbol 12‐myristate 13‐acetate (PMA) was utilized to robustly induce NETosis in neutrophils. Subsequent immunofluorescence assays revealed that both Co‐SAN and LY294002 effectively hampered NET formation. Crucially, a pharmacological rescue assay utilizing the AKT agonist SC79 successfully reversed the inhibitory effects of Co‐SAN, unambiguously confirming that Co‐SAN suppresses NETosis primarily via the PI3K/AKT axis (Figure S24b). Furthermore, scRNA‐seq was performed to dissect the dynamic state transitions of neutrophils. Quadrant analysis indicated that irradiation acts as a strong driver that propels neutrophils toward a terminal differentiation fate. In the IR group, the terminal NETs subpopulation (PI3K^−^ NETs^+^) was markedly expanded; meanwhile, the proportion of physiological‐state neutrophils (PI3K^+^ NETs^−^) was markedly depleted, and transitional‐state cells (PI3K^+^ NETs^+^) were remarkably increased (Figure S24d). This hierarchical state transition indicates an accelerated differentiation trajectory, wherein irradiated neutrophils rapidly transit through the PI3K activation phase to irreversibly commit to the NETosis program.

Thus, we further validated the role of the PI3K/AKT pathway in radiation‐induced intestinal injury in vivo (Figure 7f). Consistent with our preceding in vitro findings, prophylactic oral administration of Co‐SAN significantly ameliorated radiation‐induced weight loss and extended survival. Notably, the combination therapy (IR + LY294002 + Co‐SAN) yielded no additive benefits in body weight or survival compared to Co‐SAN monotherapy (Figure 7g,h). This phenotypic redundancy implies that Co‐SAN effectively saturates the inhibition of this specific pathogenic pathway. Similar trends were obtained in the evaluation of the colorectum length in different groups (Figure 7i). HE staining of intestinal tissue from different regions of the small intestine demonstrated that histologic scores and the length of small intestinal villi in the IR+Co‐SAN group was superior to that of the IR+LY294002 group, whereas the IR+LY294002+Co‐SAN group exhibited a similar effect to that of the IR+Co‐SAN group (Figure 7j). Similarly, immunofluorescence analysis of tight junction proteins (ZO‐1, Occludin, and Claudin‐3) and PAS staining confirmed that Co‐SAN monotherapy was sufficient to maintain mucosal barrier integrity, achieving protective outcomes equivalent to those of the combination group (Figure S25a). Importantly, the successful in vivo blockade of the PI3K/AKT pathway was confirmed by IF and Western blotting, while the reduced co‐localization of MPO and CitH3 verified the suppression of NET formation (Figure S24c and S25b). The aforementioned results illustrated the enhanced radioprotective efficacy and outstanding capacity of Co‐SAN to diminish the generation of NETs through the modulation of the PI3K/AKT pathway, thus preventing the onset of acute radiation‐induced intestinal injury.

### 2.8 Co‐SAN Protect Normal Intestinal Tissues in The Presence of Tumor

Previous in vitro and in vivo experiments demonstrated that the Co‐SAN manifested strong protective abilities to intestine against radiation‐induced injury, the protection of radiation‐induced intestinal injury under a tumor‐bearing condition should be considered. First, to investigate the effect of Co‐SAN on tumor radiotherapy, the tumor growth of the C57BL/6J mice bearing colorectal tumors was evaluated after the oral administration of Co‐SAN and abdominal X‐ray radiation (IR+Co‐SAN group) (Figure 8a). Notably, mice in the IR + Co‐SAN group exhibited a significant prolongation in survival (Figure 8b). Contrary to conferring radioprotection to neoplastic tissues, this survival benefit conclusively demonstrates that Co‐SAN sensitizes the tumor to radiotherapy. To faithfully recapitulate clinical regimens, a fractionated radiotherapy mode (5 Gy × 3) was subsequently employed, which further corroborated that Co‐SAN possesses absolutely no radioprotective effect on tumors (Figure S26a,b).

**FIGURE 8:**
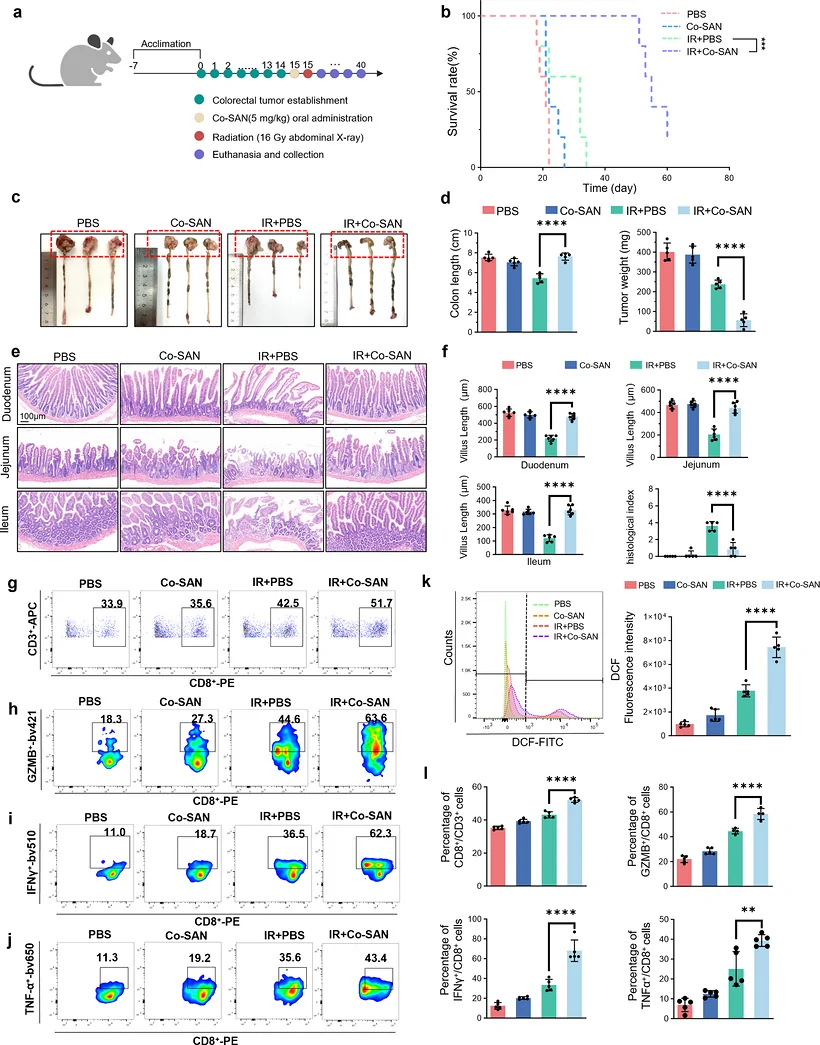
Co‐SAN increased tumor radiotherapy sensitivity while protecting normal tissues. (a) Schematic illustration of the experiment protocol. (b) Survival curves of mice after establishment of colorectal tumors (n = 5 biologically independent animals). Median survival: PBS,17 days; Co‐SAN, 19 days. IR + PBS, 27 days; IR + Co‐SAN, undefined (>50 days); *P* was calculated using Log‐rank (Mantel‐Cox) test (c,d) Colon length and tumor weight of mice in each group at 5 days post‐ by 16 Gy X‐ray (n = 5 biologically independent animals). (e,f) Represented HE images (e) and the Villus Length and histological index (f) in the small intestine (duodenum, jejunum, and ileum) after treatment with PBS (PBS group), Co‐SAN (Co‐SAN group), IR + PBS and IR + Co‐SAN (n = 6 biologically independent animals, mean ± SEM, n = 5 independent experiments, Data were analyzed by one‐way ANOVA followed by Dunnett's multiple comparisons test (vs. IR+Co‐SAN group). ^***^ *p* <0.001.). Scale bar = 100 µm. The experiment was repeated three times independently with similar results. (g‐j) Representative FACS plots and quantification of CD8^+^CD3^+^CD45^+^ cells(g); GZMB^+^CD8^+^ cells (h); IFNγ^+^CD8^+^ cells(i) and TNFα^+^CD8^+^ cells(j) in the tumor of each group (mean ± SEM, n = 5 independent experiments. (k) Detection of ROS content in tumor tissues between each group by flow cytometry (n = 5 biologically independent animals). The data show means ± SD. Data were analyzed by one‐way ANOVA followed by Dunnett's multiple comparisons test (vs. IR+Co‐SAN group). ^***^ *p* <0.001. (i) The statistical graph is shown in the Figure. (n = 6 biologically independent animals). The data show means ± SD. Data were analyzed by one‐way ANOVA followed by Dunnett's multiple comparisons test (vs. IR+Co‐SAN group). ^***^ *p* <0.001.

Crucially, comprehensive assessments of colorectal length and tumor burden revealed a dual therapeutic advantage: Co‐SAN concurrently preserved normal intestinal integrity and potentiated the tumoricidal efficacy of ionizing radiation. We postulate that this radiosensitization stems from the intrinsic, microenvironment‐responsive enzyme‐mimicking activities of Co‐SAN within the TME (Figure 8c,d). In the fractionated radiotherapy model, Co‐SAN not only extended survival but also significantly augmented tumor regression, as evidenced by macroscopic tumor reduction and H&E staining (Figure S26c,d). Given that HIF‐1α and CA9 serve as classical indicators of intratumoral hypoxia, their specific evaluation demonstrated that Co‐SAN prominently alleviated hypoxic stress, thereby overcoming a major barrier to radiosensitivity (Figure S26e and S27a). This enhanced therapeutic vulnerability was further validated by the pronounced upregulation of DNA damage markers (Figure S27b). To provide a pharmacokinetic rationale for this intratumoral efficacy, the biodistribution of cobalt was quantified via ICP‐MS (Figure S27c,d). The results showed that the blood cobalt concentration peaked at 8 h with a maximum concentration of 3.80 µg/g. Its half‐life was approximately 11 h, and the area under the concentration‐time curve (AUC) was 63.06 µg h/g. The intratumoral accumulation of cobalt single‐atom nanozyme (Co‐SAN) reached the maximum level at 24 h, with a peak concentration of 3.51 µg/g, ensuring optimal bioavailability during radiotherapy.

Moreover, the HE, PAS, and IF staining of ZO‐1, Claudin3, and Occludin indicators at various intestinal locations demonstrated the Co‐SAN recovered the intestinal barrier functions and prevent the damage of small intestinal villi even though the presence of a tumor (Figure 8e and Figure S28a). Furthermore, flow cytometric immunophenotyping of the tumor tissues revealed a robust infiltration and activation of CD8^+^ T cells in the IR + Co‐SAN group. This favorable immune remodeling was accompanied by elevated secretion of effector cytokines (GZMB, IFN‐γ, TNF‐α) and localized ROS generation (Figure 8k), culminating in significantly enhanced tumoricidal capacity (Figure 8g–l and Figure S26f,g and S28b). Mechanistically, these synergistic outcomes are attributed to the catalytic properties of Co‐SAN operating within the acidic TME, which simultaneously alleviates hypoxia and catalyzes ROS production, ultimately maximizing radiotherapeutic efficacy.

### 2.9 Protective Effect on Gut Microbiota

Given the profound vulnerability of the gut microbiome to ionizing radiation, we utilized 16S rRNA sequencing to decode the protective role of Co‐SAN. Radiation exposure induced severe compositional aberrations within the microbial community, characterized by the marked depletion of *Bacteroides* and *Actinobacteria*, alongside a concurrent enrichment of *γ‐Proteobacteria* (Figure 9a) [46, 47, 48, 49]. Remarkably, Co‐SAN administration successfully abrogated this radiation‐induced dysbiosis, maintaining a community architecture analogous to healthy controls. This structural stabilization was quantitatively validated by the recovery of α‐diversity metrics (Figure 9c) and principal coordinate analysis (PCoA) of β‐diversity, which demonstrated a clear spatial overlap between the Co‐SAN‐treated and PBS cohorts (Figure 9d). Crucially, Co‐SAN sustained the abundance of critical commensal genera, including *Lactobacillus*, *Muribaculaceae*, and *Rikenellaceae* (Figure 9b,e,f) [50−56]. These bacteria are instrumental in maintaining mucosal integrity; specifically, *Lactobacillus* and other SCFA‐producers facilitate tight‐junction assembly and barrier regulation, thereby limiting intestinal permeability [57]. The parallel microecological outcomes observed following pharmacological PI3K blockade (LY294002) reinforce the premise that the protective capacity of Co‐SAN relies, at least partially, on the suppression of inflammatory cascades. Consequently, Co‐SAN acts as a robust ecological stabilizer, defending intestinal homeostasis against radiation insults.

**FIGURE 9:**
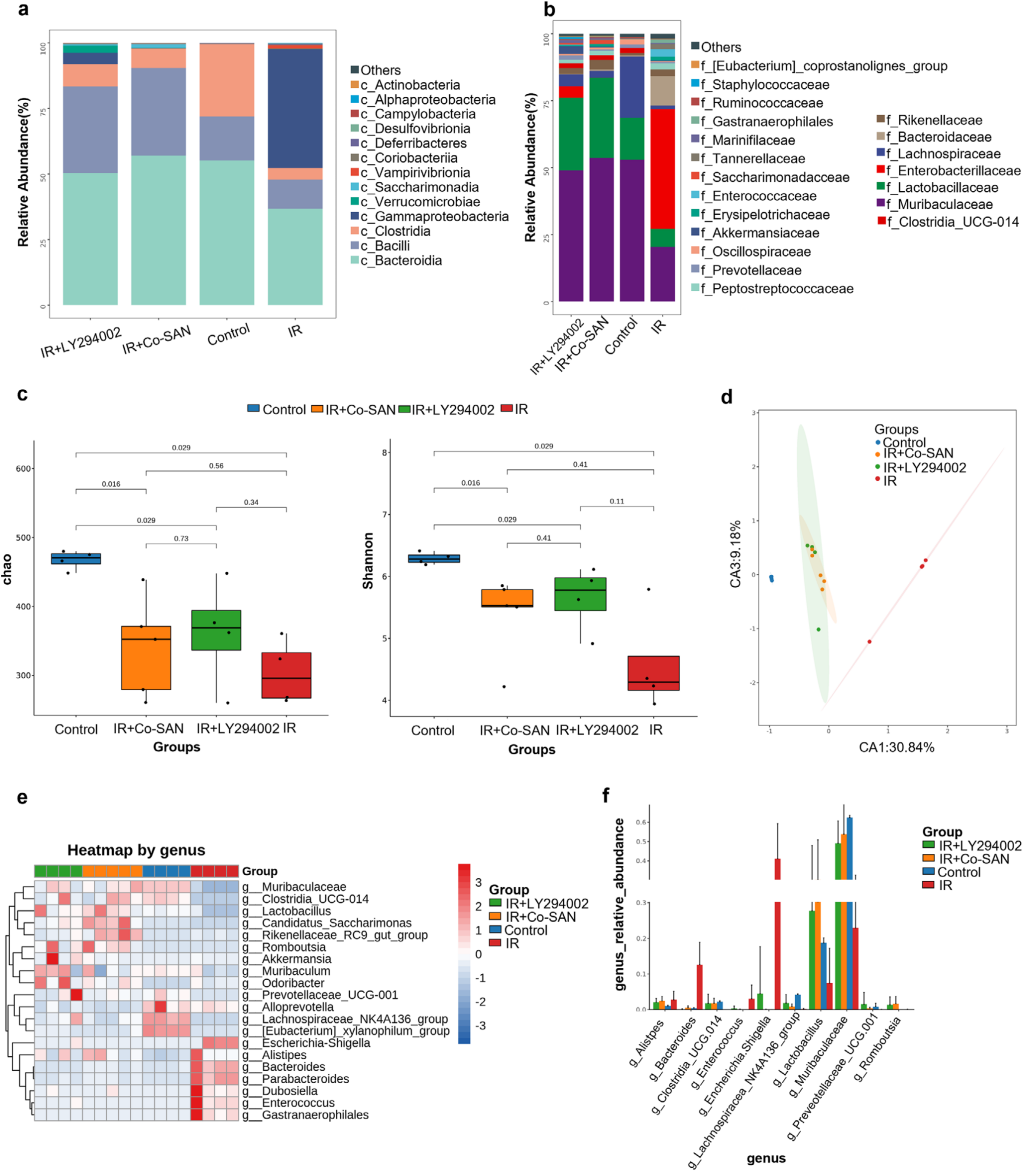
16S rRNA gene sequencing analysis shows the protective effect of Co‐SAN on the gut microbiota of irradiated mice. (a) Relative abundance barplot of the gut microbiota of the samples in different groups in family level. (b) Relative abundance barplot of the gut microbiota of the samples in different groups in genus level. (c) Alpha diversity boxplot, in which the index Chao represents the community richness, and the index Shannon represents the community diversity. n = 4 for PBS, IR, IR + LY294002 and IR+Co‐SAN (representing biologically independent animals). Results are presented as the boxes’ bounds (the 25th to 75th percentile) and lines representing maxima, medians, and minima. P was calculated using a two‐tailed t‐test. (d) Beta diversity CA (Correspondence Analysis) figure, visualizing the differences in the microbiota composition between groups through the distance in the *x*‐coordinate and *y*‐coordinate. (e) Relative abundance heatmap of the gut microbiota of the samples in different groups in genus level. (f) The analysis of significant differences of the microbiota of the samples in different groups.

To further delineate how Co‐SAN alters the intestinal metabolic microenvironment post‐irradiation, untargeted metabolomics of fecal samples was performed. Score plots from principal component analysis (PCA) and partial least squares discriminant analysis (PLS‐DA) exhibited a distinct spatial segregation between the IR and IR+Co‐SAN cohorts. This divergence signifies that Co‐SAN intervention extensively reprogrammed the radiation‐altered intestinal metabolic phenotype (Figure S29a).

Comprehensive categorization of the identified differential metabolites revealed their broad distribution across multiple chemical classes, predominantly encompassing lipids, lipid‐like molecules, organic acids, and their derivatives (Figure S29b). Subsequent functional annotation of the core differential metabolites, filtered via variable importance in projection (VIP) and fold‐change thresholds, revealed a targeted protective mechanism. Specifically, Co‐SAN treatment predominantly restored the abundance of anti‐inflammatory mediators. Metabolites conferring anti‐inflammatory and antioxidant properties—such as Citrumarin A, Cladospolide B, and Schiffnerone B—were substantially enriched (VIP > 1.66). Conversely, the levels of metabolites implicated in lipid peroxidation or pro‐inflammatory cascades, including 16‐oxo‐palmitate and 2‐hydroxymyristic acid, were significantly depleted (Figure S29c). These metabolic shifts were further corroborated by KEGG pathway enrichment and differential abundance (DA) score analyses. Co‐SAN administration profoundly upregulated cascades critical for restoring intestinal mucosal barrier function and metabolic homeostasis, notably cholesterol metabolism, bile secretion, the tricarboxylic acid (TCA) cycle, and diverse amino acid metabolic pathways (DA score > 0). Concurrently, stress‐responsive and pro‐inflammatory pathways, such as mTOR and sphingolipid signaling, were globally suppressed (DA score < 0) (Figure S29d).

In summary, Co‐SAN effectively ameliorates radiation‐induced intestinal injury by reshaping the gut microbiome, enriching anti‐inflammatory metabolites, restoring energy and lipid homeostasis, and suppressing stress‐related signaling networks, thereby comprehensively repairing the intestinal microenvironment.

### 2.10 Long‐Term Safety Profiles

Finally, to comprehensively evaluate the long‐term in vivo biosafety of Co‐SAN, continuous oral administration was performed over a 60‐day period in both naive and irradiated mouse cohorts (Figure 10a). Throughout this prolonged monitoring period, no significant body weight loss was observed in either scenario (Figure 10b). Furthermore, detailed histological examinations of the major organs revealed no discernible morphological abnormalities or pathological lesions (Figure 10c and Figure S30a). To assess potential systemic toxicity, serum biochemical markers for hepatic (ALT) and renal (UREA) functions were quantified, demonstrating that Co‐SAN induced no adverse hepatic or renal toxicity under any conditions (Figure 10d and Figure S30b). Additionally, routine hematological parameters including red blood cells (RBCs), white blood cells (WBCs), lymphocytes (Lym), and neutrophils (Neu) remained well within normal physiological ranges (Figure 10e,f). These robust biosafety profiles confirm the excellent long‐term biocompatibility of Co‐SAN, underscoring its tremendous potential for clinical translation as a safe and highly effective radioprotectant.

**FIGURE 10:**
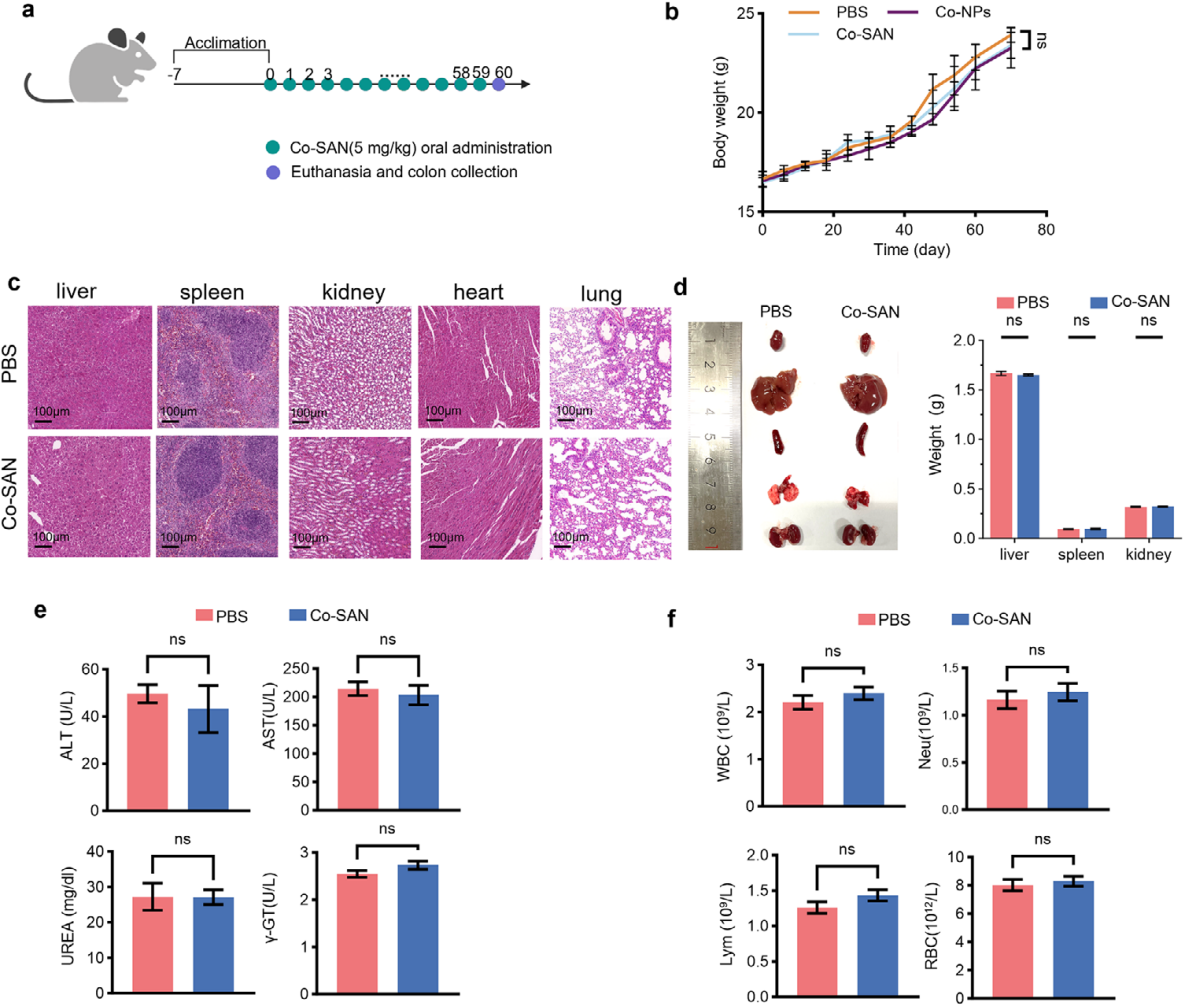
In vivo safety. (a) Schematic illustration of the experiment protocol. (b) Body weight of the mice (n = 3 biologically independent animals). The data show means ± SD. *P* between PBS group (Control) and Co‐SAN was calculated using a two‐tailed t‐test. (c) Represented HE images at day 60 after treatment with PBS (Control group), Co‐SAN group (5 mg/kg) (n = 3 biologically independent animals). Scale bar = 100 µm. The experiment was repeated three times independently with similar results. (d) Weight of vital organ of mice in the PBS group and Co‐SAN group (n = 5 biologically independent animals). *P* between the PBS group (Control) and Co‐SAN was calculated using a two‐tailed t‐test. (e‐f) Liver and kidney function (e) and complete blood counts (f) (n = 3 biologically independent animals). *P* between PBS group (Control) and Co‐SAN was calculated using a two‐tailed t‐test. (^*^<0.05, ^**^<0.01, ^***^<0.001, ns., no significance).

## 3 Conclusion

In summary, we have successfully established an oral‐administrated Co‐SAN that can response to pH in the biological microenvironment to imitate different enzymatic activities, breaking through the challenge for abdominal/pelvic tumor radiotherapy and radioprotection. The Co‐SAN presents favorable CAT, SOD, GPx catalytic activities in the environment of the intestine, thus can significantly relieve oxidative stress and protect the normal intestinal tract from radiation‐induced injury by superior ROS scavenging capability. Simultaneously, the Co‐SAN leverages CAT, OXD, NOX, and GSHOx activities in the tumor microenvironment, which can alleviate tumor hypoxia and generate ROS to improve the efficacy of radiotherapy for abdominal/pelvic tumor. Comprehensive in vitro and in vivo evaluations robustly validate this dual‐therapeutic paradigm, demonstrating exceptional biocompatibility alongside the simultaneous realization of tumor radiosensitization and normal tissue radioprotection. Mechanistically, multi‐omics combined with in vitro and in vivo experiments reveal that Co‐SAN‐mediated ROS scavenging suppresses the hyperactivation of the PI3K/AKT signaling pathway, which consequently abrogates pathogenic NET formation. This discovery delineates a critical role for NETosis in driving secondary radiation‐induced tissue damage. Ultimately, this study provides a compelling rational design for smart single‐atom nanotherapeutics, offering a highly translatable strategy to expand the therapeutic window of clinical radiotherapy.

## 4 Experimental Section

Details regarding the experimental procedures can be found in the Supporting Information. All animal experiments were approved by the Institutional Animal Care and Use Committee of Nanfang Hospital, Southern Medical University (IACUC‐LAC‐20241129‐002).

### 4.1 Statistical Analysis

All statistical analyses were performed by GraphPad Prism v.10.1 (GraphPad Software). The statistical significance was indicated as ^*^ *p* <0.05; ^**^ *p* <0.01; ^***^ *p* <0.001; ns, no significance.

## Author Contributions

**Yang Liu**: writing – original draft, validation. **Tao Li**: investigation. **Shengqi Yin**: writing – review and editing, conceptualization, software, data curation, formal analysis, visualization, methodology, writing – original draft, validation, investigation. **Junjie Li**: validation, visualization. **Zehui Zhang**: investigation. **Peiqun Yin**: investigation, conceptualization, methodology, data curation, validation, visualization, writing – review and editing, writing – original draft, funding acquisition. **Yajun Zou**: investigation. **Yilin Zheng**: investigation. **Yishu Zou**: validation, visualization, writing – original draft. **Wanying Zheng**: validation. **Jianlin Zhang**: resources, supervision, writing – review and editing. **Yi Ding**: funding acquisition, resources, writing – review and editing, methodology. **Yuen Wu**: writing – review and editing, writing – original draft, resources, supervision, funding acquisition, conceptualization, methodology. **Lu Yu**: investigation.

## Conflicts of Interest

The authors declare no conflicts of interest.

## Supporting information




**Supporting File**: advs76491‐sup‐0001‐SuppMat.pdf.

## Data Availability

The data that support the findings of this study are available from the corresponding authors upon reasonable request.

## References

[1] W. Wang , B. Cui , Y. Nie , L. Sun , and F. Zhang , “Radiation Injury and Gut Microbiota‐Based Treatment,” Protein & Cell 15 (2024): 83–97. 10.1093/procel/pwad044 PMC10833463 37470727

[2] G. Petroni , L. C. Cantley , L. Santambrogio , S. C. Formenti , and L. Galluzzi , “Radiotherapy as a Tool to Elicit Clinically Actionable Signalling Pathways in Cancer,” Nature Reviews Clinical Oncology 19 (2022): 114–131. 10.1038/s41571-021-00579-w PMC9004227 34819622

[3] Y. Pan , W. Tang , W. Fan , J. Zhang , and X. Chen , “Development of Nanotechnology‐Mediated Precision Radiotherapy for Anti‐Metastasis and Radioprotection,” Chemical Society Reviews 51 (2022): 9759–9830. 10.1039/d1cs01145f 36354107

[4] K. Wang and J. E. Tepper , “Radiation Therapy‐Associated Toxicity: Etiology, Management, and Prevention,” CA: A Cancer Journal for Clinicians 71 (2021): 437–454. 10.3322/caac.21689 34255347

[5] M. Hauer‐Jensen , J. W. Denham , and H. J. N. Andreyev , “Radiation Enteropathy—Pathogenesis, Treatment and Prevention,” Nature Reviews Gastroenterology & Hepatology 11 (2014): 470–479. 10.1038/nrgastro.2014.46 PMC4346191 24686268

[6] C. Gandle , S. Dhingra , and S. Agarwal , “Radiation‐Induced Enteritis,” Clinical Gastroenterology and Hepatology 18 (2020): A39–A40. 10.1016/j.cgh.2018.11.060 30529730

[7] A. D. Sasse , L. G. Clark , E. C. Sasse , and O. A. Clark , “Amifostine Reduces Side Effects and Improves Complete Response Rate During Radiotherapy: Results of a Meta‐Analysis,” International Journal of Radiation Oncology*Biology*Physics 64 (2006): 784–791. 10.1016/j.ijrobp.2005.06.023 16198504

[8] V. E. Kouloulias , J. R. Kouvaris , G. Pissakas , et al., “Phase II Multicenter Randomized Study of Amifostine for Prevention of Acute Radiation Rectal Toxicity: Topical Intrarectal Versus Subcutaneous Application,” International Journal of Radiation Oncology*Biology*Physics 62 (2005): 486–493. 10.1016/j.ijrobp.2004.10.043 15890591

[9] K. H. Katsanos , E. Briasoulis , P. Tsekeris , et al., “Randomized Phase II Exploratory Study of Prophylactic Amifostine in Cancer Patients Who Receive Radical Radiotherapy to the Pelvis,” Journal of Experimental & Clinical Cancer Research 29 (2010): 68. 10.1186/1756-9966-29-68 PMC2903531 20537164

[10] Y. Yang , J. Yang , J. Zhu , et al., “A DNA Tetrahedron‐Based Nanosuit for Efficient Delivery of Amifostine and Multi‐Organ Radioprotection,” Bioactive Materials 39 (2024): 191–205. 10.1016/j.bioactmat.2024.05.017 PMC11131065 38808157

[11] D. Zhang , D. Zhong , J. Ouyang , et al., “Microalgae‐Based Oral Microcarriers for Gut Microbiota Homeostasis and Intestinal Protection in Cancer Radiotherapy,” Nature Communications 13 (2022): 1413. 10.1038/s41467-022-28744-4 PMC8931093 35301299

[12] H. Guo , W. C. Chou , Y. Lai , et al., “Multi‐Omics Analyses of Radiation Survivors Identify Radioprotective Microbes and Metabolites,” Science 370 (2020): aay9097. 10.1126/science.aay9097 PMC7898465 33122357

[13] D. Zhang , J. He , J. Cui , et al., “Oral Microalgae‐Nano Integrated System Against Radiation‐Induced Injury,” ACS Nano 17 (2023): 10560–10576. 10.1021/acsnano.3c01502 37253200

[14] J. Xie , M. Zhao , C. Wang , Y. Yong , Z. Gu , and Y. Zhao , “Rational Design of Nanomaterials for Various Radiation‐Induced Diseases Prevention and Treatment,” Advanced Healthcare Materials 10 (2021): 2001615. 10.1002/adhm.202001615 33506624

[15] C. Zhang , H. Wang , X. Yang , et al., “Oral Zero‐Valent‐Molybdenum Nanodots for Inflammatory Bowel Disease Therapy,” Science Advances 8 (2022): abp9882. 10.1126/sciadv.abp9882 PMC9481133 36112678

[16] L.‐W. Xie , H.‐Y. Lu , L.‐F. Tang , et al., “Probiotic Consortia Protect the Intestine Against Radiation Injury by Improving Intestinal Epithelial Homeostasis,” International Journal of Radiation Oncology* Biology* Physics 120 (2024): 189–204. 10.1016/j.ijrobp.2024.03.003 38485099

[17] C. Peng , R. Pang , J. Li , and E. Wang , “Current Advances on the Single‐Atom Nanozyme and Its Bioapplications,” Advanced Materials 36 (2024): 2211724. 10.1002/adma.202211724 36773312

[18] L. Huang , J. Chen , L. Gan , J. Wang , and S. Dong , “Single‐Atom Nanozymes,” Science Advances 5 (2019): aav5490. 10.1126/sciadv.aav5490 PMC6499548 31058221

[19] L. Jiao , H. Yan , Y. Wu , et al., “When Nanozymes Meet Single‐Atom Catalysis,” Angewandte Chemie International Edition 59 (2020): 2565–2576. 10.1002/anie.201905645 31209985

[20] H. Zhang , X. F. Lu , Z. P. Wu , and X. W. D. Lou , “Emerging Multifunctional Single‐Atom Catalysts/Nanozymes,” ACS central science 6 (2020): 1288–1301. 10.1021/acscentsci.0c00512 PMC7453415 32875072

[21] S. Ji , B. Jiang , H. Hao , et al., “Matching the Kinetics of Natural Enzymes With a Single‐Atom Iron Nanozyme,” Nature Catalysis 4 (2021): 407–417.

[22] Y. Liu , B. Wang , J. Zhu , X. Xu , B. Zhou , and Y. Yang , “Single‐Atom Nanozyme With Asymmetric Electron Distribution for Tumor Catalytic Therapy by Disrupting Tumor Redox and Energy Metabolism Homeostasis,” Advanced Materials 35 (2023): 2208512. 10.1002/adma.202208512 36373624

[23] J. Shen , J. Chen , Y. Qian , et al., “Atomic Engineering of Single‐Atom Nanozymes for Biomedical Applications,” Advanced Materials 36 (2024): 2313406. 10.1002/adma.202313406 38319004

[24] F. Cao , L. Jin , Y. Gao , et al., “Artificial‐Enzymes‐Armed Bifidobacterium Longum Probiotics for Alleviating Intestinal Inflammation and Microbiota Dysbiosis,” Nature Nanotechnology 18 (2023): 617–627. 10.1038/s41565-023-01346-x 36973397

[25] N. Singh , G. R. Sherin , and G. Mugesh , “Antioxidant and Prooxidant Nanozymes: From Cellular Redox Regulation to Next‐Generation Therapeutics,” Angewandte Chemie International Edition 62 (2023): 202301232. 10.1002/anie.202301232 37083312

[26] J. Fox and C. K. Haston , “CXC receptor 1 and 2 nad neutrophil elastase inhibitors alter radatioinduced lung disease in the mouse,” International Journal of Radiation Oncology*Biology*Physics 85 (2013): 215–222. 10.1016/j.ijrobp.2012.02.024 22929857

[27] J. Hu , Q. Ji , F. Chen , et al., “CXCR2 is Essential for Radiation‐Induced Intestinal Injury by Initiating Neutrophil Infiltration,” Journal of Immunology Research 2022 (2022): 7966089. 10.1155/2022/7966089 PMC9308512 35879949

[28] L. M. Abernathy , M. D. Fountain , S. E. Rothstein , et al., “Soy Isoflavones Promote Radioprotection of Normal Lung Tissue by Inhibition of Radiation‐Induced Activation of Macrophages and Neutrophils,” Journal of Thoracic Oncology 10 (2015): 1703–1712. 10.1097/JTO.0000000000000677 PMC6876621 26709479

[29] F. Zhang , Y. Xia , J. Su , et al., “Neutrophil Diversity and Function in Health and Disease,” Signal Transduction and Targeted Therapy 9 (2024): 343. 10.1038/s41392-024-02049-y PMC11627463 39638788

[30] C. M. Brackett , K. F. Greene , A. R. Aldrich , et al., “Signaling Through TLR5 Mitigates Lethal Radiation Damage by Neutrophil‐Dependent Release of MMP‐9,” Cell Death Discovery 7 (2021): 266. 10.1038/s41420-021-00642-6 PMC8478872 34584068

[31] E. Nolan , V. L. Bridgeman , L. Ombrato , et al., “Radiation Exposure Elicits a Neutrophil‐Driven Response in Healthy Lung Tissue That Enhances Metastatic Colonization,” Nature Cancer 3 (2022): 173–187. 10.1038/s43018-022-00336-7 PMC7612918 35221334

[32] F. V. S. Castanheira and P. Kubes , “Neutrophils and Nets in Modulating Acute and Chronic Inflammation,” Blood 133 (2019): 2178–2185. 10.1182/blood-2018-11-844530 30898862

[33] Y. Wang , V. K. Paidi , W. Wang , et al., “Spatial Engineering of Single‐Atom Fe Adjacent to Cu‐Assisted Nanozymes for Biomimetic O_2_ Activation,” Nature Communications 15 (2024): 2239. 10.1038/s41467-024-46528-w PMC10933453 38472201

[34] J. Liu , B. Yu , M. Rong , W. Sun , and L. Lu , “A New Strategy to Fight Tumor Heterogeneity: Integrating Metal‐Defect Active Centers Within NADH Oxidase Nanozymes,” Nano Today 54 (2024): 102113.

[35] B. Yang , Y. Chen , and J. Shi , “Reactive Oxygen Species (Ros)‐Based Nanomedicine,” Chemical Reviews 119 (2019): 4881–4985. 10.1021/acs.chemrev.8b00626 30973011

[36] J. Liu , P. Leng , and Y. Liu , “Oral Drug Delivery With Nanoparticles Into the Gastrointestinal Mucosa,” Fundamental & clinical pharmacology 35 (2021): 86–96. 10.1111/fcp.12594 32749731

[37] K. O. Abulnaja , K. Kannan , A. M. K. Al‐Manzlawi , T. A. Kumosani , M. Qari , and S. S. Moselhy , “Ajwa Date Flavonoids Mitigate Neutrophil Migration and Interferon‐Γ‐Induced Renal Injury by Ultraviolet C Radiation in Rats,” Environmental Science and Pollution Research 29 (2022): 71607–71613. 10.1007/s11356-022-20956-x 35604596

[38] M. J. Moravan , J. A. Olschowka , J. P. Williams , and M. K. O'Banion , “Brain Radiation Injury Leads to a Dose‐ and Time‐Dependent Recruitment of Peripheral Myeloid Cells That Depends on CCR2 Signaling,” Journal of Neuroinflammation 13 (2016): 30. 10.1186/s12974-016-0496-8 PMC4738790 26842770

[39] V. Delgado‐Rizo , M. A. Martínez‐Guzmán , L. Iñiguez‐Gutierrez , A. García‐Orozco , A. Alvarado‐Navarro , and M. Fafutis‐Morris , “Neutrophil Extracellular Traps and its implications in inflammation: An Overview,” Frontiers in Immunology 8 (2017): 81. 10.3389/fimmu.2017.00081 PMC5292617 28220120

[40] V. Papayannopoulos , “Neutrophil Extracellular Traps in Immunity and Disease,” Nature Reviews Immunology 18 (2018): 134–147. 10.1038/nri.2017.105 28990587

[41] J. H. Nunez , C. Juan , Y. Sun , et al., “Neutrophil and Netosis Modulation in Traumatic Heterotopic Ossification,” Annals of Surgery 278 (2023): e1289–e1298. 10.1097/SLA.0000000000005940 PMC10724380 37325925

[42] Y. Zhang , L. Guo , Q. Dai , et al., “A Signature for Pan‐Cancer Prognosis Based on Neutrophil Extracellular Traps,” Journal for ImmunoTherapy of Cancer 10 (2022): 004210. 10.1136/jitc-2021-004210 PMC9189842 35688556

[43] Y. Xia , Y. Wang , Q. Xiong , et al., “Neutrophil Extracellular Traps Promote MASH Fibrosis by Metabolic Reprogramming of HSC,” Hepatology (Baltimore, Md) 81 (2025): 947. 10.1097/HEP.0000000000000762 PMC11881075 38266270

[44] W. Lu , Y. Xie , B. Huang , et al., “Platelet‐Derived Growth Factor C Signaling Is a Potential Therapeutic Target for Radiation Proctopathy,” Science Translational Medicine 13 (2021): abc2344. 10.1126/scitranslmed.abc2344 33627485

[45] J. Kong , L. Li , L. Zhimin , et al., “Potential Protein Biomarkers for Systemic Lupus Erythematosus Determined by Bioinformatics Analysis,” Computational Biology and Chemistry 83 (2019): 107135. 10.1016/j.compbiolchem.2019.107135 31751880

[46] L. Jia , Y. Jiang , L. Wu , et al., “Porphyromonas Gingivalis Aggravates Colitis via a Gut Microbiota‐Linoleic Acid Metabolism‐Th17/Treg Cell Balance Axis,” Nature Communications 15 (2024): 1617. 10.1038/s41467-024-45473-y PMC10883948 38388542

[47] R. H. Mills , P. S. Dulai , Y. Vázquez‐Baeza , et al., “Multi‐Omics Analyses of the Ulcerative Colitis Gut Microbiome Link Bacteroides Vulgatus Proteases With Disease Severity,” Nature Microbiology 7 (2022): 262–276. 10.1038/s41564-021-01050-3 PMC8852248 35087228

[48] S. Zhang , Q. Nie , Y. Sun , et al., “Bacteroides Uniformis Degrades Β‐Glucan to Promote Lactobacillus Johnsonii Improving Indole‐3‐Lactic Acid Levels in Alleviating Colitis,” Microbiome 12 (2024): 177. 10.1186/s40168-024-01896-9 PMC11414225 39300532

[49] Q. Zhao , M. Y. Dai , R. Y. Huang , et al., “Parabacteroides Distasonis Ameliorates Hepatic Fibrosis Potentially Via Modulating Intestinal Bile Acid Metabolism and Hepatocyte Pyroptosis in Male Mice,” Nature Communications 14 (2023): 1829. 10.1038/s41467-023-37459-z PMC10067939 37005411

[50] C. Li , K. Peng , S. Xiao , Y. Long , and Q. Yu , “The Role of Lactobacillus in Inflammatory Bowel Disease: From Actualities to Prospects,” Cell Death Discovery 9 (2023): 361. 10.1038/s41420-023-01666-w PMC10541886 37773196

[51] Y. J. Jang , W. K. Kim , D. H. Han , K. Lee , and G. Ko , “Lactobacillus Fermentum Species Ameliorate Dextran Sulfate Sodium‐Induced Colitis by Regulating the Immune Response and Altering Gut Microbiota,” Gut Microbes 10 (2019): 696–711. 10.1080/19490976.2019.1589281 PMC6866707 30939976

[52] B. Lamas , M. L. Richard , V. Leducq , et al., “CARD9 Impacts Colitis by Altering Gut Microbiota Metabolism of Tryptophan Into Aryl Hydrocarbon Receptor Ligands,” Nature Medicine 22 (2016): 598–605. 10.1038/nm.4102 PMC5087285 27158904

[53] Y. Zhang , F. Song , M. Yang , et al., “Gastrointestinal Dysmotility Predisposes to Colitis Through Regulation of Gut Microbial Composition and Linoleic Acid Metabolism,” Advanced Science 11 (2024): 2306297. 10.1002/advs.202306297 PMC11132037 38477534

[54] Q. W. Wang , D. J. Jia , J. M. He , et al., “Lactobacillus Intestinalis Primes Epithelial Cells to Suppress Colitis‐Related Th17 Response by Host‐Microbe Retinoic Acid Biosynthesis,” Advanced science 10 (2023): 2303457. 10.1002/advs.202303457 PMC10754072 37983567

[55] D. J. Jia , Q. W. Wang , Y. Y. Hu , et al., “Lactobacillus Johnsonii Alleviates Colitis by TLR1/2‐STAT3 Mediated CD206^+^ Macrophages IL‐10 Activation,” Gut microbes 14 (2022): 2145843. 10.1080/19490976.2022.2145843 PMC9677986 36398889

[56] F. Dong , L. Hao , L. Wang , and Y. Huang , “Clickable Nanozyme Enhances Precise Colonization of Probiotics for Ameliorating Inflammatory Bowel Disease,” Journal of Controlled Release 373 (2024): 749–765. 10.1016/j.jconrel.2024.07.064 39084465

[57] E. R. Mann , Y. K. Lam , and H. H. Uhlig , “Short‐chain Fatty Acids: Linking Diet, the Microbiome and Immunity,” Nature Reviews Immunology, 24 (2024): 577–595. 10.1038/s41577-024-01014-8 38565643

