# Supplementary material for: Cobalt Single‐Atom Nanozyme for Enhanced Intestinal Radioprotection and Tumor Radiosensitization via Bidirectional ROS Modulation
Source: Adv Sci (Weinh). 2026 Jul 7:e76491. Online ahead of print. doi: 10.1002/advs.76491 (PMC13338992; doi:10.1002/advs.76491)
Supplement: Supplementary file 1 — Supporting File: advs76491‐sup‐0001‐SuppMat.pdf. [file ADVS-9999-e76491-s001.pdf]

Supplementary Information

Cobalt Single-Atom Nanozyme for Enhanced Intestinal Radioprotection and Tumor Radiosensitization via Bidirectional ROS modulation

Shengqi Yin,[1,5,7] Junjie Li,[2,7] Yishu Zou,[1,5,7] Yang Liu,[6,7] Yilin Zheng,[1,5] Lu Yu,[1,5] Wanying Zheng,[1,5] Yajun Zou,[3] Zehui Zhang,[2] Tao Li,[2] Peiqun Yin,*[,2] Jianlin Zhang,*[,2,3] Yuen Wu,*[,4] Yi Ding*[,1,5]

[1] Center of Radiation Oncology department, Nanfang Hospital, Southern Medical University, Guangzhou, Guangdong Province, 510515, China.

[2] School of Biomedical Engineering, Research and Engineering Center of Biomedical Materials, Anhui Medical University, Hefei 230032, China.

[3] Department of Emergency Surgery, The First Affiliated Hospital of Anhui Medical University, Hefei, Anhui 230022, China.

[4] Key Laboratory of Precision and Intelligent Chemistry, University of Science and Technology of China, Hefei, 230026, China.

[5] Guangdong Province Key Laboratory of Molecular Tumor Pathology, Guangzhou, Guangdong Province,510515, China.

[6]The First Clinical Medical College of Jinan University, The First Affiliated Hospital, Jinan University, Guangzhou, Guangdong, 510630, People's Republic of China.

[7] These authors contributed equally: Shengqi Yin, Junjie Li, Yishu Zou, Yang Liu.

*Correspondence authors: Peiqun Yin (pqyin@ustc.edu.cn); Jianling Zhang (zhangjianlin@ahmu.edu.cn); Yuen Wu (yuenwu@ustc.edu.cn); Yi Ding (dy512@smu.edu.cn).

Supplementary Methods

Chemicals. Zinc nitrate hexahydrate ($Zn(NO_3)_2 \cdot 6H_2O$), Cobalt nitrate hexahydrate ($Co(NO_3)_2 \cdot 6H_2O$), 2-methyl imidazol and methanol were achieved from Sinopharm Reagent. Zinc nitrate hexahydrate ($Zn(NO_3)_2 \cdot 6H_2O$), Cobalt nitrate hexahydrate ($Co(NO_3)_2.6H_2O$), 2-methyl imidazole were obtained from Sinopharm Chemical Reagents (Shanghai, China).3,5,3',5'-tetramethylbenzidine (TMB), and 1,2-diaminobenzene (OPD), were purchased from Macklin. 1,3-diphenylisobenzofuran (DPBF), 5,5'-dithiobis-(2-nitrobenzoic acid) (DTNB), 2,2-diphenyl-1-picrylhydrazyl (DPPH), methanol (MeOH), dimethyl sulfoxide (DMSO), 5,5'-Dimethyl-1-pyrroline-N-oxide (DMPO) was acquired from Aladdin Reagent (Shanghai, China). 2,2'-azino-bis (3-ethylbenzothiazoline-6-sulfonic acid) (ABTS), NADH, NADPH were acquired from Innochem (Beijing, China). Total Superoxide Dismutase Assay Kit with WST-8, Hydrogen Peroxide Assay Kit were purchased from Beyotime Biotechnology (Shanghai, China).

Synthesis of ZIF-8, ZIF-67 and ZnCo-MOF. Regarding the synthesis of typical ZIF-8, 2-methylimidazole (1.232 g) was first dispersed in methanol solution (100 mL) and subsequently added to methanol solution (100 mL) containing $Zn(NO_3)_2 \bullet 6H_2O$ (1.116 g). The resulting mixture was sonicated for 5 min at room temperature and then aged at 37 °C overnight. The precipitate obtained from the reaction was washed three times with methanol and dried in vacuum at 65 °C.

ZIF-67 was synthesized by replacing $Zn(NO_3)_2 \bullet 6H_2O$ (1.116 g) with $Co(NO_3)_2 \bullet 6H_2O$ (1.0 g) using a similar synthetic method as the synthesis of ZIF-8.

To synthesize ZnCo-MOF, $Zn(NO_3)_2 \bullet 6H_2O$ (1.116 g) and $Co(NO_3)_2 \bullet 6H_2O$ (0.5 g) were dispersed in methanol solution (100 mL), followed by sonication at room temperature and simultaneous injection of methanol solution containing 2-methylimidazole (1.232 g, 100 mL) and allowed to stand at 37 °C for 6 h. The precipitate was washed three times with methanol and dried under vacuum at 65 °C.

Synthesis of NC, Co-NPs and Co-SAN. The vacuum-dried ZIF-8, ZIF-67 and ZnCo-MOF powders were placed in a tube furnace and then increased to 900 °C for 3 h with a temperature increase rate of 5 °C/min under the protection of a flowing N_2 atmosphere. The obtained samples were cooled to room temperature to collect NC, Co-NPs and Co-SAN, respectively.

Characterization. Transmission electron microscopy (TEM) images were taken by Hitachi-7700 at 100 kV. Scan electron microscopy (SEM) images were taken by JSM-6700F. Using a JEOL JEM-ARM 200F high-angle annular dark field scanning transmission electron microscopy (HAADF-STEM) with a

spherical aberration corrector, the morphology of the sample was characterized on the atomic scale under the working voltage of 200 keV. X-ray diffraction (XRD) patterns were recorded by DX-2700BH. The Optima 7300 DV was used for inductively coupled plasma atomic emission spectrometry (ICP-AES) measurements. X-ray photoelectron spectroscopy (XPS) were carried out at the Catalysis and Surface Science Terminal Station at the BL11U beamline at the National Synchrotron Radiation Laboratory (NSRL) in Hefei. Using a Micrometics ASAP 2020 Nitrogen Physical Adsorption Desorption Apparatus, the samples' specific surface area and pore size were evaluated after being degassed for 6 hours at 573.15 K. Co K-edge X-ray absorption fine structure (XAFS) spectrograms were recorded at the 1W1B test station of the Beijing Synchrotron Radiation Facility (BSRF). The XAFS data were processed according to the standard procedures using the Athena module implemented in the IFEFFIT software packages. The EXAFS spectra were obtained by subtracting the post-edge background from the overall absorption and then normalizing with respect to the edge-jump step. Subsequently, the $\chi(k)$ data of were Fourier transformed to real (R) space using a hanning windows (dk = 1.0 $Å^{-1}$) to separate the EXAFS contributions from different coordination shells. To obtain the quantitative structural parameters around central atoms, least-squares curve parameter fitting was performed using the ARTEMIS module of IFEFFIT software packages.

Antioxidant properties test. The antioxidant capacity of Co-SAN was detected by 2,2-diphenyl-1-picrylhydrazyl (DPPH). 80 μL of Co-SAN (1 mg/mL) and the same concentrations of NC and Co-NPs were added to 920 μL of DPPH· solution (50 μg/mL) and incubated for 30 min, and then the absorbance was measured at 519 nm.

The antioxidant property of Co-SAN at different pH values was tested by 2,2'-azino-bis(3-ethylbenzothiazoline-6-sulfonic acid) (ABTS). 80 μL of Co-SAN (1 mg/mL) was added to 920 μL of $ABTS^{\bullet+}$ buffer solution at different pH (7.4, 7.8, 8.4, and 9.0) and incubated for 5 min, and then the absorbance at 734 nm was measured.

To test the stability of Co-SAN, Co-SAN were dispersed in pH 1.2 buffer and incubated for 3 h. After washing with deionized water for three times, their antioxidant properties were further tested by ABTS.

Catalase-mimic activity of Co-SAN. The CAT-mimicking activity of Co-SAN was explored by testing the ability of the samples to catalyse the decomposition of H_2O_2 for oxygen production. Immediately after the addition of Co-SAN (2.5 μg/mL) to a buffer (20 mM, pH 7.8) containing hydrogen peroxide (50 mM), the production of oxygen was monitored using a dissolved oxygen detector (JPSJ-606L, Leici

China).

The rate of hydrogen peroxide inhibition by Co-SAN at different concentrations and the pH dependence of CAT-mimic activity were examined by a hydrogen peroxide assay kit (Beyotime Biotechnology, China). The kinetics of Co-SAN with H_2O_2 as substrate was determined by varying the concentration of H_2O_2 solution in the reaction solution (0, 5, 10, 20, 30, 40, 50 mM). The decomposition of H_2O_2 was measured by recording the change in absorption at 240 nm (ε= 43.6 M^{-1} cm^{-1}).

Superoxide-dismutase-mimic activity of Co-SAN. The rate of superoxide anion ($O_2^{\bullet-}$) inhibition at different concentrations of Co-SAN was further examined by the total SOD activity assay kit (Beyotime Biotechnology, China).

Glutathione peroxidase- and oxidase-mimic activity of Co-SAN. The GPx-mimic activity of Co-SAN was detected by 5,5'-dithiobis-(2-nitrobenzoic acid) (DTNB) probe assay. Reaction solutions containing glutathione (2 mM), H_2O_2 (0.1 mM, 0.5 mM and 1 mM) and Co-SAN (80 μg/mL) were prepared in buffer (20 mM, pH 7.8). The absorbance at 420 nm was measured by adding DTNB after incubation for 30 min in a dark environment. In addition, by adjusting the buffer pH (7.4, 7.8, 8.4, and 9.0), the pH dependence of GPx-mimic activity was further investigated.

The GSHOx-mimic activity of Co-SAN was assayed by a similar experimental method without the addition of H_2O_2 solution.

Nicotinamide adenine dinucleotide (phosphate) oxidase-mimic of Co-SAN. The NOX-mimic activity of Co-SAN was detected by the absorbance change of NAD(P)H at 340 nm. Co-SAN (80 μg/mL) were added to a buffer containing NADH (200 μg/mL) at different pH values for the reaction, and the absorbance of the reactants at 260 nm and 340 nm was measured. The H_2O_2 generated during the oxidation reaction of NADH at different concentrations was then detected using a hydrogen peroxide detection kit. The experiment of NADPH oxidation was also determined by the same method as NADH.

Peroxidase-mimic and oxidase-mimic of Co-SAN. The OXD-mimic activity of Co-SAN was detected by 1,2-diaminobenzene (OPD) chromogenic assay. Co-SAN (80 μg/mL) were added to a buffer (20 mM, pH 6.0) containing OPD (1 mM) for reaction, and the absorbance at 417 nm was measured. The $O_2^{\bullet-}$ produced by oxidation at different times was detected by 1,3-diphenylisobenzofuran (DPBF). The kinetics of Co-SAN with TMB as substrate was determined by varying the concentration of TMB solution in the reaction solution (0, 0.09375, 0.1875, 0.375, 0.75, 1.125, 1.5 mM). The oxidation of TMB was measured by recording the absorption change at 652 nm (ε= 39000 M^{-1} cm^{-1}).

The POD-mimic activity of Co-SAN was evaluated using a 3,5,3',5'-tetramethylbenzidine (TMB) chromogenic assay. The reaction solution containing Co-SAN (80 μg/mL), H_2O_2 (0.1 mM) and TMB (0.5 mM) was prepared in buffer (20 mM, pH 5.0) and the absorbance at 652 nm was measured. The kinetic analysis of the POD-mimicking activity was performed following the same protocol as that used for the OXD-mimicking kinetic assay.

In addition, the concentration and pH dependence of Co-SAN POD-mimic and OXD-mimic activities were examined by varying the concentration of Co-SAN in the reaction system (5, 10, 20, 40, and 80 μg/mL) as well as the pH of the buffer (4.0, 5.0, 6.0, 6.8, and 7.4).

•OH and $O_2^{\bullet-}$ detection. The generation of •OH and $O_2^{\bullet-}$ was detected by electron paramagnetic resonance (ESR). To detect •OH, a reaction solution containing Co-SAN (80 μg/mL), H_2O_2 (1 mM) and DMPO (0.1 mM) was prepared in buffer (20 mM, pH 6.0). To prevent $O_2^{\bullet-}$ interference in the reaction solution superoxide dismutase was added. The reaction solution was sonicated and then analyzed using EMXplus (Bruker). ESR analysis was performed. To probe the generation of $O_2^{\bullet-}$, Co-SAN (80 μg/mL) were added to a buffer (20 mM, pH 6.0) containing NADPH (200 μg/mL) and DMPO (0.1 mM), and the ESR spectra of the reaction solution were measured after the addition of DMSO to remove the interference of •OH in the experiment.

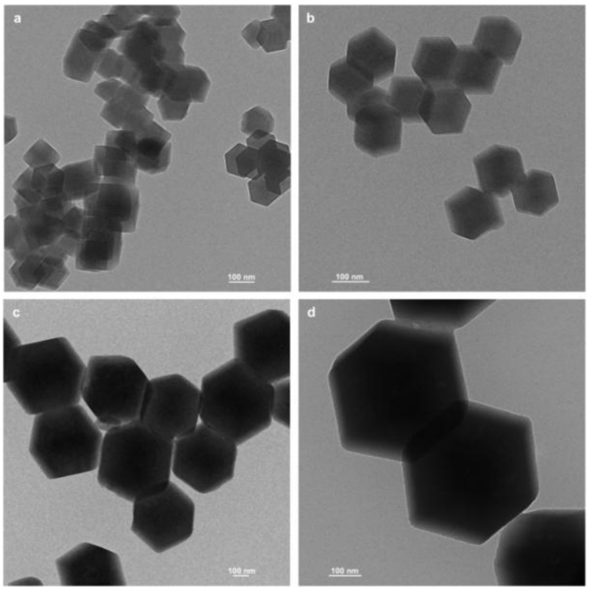

Supplementary Fig. 1 Morphology characterization of ZnCo-MOF and ZIF-67. Representative TEM images of (a-b) ZnCo-MOF and (c-d) ZIF-67 (The experiments were repeated three times with similar results).

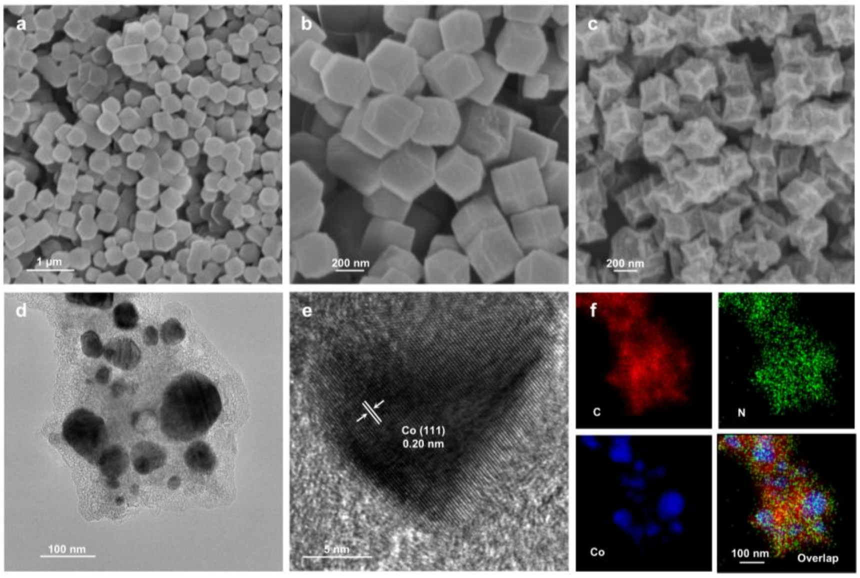

Supplementary Fig. 2 Physicochemical structural characterization of ZIF-67 and Co-NPs derived from ZIF-67. Representative SEM images of (a-b) ZIF-67 and (c) Co-NPs. (d) HRTEM image of Co-NPs. (e) HRTEM image of Co-NPs with clear lattice fringes. (f) EDS mapping images of Co-NPs. (The experiments were repeated three times with similar results).

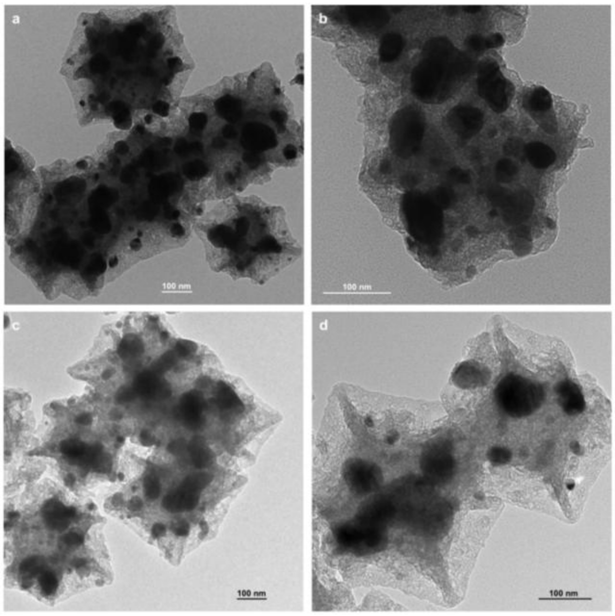

Supplementary Fig. 3 (a-d) TME images and (c-d) HRTEM images of Co-NPs. (The experiments were repeated three times with similar results).

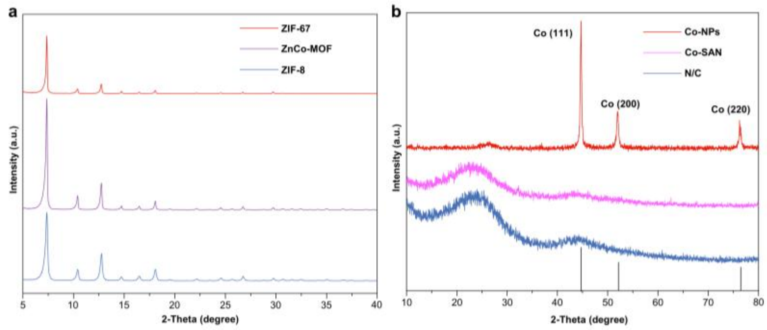

Supplementary Fig. 4 XRD patterns of ZIF-8, ZIF-67, and ZnCo-MOF and the corresponding samples after pyrolysis of the samples. Source data are provided as a Source Data file.

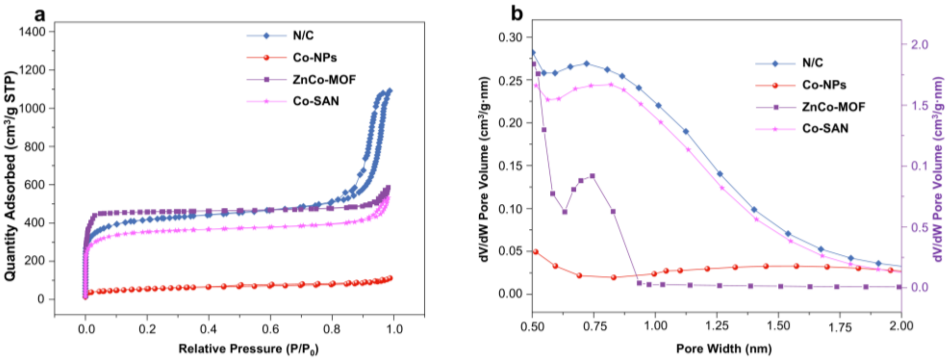

Supplementary Fig. 5 N_2 adsorption−desorption isotherms of (a) N/C, Co-NPs, ZnCo-MOF, and Co-SAN, and (b) the corresponding pore width distribution. Source data are provided as a Source Data file.

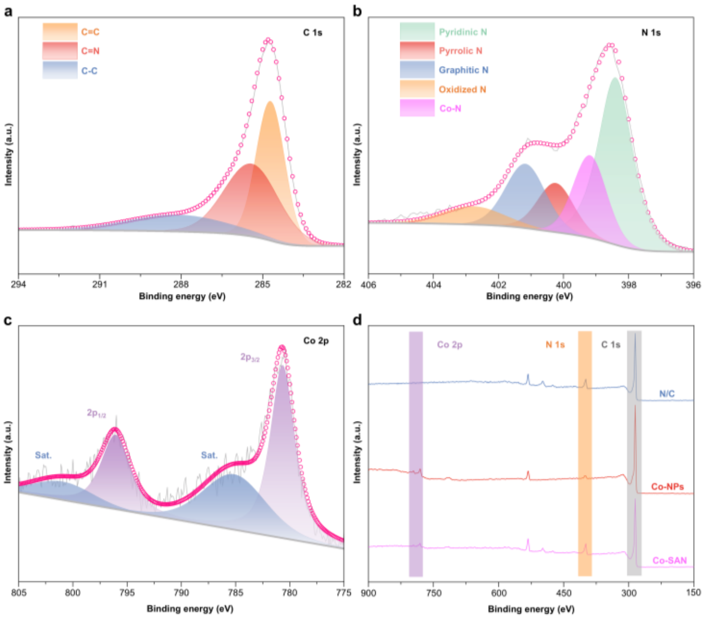

Supplementary Fig. 6 XPS High-resolution spectra of (**a**) C 1s, (**b**) N 1s and (**c**) Co 2p regions of Co-SAN. (**d**) XPS survey spectra of N/C, Co-NPs, and Co-SAN. Source data are provided as a Source Data file.

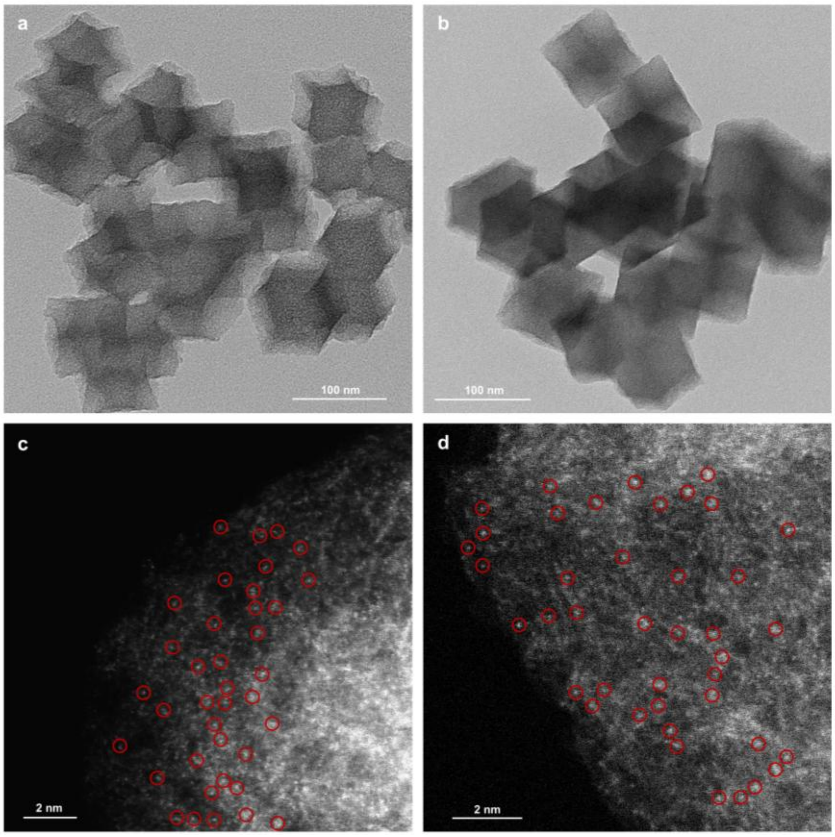

Supplementary Fig. 7 TEM images of Co-SAN. (a,c) TEM image and aberration-corrected HAADF-STEM image of Co-SANs after incubation with SGF for 1 h. (b,d) TEM image and aberration-corrected HAADF-STEM image of Co-SAN after incubation with SIF for 12 h.

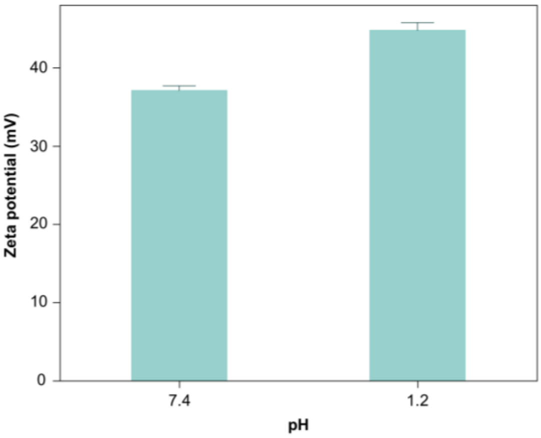

Supplementary Fig. 8 Zeta potential of Co-SAN dispersed in pH 7.4 and pH 1.2 buffers for 3 h. Data are presented as mean values ± SD (n = 3). Source data are provided as a Source Data file.

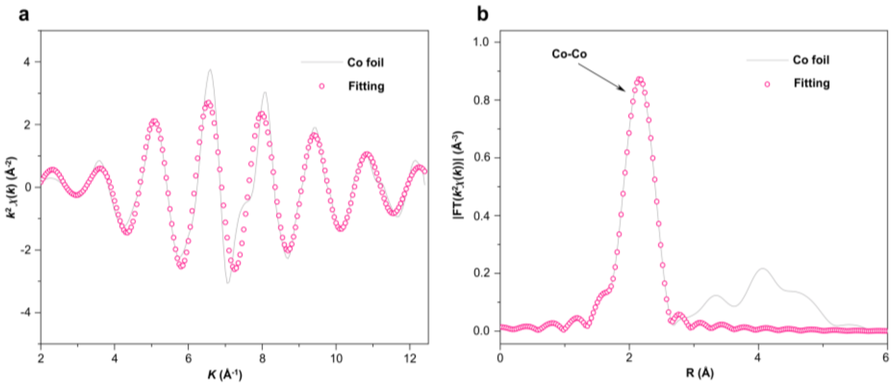

Supplementary Fig. 9 EXAFS fitting curves of (a-b) Co foil in *k* space and R space, respectively. Source data are provided as a Source Data file.

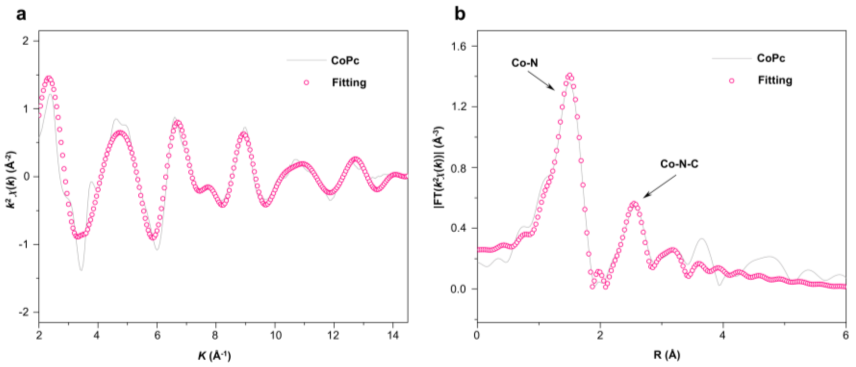

Supplementary Fig. 10 EXAFS fitting curves of (a-b) CoPc in k space and R space, respectively. Source data are provided as a Source Data file.

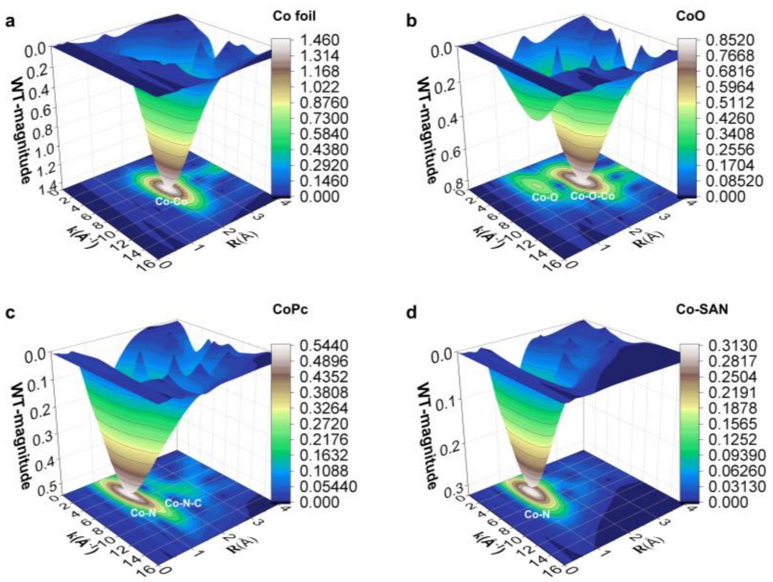

Supplementary Fig. 11 The corresponding Wavelet transform plots of the EXAFS of Co foil, CoO, CoPc and Co-SAN, respectively.

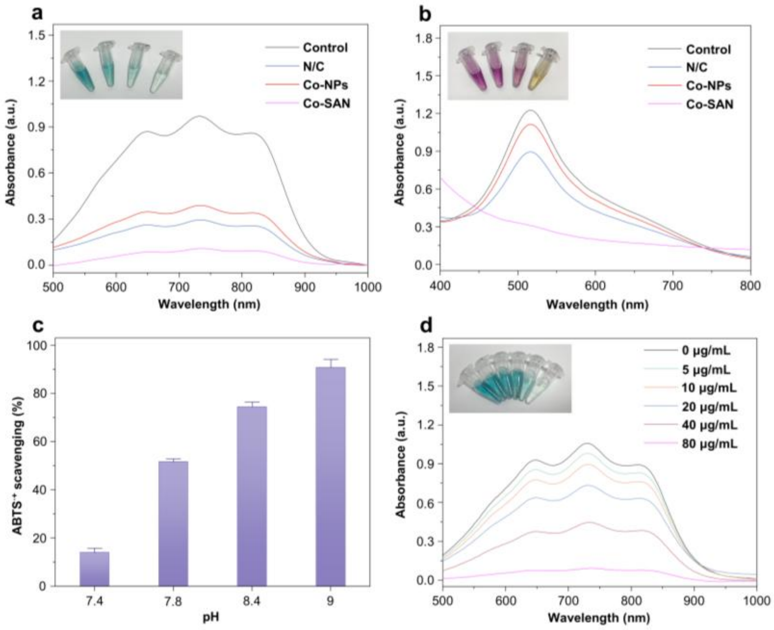

Supplementary Fig. 12 Detection of antioxidant properties of different samples. Comparison of the effects of the same concentrations of N/C, Co-NPs and Co-SAN scavenging effects (a) $ABTS^{\bullet+}$ and (b) DPPH•. (c) Co-SAN antioxidant capacity versus pH value (7.4, 7.8, 8.4, 9.0). Data are presented as mean values ± SD (n = 3). (d) Co-SAN was dispersed in pH 1.2 buffer for 3 h, and the effect of different concentrations of Co-SAN in scavenging $ABTS^{\bullet+}$ was detected. Source data are provided as a Source Data file.

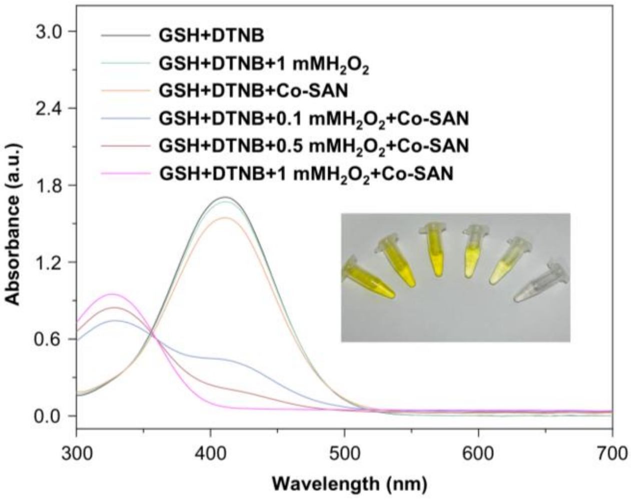

Supplementary Fig. 13 GPx-mimic catalytic activity of Co-SAN. Source data are provided as a Source Data file.

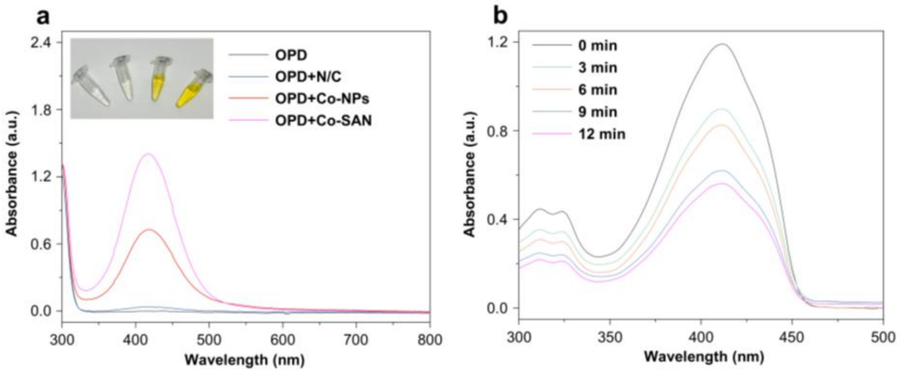

Supplementary Fig. 14 OXD-mimic catalytic activity of Co-SAN. Source data are provided as a Source Data file. (a) OXD-mimic activity of N/C, Co-NPs and Co-SAN. (b) Time-Dependent $O_2^{\bullet -}$ Generation Evaluation for Co-SAN. Source data are provided as a Source Data file.

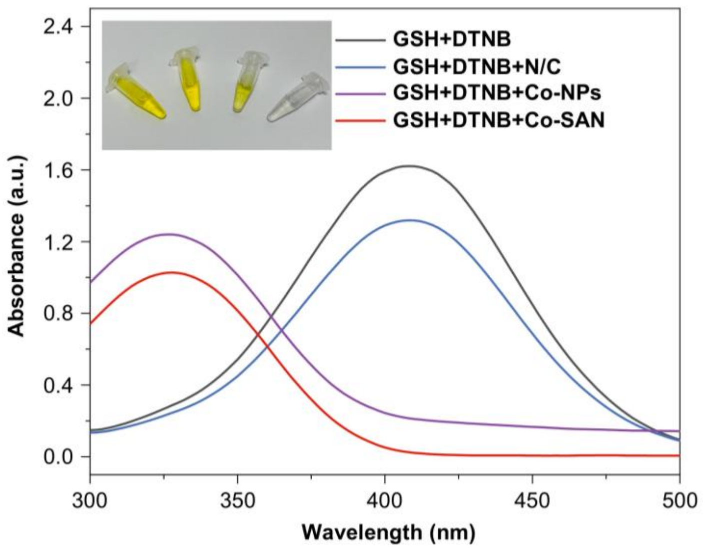

Supplementary Fig. 15 GSHOx-mimic catalytic activity of N/C, Co-NPs and Co-SAN. Source data are provided as a Source Data file.

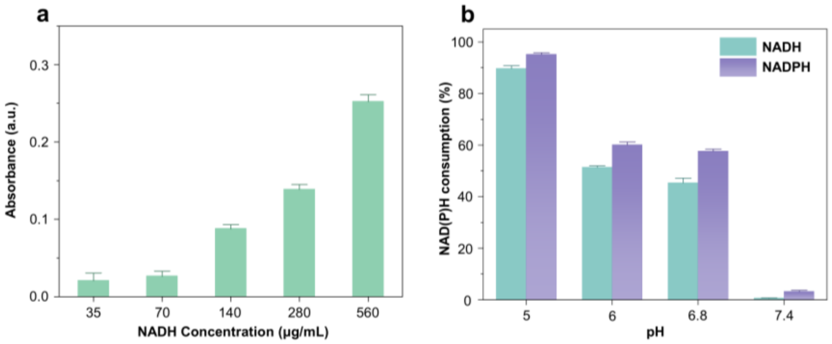

Supplementary Fig. 16 NOX-mimic catalytic activity of Co-SAN. (a) Hydrogen peroxide generation by co-incubation of different concentrations of NADH with Co-SAN as assessed by a hydrogen peroxide assay kit. Data are presented as mean values ± SD (n = 3). (b) Detection of the ability of Co-SAN to consume NAD(P)H under different pH conditions. Data are presented as mean values ± SD (n = 3). Source data are provided as a Source Data file.

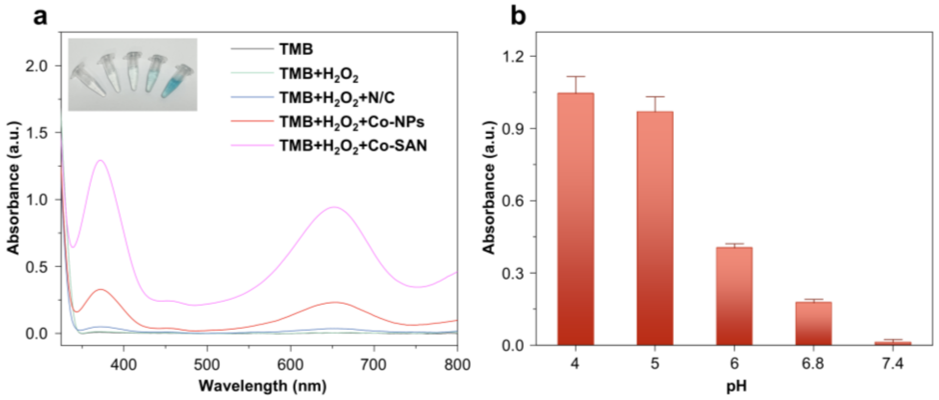

Supplementary Fig. 17 POD-mimic catalytic activity of Co-SAN. (a) UV/Vis spectra of different solutions for TMB oxidation. (b) Comparison of POD-mimic activity of Co-SAN under different pH conditions. Data are presented as mean values ± SD (n = 3). (b) •OH generation detected by ESR spectra. Source data are provided as a Source Data file.

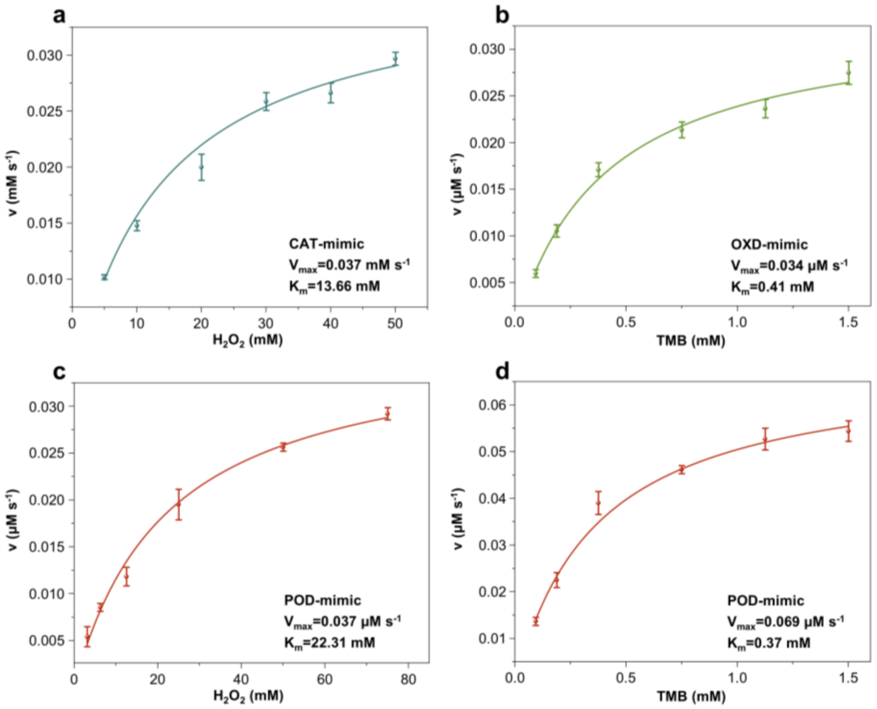

Supplementary Fig. 18 Michaelis–Menten kinetic analysis for Co-SAN of the (a) CAT-mimic reaction with H2O2 substrates, (b) OXD-mimic reaction with TMB substrates, (c-d) POD-mimic reaction with TMB and H2O2 substrates. Data are presented as mean values ± SD (n = 3). Source data are provided as a Source Data file.

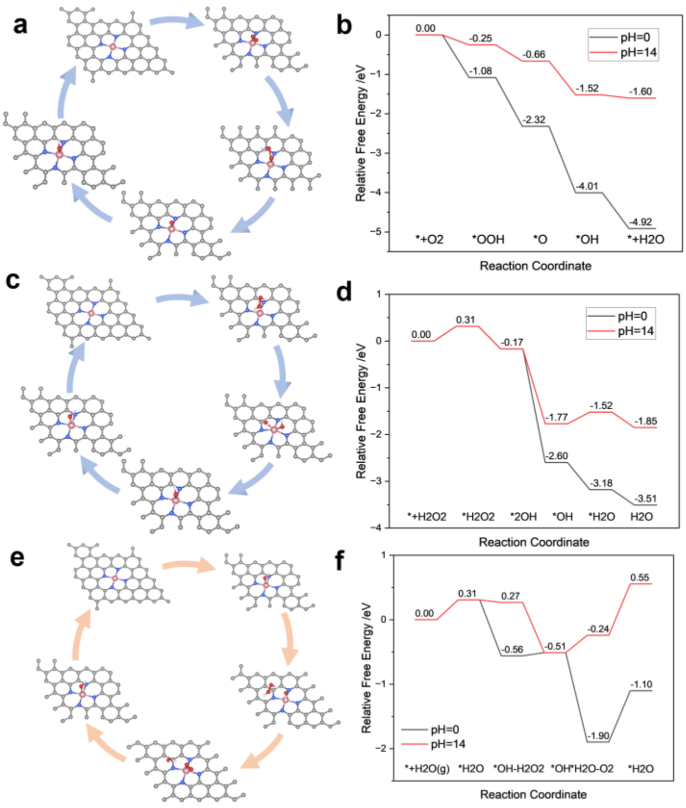

Supplementary Fig. 19 DFT calculations of the enzyme-like catalytic mechanisms of Co-SAN. (a) Proposed reaction pathways with optimized adsorption configurations (a) and corresponding free energy diagrams (b) for the oxidase-like (OXD) activity of Co-SAN under acidic (pH = 0, black line) and alkaline (pH = 14, red line) conditions. (c) Proposed reaction pathways and corresponding free energy diagrams (d) for the peroxidase-like (POD) activity. (e) Proposed reaction pathways and corresponding free energy diagrams (f) for the superoxide dismutase-like (SOD) activity.

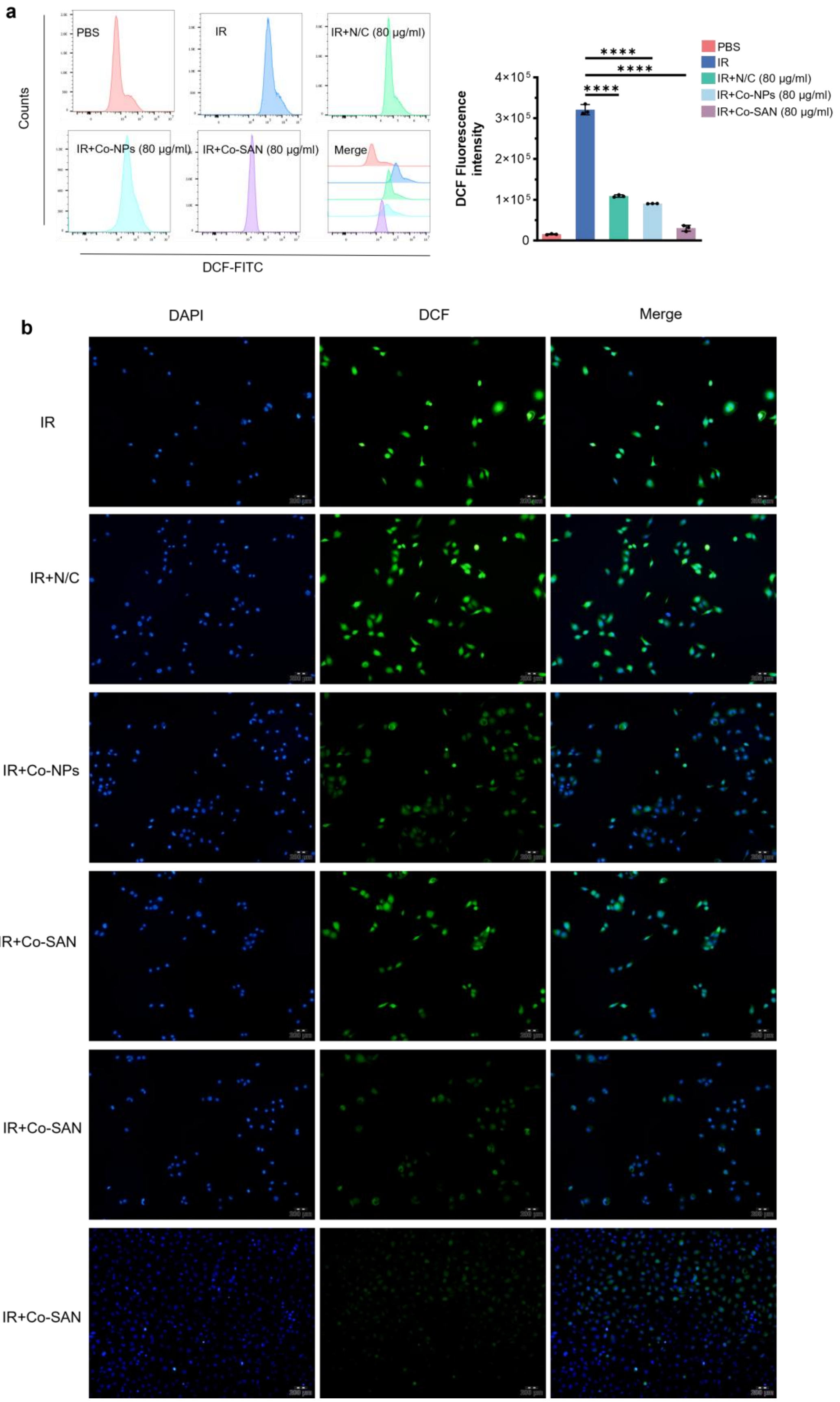

Supplementary Fig. 20. ROS detection in cellular radiotherapy. (a) ROS intensity after radiotherapy in NCM460 by flow assay, demonstrating the good and superior ROS scavenging capability of Co-SAN. (b) Confocal fluorescent images of ROS levels (green,

DCF; blue, Hoechst) in the ICE-6 cells irradiated by 8 Gy X-ray (IR) after 2h of incubation with the renewed medium in different groups. Scale bar = 200 µm.

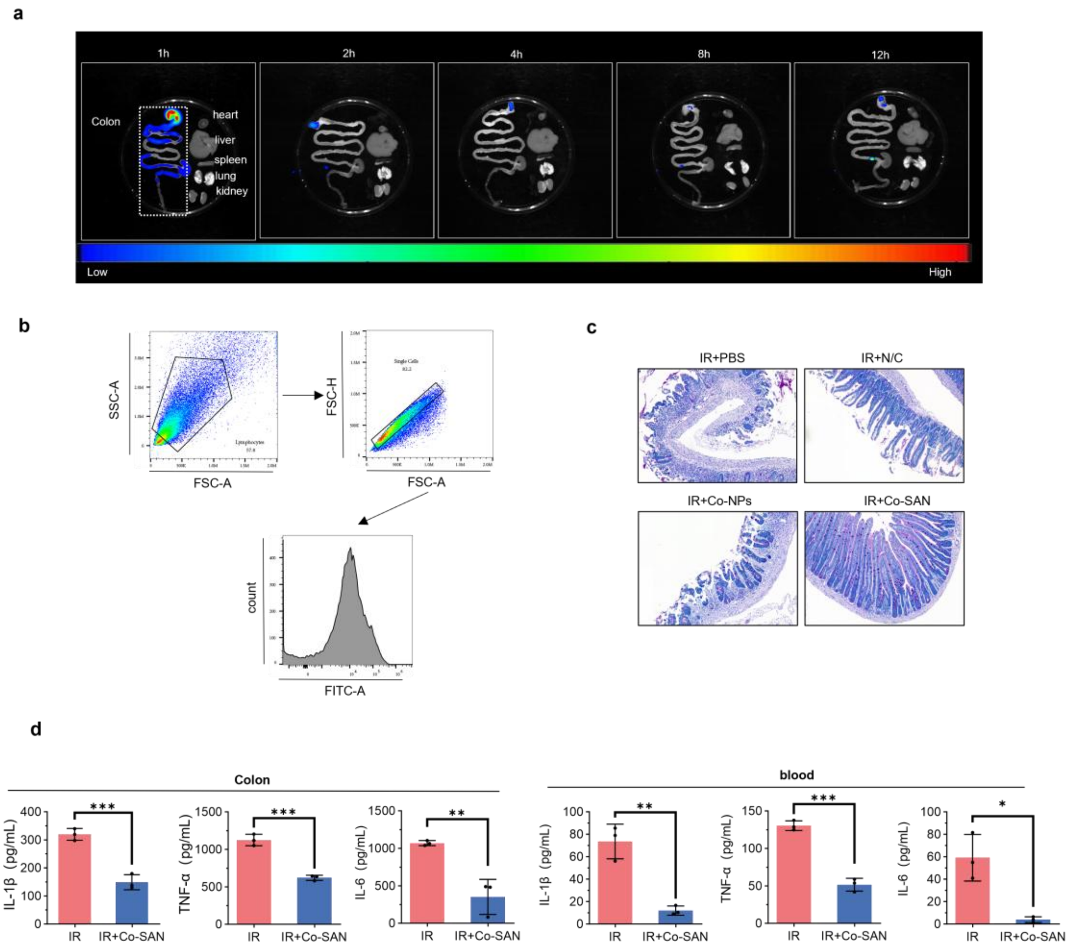

Supplementary Fig. 21. Detection of inflammatory factors in plasma and tissues. (a) Fluorescence images of the mice's gastrointestinal tract at 1, 2, 4, 8, and 12 h after the oral administration of Cy7 (with an equal amount of Cy7@Co-SAN). Cy7 channel: Ex, 740 nm; Em, 770 nm. The white dotted circle indicates the analyzed area for the fluorescence intensity quantification. (b) Example of a flow-through trap door. (b) Example of a flow-through trap door. (c) Representative PAS in the intestine in each group at 5 days after IR. (n = 5 biologically independent animals). (d) Pro-inflammatory cytokines including IL-1β, IL-6, and TNF-α in the small intestine tissue and the supernatant of peripheral blood (n = 3 biologically independent animals). The data show means ± SD. *P* was calculated using two-tailed t-test. **P* versus IR + PBS group (*<0.05, **<0.01, ***<0.001, ns, no significance).

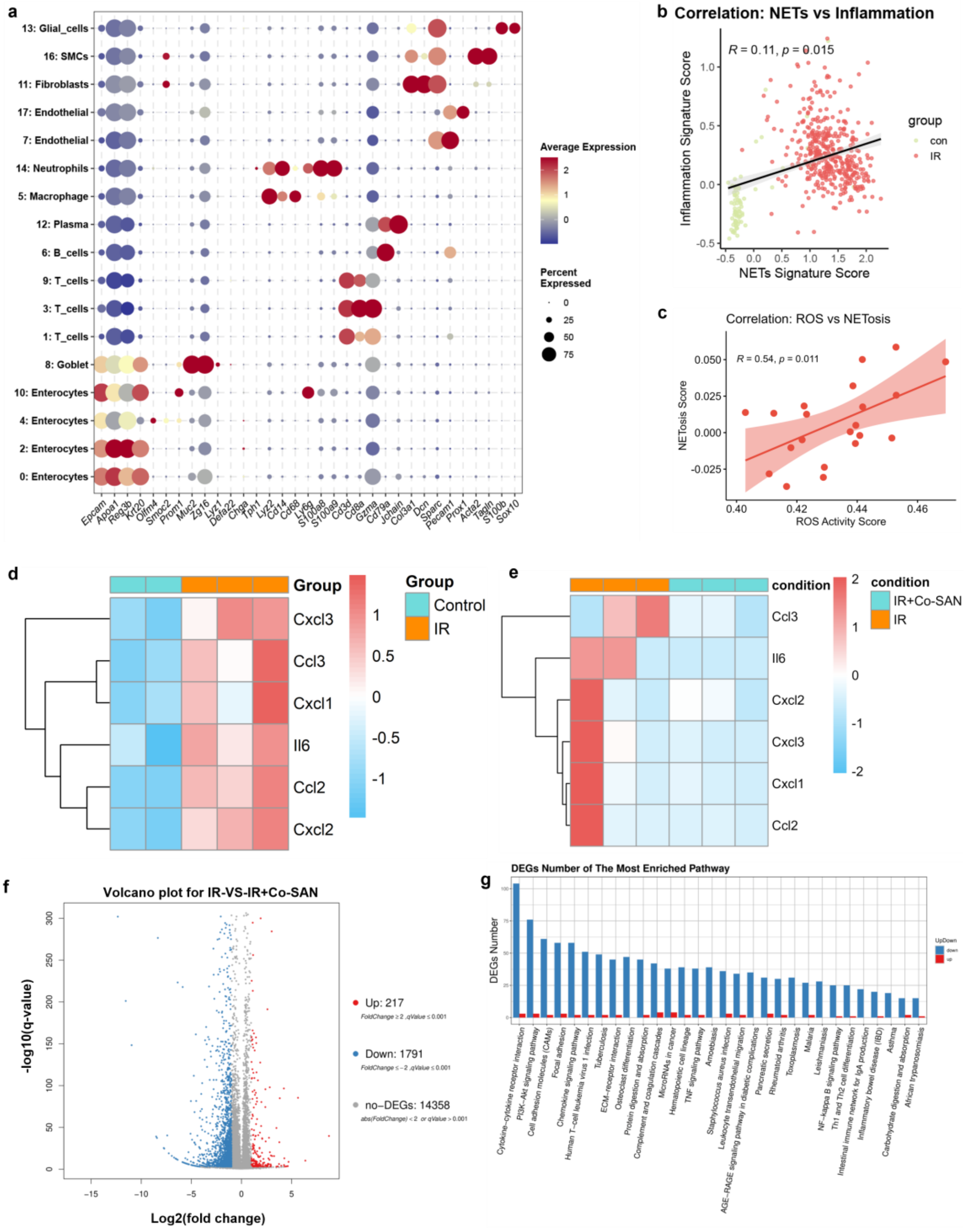

Supplementary Fig. 22. Inflammation-related pathways are downregulated in the Co-SAN group. (a) Dot plot showing the expression of selected marker genes (x-axis) across different cell types (y-axis). The size of the dot represents the percentage of cells expressing the gene within a cluster, and the color intensity indicates the average expression level. (b) Scatter plot of NETs vs. inflammation scores. The plot shows the Pearson correlation between the single-cell NETs signature score and the inflammation signature score across two groups: con (green) and IR (red). (c) Correlation Analysis between ROS Pathway Activity and NETs (Data source: GSE308064). (d) Relative abundance heatmap of the chemokine genes of the samples in different groups in genus level (data source: GSE145131). (e) Relative abundance heatmap of the chemokine genes of the samples in different groups in genus level. (f) Volcano plot for metabolite analysis in mice from PBS and Co-SAN mice after 12 Gy abdominal IR. Red dots denote the significantly upregulated metabolites, blue dots denote the significantly downregulated metabolites, and gray dots denote the insignificant metabolites. (g) mice were treated with 12 Gy abdominal X-ray (IR) and Co-SAN then subjected to RNA-seq

analysis. Pathway enriched analysis shows the top 30 enriched pathways with significant difference. Mice were treated with 12 Gy abdominal X-ray (IR) and Co-SAN then subjected to RNA-seq analysis.

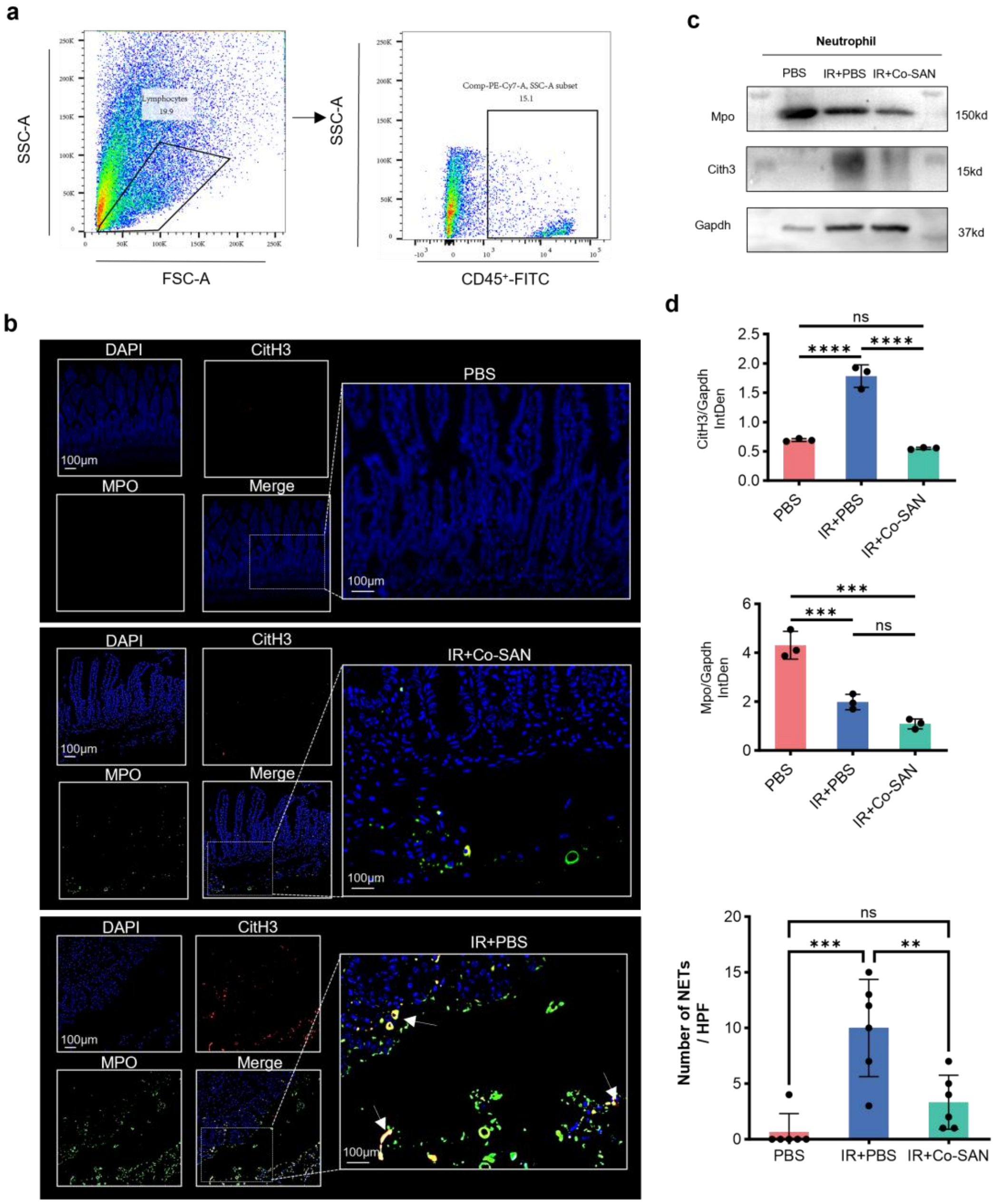

Supplementary Fig. 23. Co-SAN affect neutrophil chemotaxis and NETs formation to attenuate injury. (a) Example of a flow-through trap door. (b) Representative IF images showing the co-localization of CitH3 and Mpo in the intestine in each group after IR 5 days (n = 5 biologically independent animals). Scale bar =100 µm. The data show means ± SD. Data were analyzed by one-way ANOVA followed by Dunnett's multiple comparisons test (vs. IR group). ***p < 0.001. The Co-localization correlation between CitH3 and Mpo was analyzed (n=5 randomly selected fields, Pearson correlation coefficient). (c-d) neutrophil with 8Gy X-ray after treated with Co-SAN (80 µg/ml) 24h, then analyzed by immunoblotting.

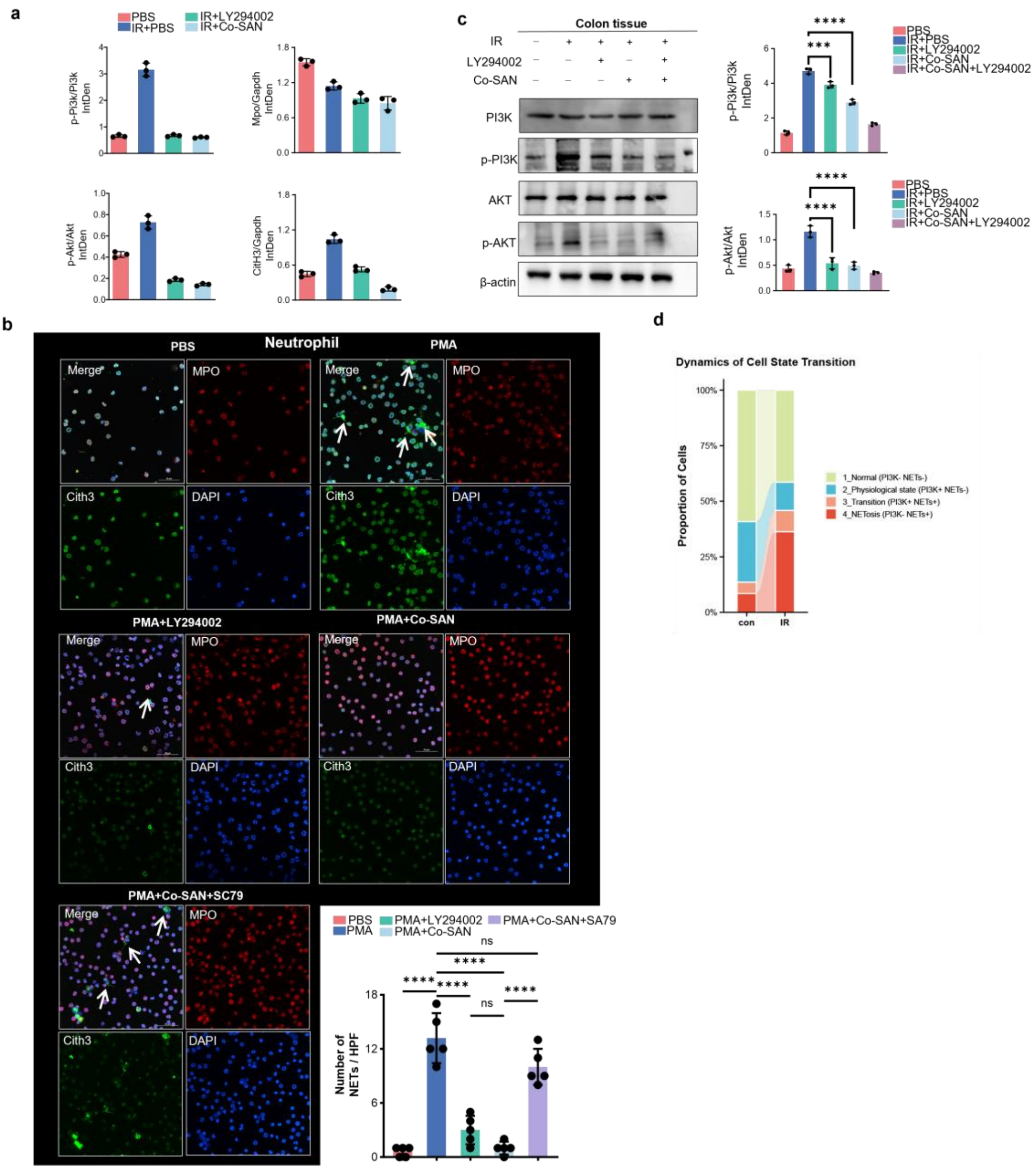

Supplementary Fig. 24. Radiation affects NETs production in neutrophils through the PI3K/AKT pathway. (a) The statistical analysis of immunoblotting. The data show means ± SD. Data were analyzed by one-way ANOVA followed by Dunnett's multiple comparisons test (vs. IR group). ***p < 0.001. (b) Representative IF images showing the co-localization of CitH3 and Mpo in the Neutrophils in each group after PMA 4 hours (n = 5). Scale bar =50 µm. The data show means ± SD. Data were analyzed by one-way ANOVA followed by Dunnett's multiple comparisons test (vs. IR group). ***p < 0.001. The co-localization correlation between CitH3 and Mpo was analyzed (n=5 randomly selected fields, Pearson correlation coefficient). (c) Intestinal tissues of each group of mice were collected for protein extraction and then analyzed by immunoblotting. (n = 3 biologically independent experiments). The data show means ± SD. Data were analyzed by one-way ANOVA followed by Dunnett's multiple comparisons test (vs. IR group). ***p < 0.001. (d) Composition and state transition of cell populations. The stacked bar chart shows the relative proportions of cells in different functional states (Normal, Physiological state, Transition, and NETosis) within the con and IR groups. Cell states were defined by the expression profiles of PI3K and NETs.

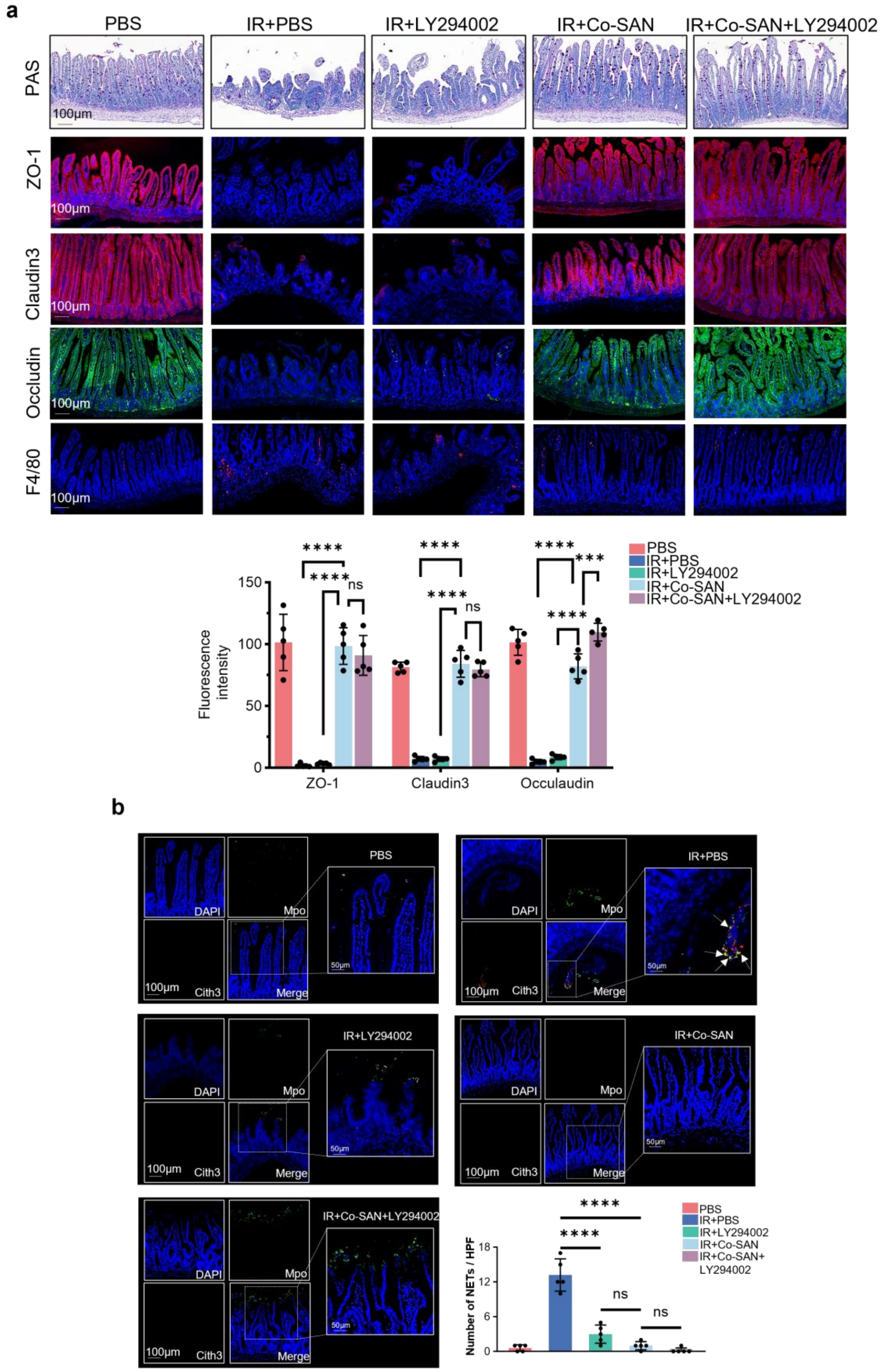

Supplementary Fig. 25. Radiation affects NETs production in neutrophils through the PI3K/AKT pathway. (a)Representative PAS and IF images showing the expression of Claudin-3, Occludin, ZO-1, F4/80 in the intestine in each group at 5 days after IR. (n = 5 biologically independent animals). The data show means ± SD. (b) Representative IF images showing the co-localization of CitH3

and Mpo in the intestine in each group at 5 days after IR. (n = 5 biologically independent animals). The data show means ± SD. Data were analyzed by one-way ANOVA followed by Dunnett's multiple comparisons test (vs. IR+Co-SAN group). ***p < 0.001.

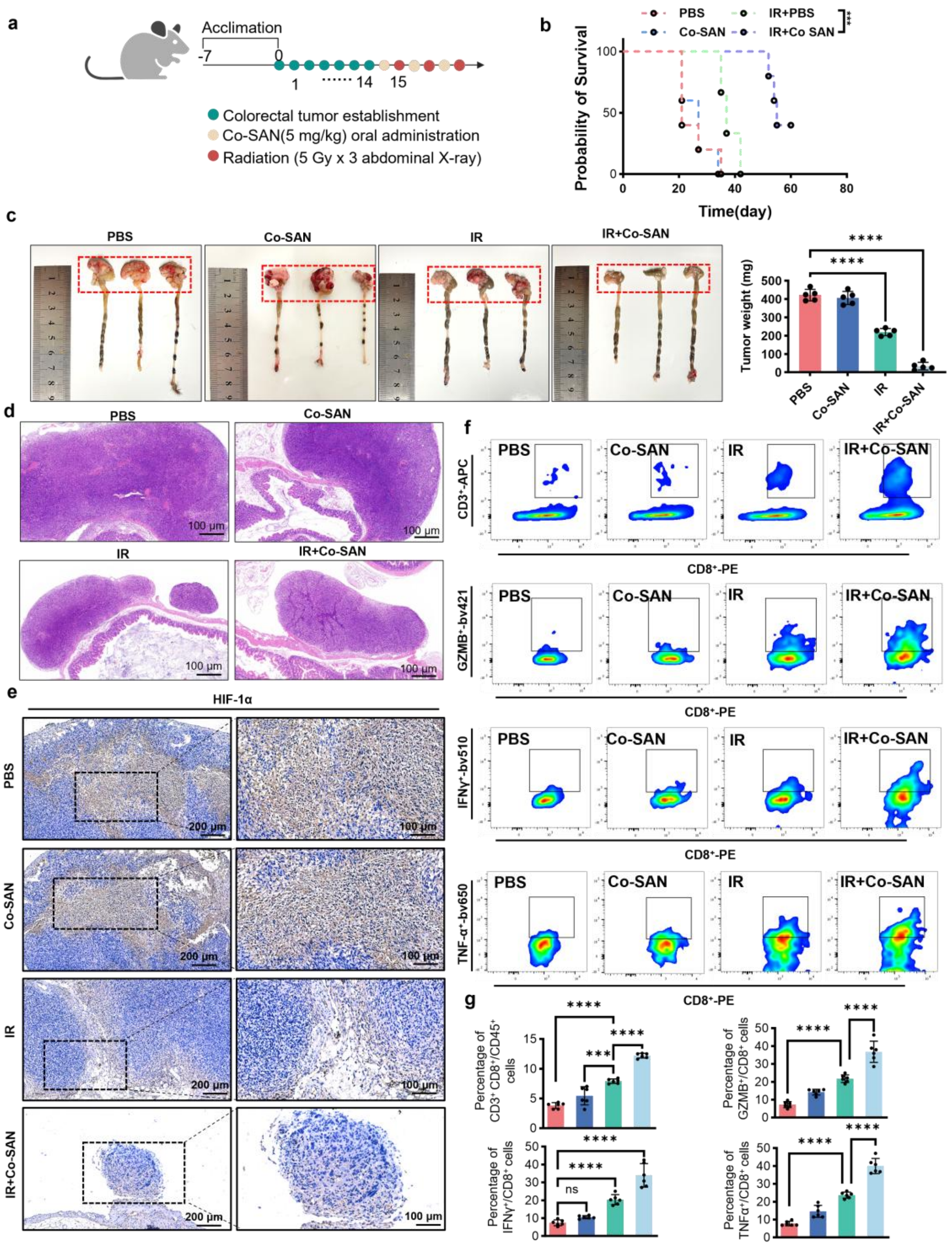

Supplementary Fig. 26. Evaluation of Co-SAN in a fractionated radiotherapy tumor model. (a) Schematic illustration of the experiment protocol. (b) Survival curves of mice after establishment of colorectal tumors (n = 5 biologically independent animals). Median survival: PBS,21 days; Co-SAN, 27 days. IR + PBS, 37 days; IR + Co-SAN, undefined (>50 days); P was calculated using Log-rank (Mantel-Cox) test (c) Tumor weight of mice in each group at 5 days post- by 5 Gy × 3 (n = 5 biologically independent animals). (d) Represented HE images. Scale bar = 100 μm. (e) Immunohistochemistry images of HIF-1α (a marker of hypoxia) in tumor tissues following treatments with PBS, Co-SAN, IR+PBS, or the combination of IR+Co-SAN. Scale bar = 100 μm. (f-g) Representative FACS plots and quantification of $CD8^+CD3^+CD45^+$ cells(f); $GZMB^+CD8^+$ cells ; $IFN\gamma^+CD8^+$ cells and $TNF\alpha^+CD8^+$

cells in the tumor of each group (mean ± SEM, n=5 independent experiments. The statistical graph is shown in the Figure. (n = 6 biologically independent animals). The data show means ± SD. Data were analyzed by one-way ANOVA followed by Dunnett's multiple comparisons test (vs. IR+Co-SAN group). ***p < 0.001.

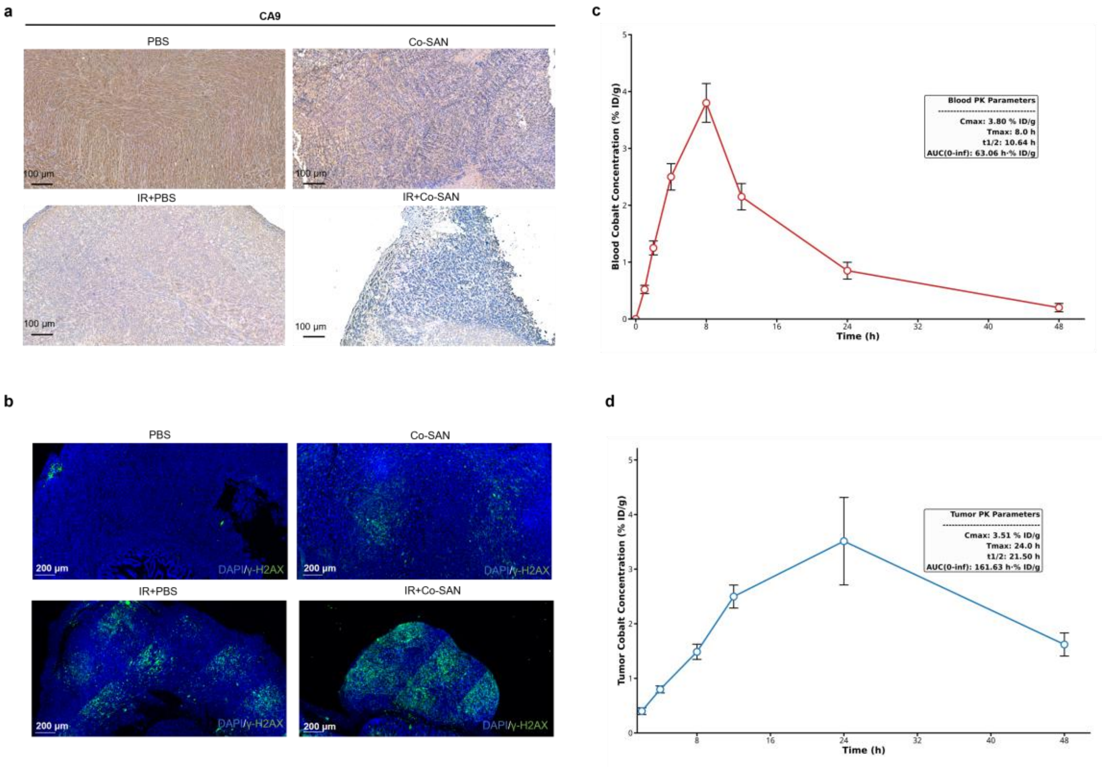

Supplementary Fig. 27 Evaluation of Co-SAN in radiotherapy tumor model. (a) immunohistochemistry images of CA9 (a marker of hypoxia) in tumor tissues following treatments with PBS, Co-SAN, IR+PBS, or the combination of IR+Co-SAN. Scale bar = 100 μm. (b) Representative IF images showing the expression of γ-H2AX in the tumor in each group. Scale bar =200 μm. (n = 5 biologically independent animals). (c) Blood concentration-time curve of Co element within 48 hours post-oral administration of Co-SAN (100 μg /mouse). The inset displays the magnified absorption phase from 0 to 12 hours. Data are presented as mean ± SD (n = 5). (d) Tumor concentration-time curve of Co element within 48 hours post-oral administration of Co-SAN (100 μg /mouse). The inset displays the magnified absorption phase from 0 to 12 hours. Data are presented as mean ± SD (n = 5). Summarized key pharmacokinetic parameters analyzed via non-compartmental analysis, including time to maximum concentration (T_{max}), maximum concentration (C_{max}), area under the curve ($AUC_{0\text{-}48h}$), and elimination half-life ($t_{1/2}$).

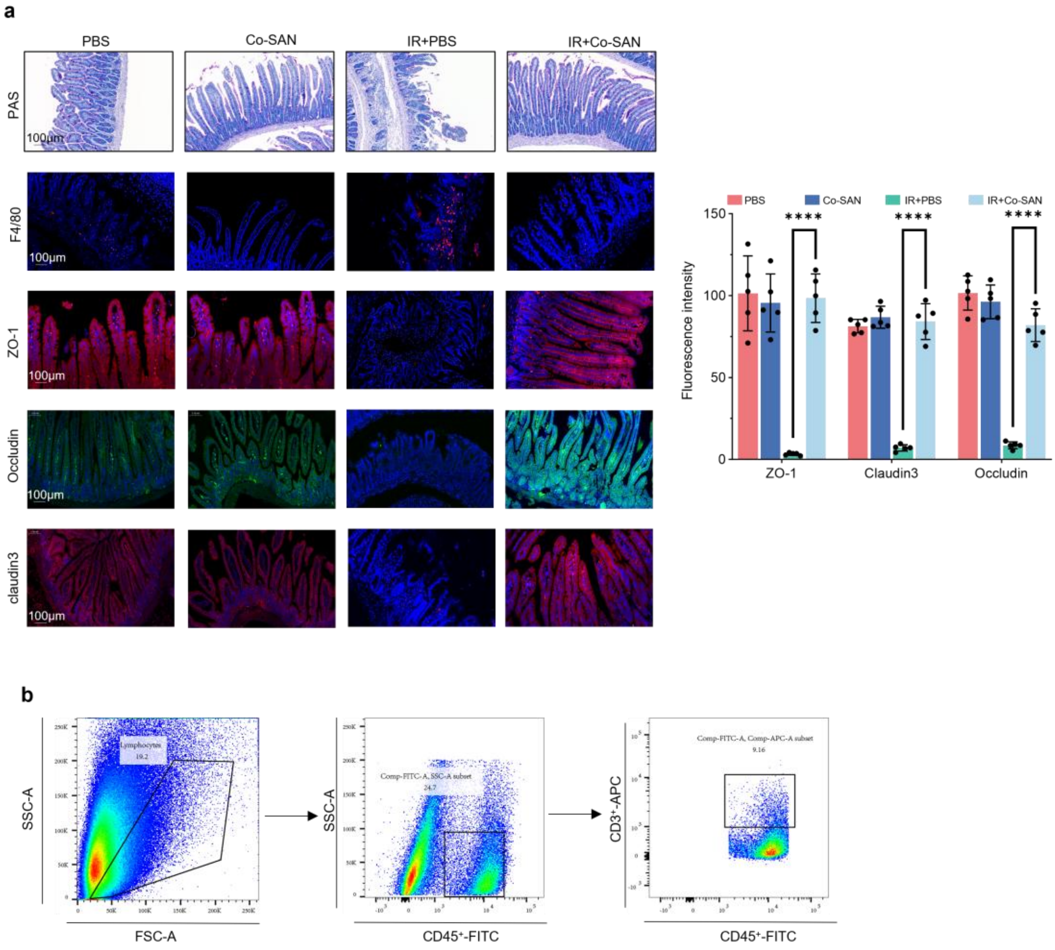

Supplementary Fig. 28 Radiotherapy Co-SAN still protects advocacy barrier function during tumor loading. (a) Representative PAS images and IF images showing the expression of Claudin-3, Occludin, ZO-1, F4/80 in the intestine in each group at 10 days after IR. (n = 5 biologically independent animals). The data show means ± SD. Scale bar = 100 μm. (b) Example diagram of a flow-through immunocyte door.

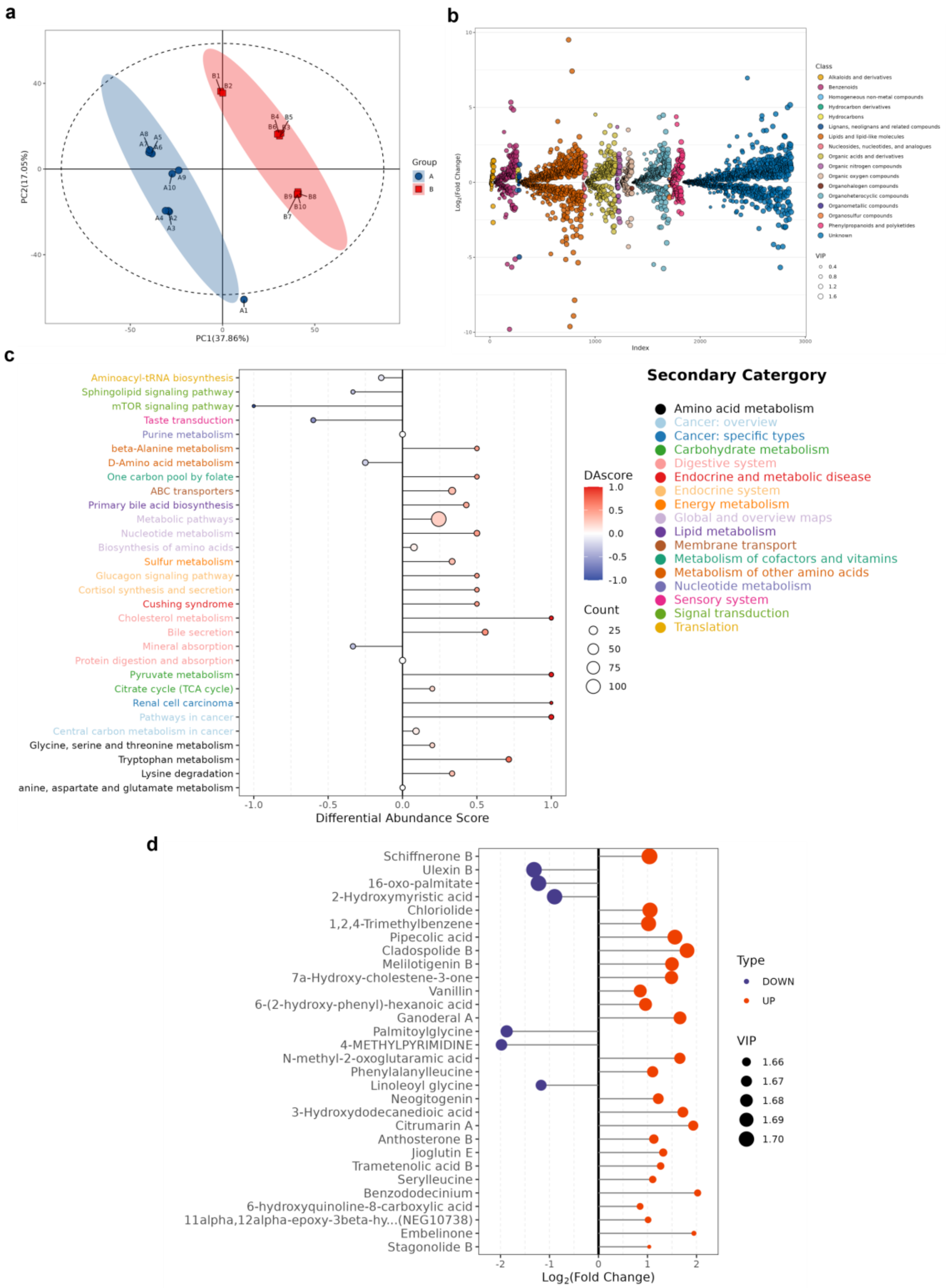

Supplementary Fig. 29. Untargeted metabolomics analysis reveals that prophylactic treatment remodels the gut metabolic profile in irradiated mice. (a) Principal Component Analysis (PCA) / Partial Least Squares Discriminant Analysis (PLS-DA) score plot of fecal samples, showing distinct metabolic separation between Group A (blue represented IR group) and Group B (red represented Co-SAN group). (b) Global distribution of all identified metabolites. The scatter plot displays the $\log_2$(Fold Change) on the y-axis, with different colors representing the specific chemical superclasses to which the metabolites belong. (c) Differential Abundance (DA) score analysis of KEGG pathways based on the altered metabolites. The x-axis represents the DA score; positive values (red

gradient) indicate an overall upregulation trend for the pathway, while negative values (blue gradient) indicate downregulation. Dot size corresponds to the number of metabolites mapped to each pathway. (d) Lollipop plot detailing the top differentially abundant metabolites (DAMs). The x-axis represents the $\log_2$(Fold Change). Dot size corresponds to the Variable Importance in Projection (VIP) score. Red dots denote significantly upregulated metabolites (Up), and blue dots denote downregulated metabolites (Down) following Co-SAN treatment.

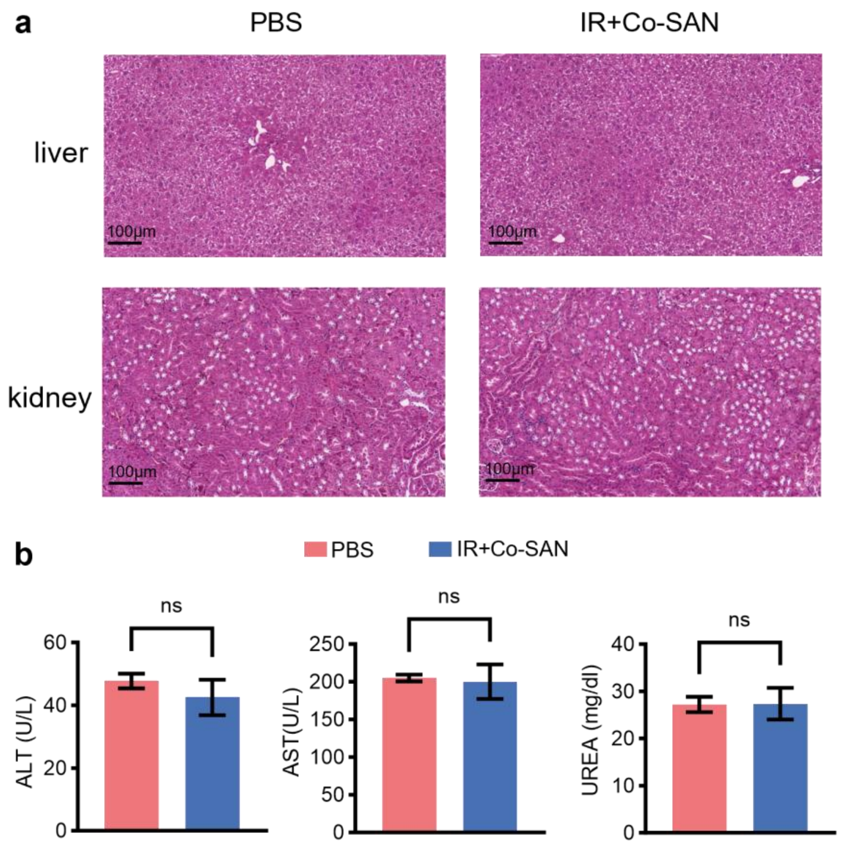

Supplementary Fig. 30 *in vivo* safety after radiation (a) Represented HE images at day 60 after treatment with PBS (Control group), IR+Co-SAN group (12Gy; 5mg/kg) (n = 3 biologically independent animals). Scale bar =100 µm. Experiment was repeated three times independently with similar results. (b) Liver and kidney function (n = 3 biologically independent animals). P between PBS group (Control) and IR+Co-SAN was calculated using two-tailed t-test. (*<0.05, **<0.01, ***<0.001, ns., no significance).

Supplementary Discussions

(On the local structure of Co sites in Co-SAN)

In our study, the atomic structure of Co-SAN was first analyzed by quantitative EXAFS fitting, which determined a Co-C/N/O coordination number of N = 4.1 at an interatomic distance of R = 1.91 Å. Regarding the inherent limitation of EXAFS in distinguishing backscatter atoms with similar atomic numbers, and therefore employed complementary XANES and theoretical studies to resolve the local

structure.

(1) Oxidation states. The XANES spectrum of Co-SAN exhibits a pre-edge peak "A1" similar to that of reference CoPc **(Supplementary Fig. 31)**., supporting the presence of quasi-planar Co-X_4 (X = C, N, O) moiety embedded within the graphene framework (The tetrahedral geometry is ruled out, as this would produce a more pronounced pre-edge feature due to hybridized d-p states). Despite the similarities in their local structure, the enhanced shoulder peak in CoPc, associated with the 1s→4pz dipole electronic transition, precludes reliable oxidation state assignment by direct comparison of absorption edges. Instead, in the original manuscript, we refer to CoO, which shows closely matched absorption edge to Co-SAN, indicating an oxidation state of approximately +2 in the sample. This finding is further supported by additional XPS analysis.

(2) Precise coordination environment. The XANES of Co-SAN exhibit discernible absorption features, "A2" and "A3", at 7727 and 7738 eV, respectively **(Supplementary Fig. 31)**. Comprehensive XANES simulations reveal that the characteristic peak "A3" predominantly originates from the second-shell Co-C scattering at an interatomic distance (R) of ~2.60 Å, indicative of Co ligation within a pyridinic-like architecture in Co-SAN [Angew. Chem. Int. Ed. 2023, 62, e202304754.]. To elucidate the precise coordination environment, different structural models, including Co_1-N_4, Co_1-N_2O_2-1, Co1-N_2O_2-2, and Co_1-O_4 moieties **(Supplementary Fig. 31)**, were constructed and optimized by DFT calculations. The calculated binding energies were −5.11, −1.66, −1.57 and 1.89 eV, respectively. These results suggest that the Co_1-O_4 moietiy is theoretically unstable, while the mixed N, O-coordination is also less favorable compared to the Co_1-N_4 configuration.

Taken together, the combined XANES and theoretical studies consistently verify that Co-SAN preferentially adopts a stable Co_1-N_4 coordination in the carbon matrix.

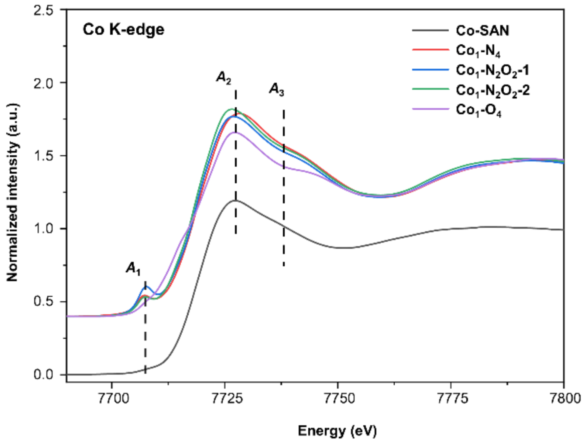

Supplementary Fig. 31. Comparison between the experimental XANES spectrum of Co-SAN (solid black line) and theoretical ones calculated with different structural models of Co_1-N_4, Co_1-N_2O_2-1, Co_1-N_2O_2-2 and Co_1-O_4. The indicated spectra are vertically offset for clarity.

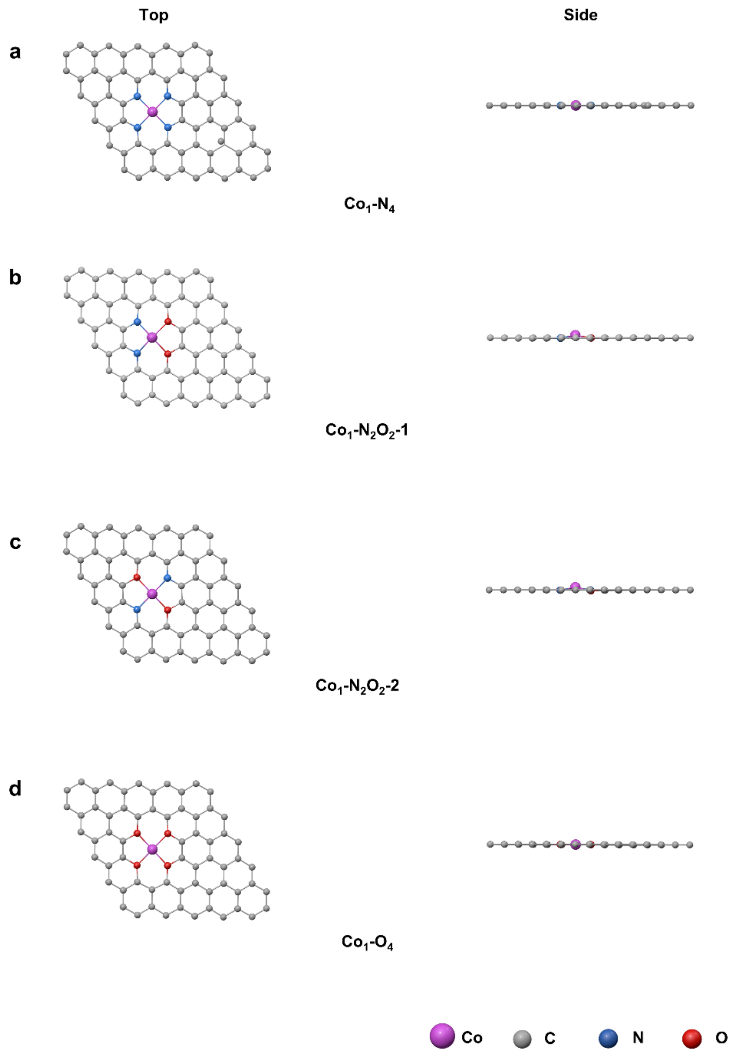

Supplementary Fig. 32. (a-d) Optimized models of Co_1-N_4, Co_1-N_2O_2-1, Co_1-N_2O_2-2 and Co_1-O_4 with different configurations (top and side view).

Supplementary Table 1. Cobalt (Co) Concentrations of Co-NPs and Co-SAN characterized by ICP-AES and EDS.

Sample	Co-NPs	Co-SAN	
Co (wt%)	29.41	2.57	ICP-AES
	28.46	2.48	EDS

Supplementary Table 2. The BET Surface Area and Pore Width of N/C, Co-NPs, ZnCo-MOF and Co-SAN.

Sample	BET Surface Area (m^2/g)	Pore Width (nm)
NC	1560.2	0.72
Co-NPs	198.1	1.45
ZnCo-MOF	1952.7	0.74
Co-SAN	1344.6	0.82

Supplementary Table 3. Fitting Parameters of Co K-edge EXAFS curve for different samples.

Sample	Path	CN[a]	R (Å)[b]	σ^2 (10^{-3}Å^2)[c]	ΔE_0(eV)[d]	R factor (%)
Co foil	Co-Co	12*	2.489±0.001	5.9	5.8	0.10
CoPc	Co-N	4.0±0.1	1.918±0.004	2.9	9.8	0.58
	Co-N-C	7.6±0.4	2.948±0.008	5.6		
Co-SAN	Co-N	4.1±0.1	1.909±0.014	2.9	-4.7	0.99

[a]CN, coordination number; [b]R, the distance between absorber and backscatter atoms; [c]σ^2, the Debye Waller factor value; [d]ΔE_0, inner potential correction to account for the difference in the inner potential between the sample and the reference compound; R factor indicates the goodness of the fit. S_0^2 was fixed to 0.879, according to the experimental EXAFS fit of Co foil by fixing CN as the known crystallographic value. * This value was fixed during EXAFS fitting, based on the known structure of Co. Fitting conditions: k range: ~2.0 - ~10.0; R range: 1.0-2.5; fitting space: R space; k-weight = 2. A reasonable range of EXAFS fitting parameters: $0.800 < S_0^2 < 1.000$; CN > 0; $\sigma^2 > 0$ Å^2; $|\Delta E_0| < 15$ eV; R factor < 0.02.

Supplementary Table 4. The retention content of Co within Co-SAN was investigated under simulated gastric fluid (SGF) environment and simulated intestinal fluid (SIF) environment.

	SGF	SIF
Time (h)	1	12
Co retention (%)	91.69	99.74

Supplementary Table 5. Comparison of CAT-mimic kinetic parameters of Co-SAN with other single-atom nanoenzymes.

Nanozymes	Substrates	K_m (mM)	V_{max} (mM s^{-1})	Ref.
Fe-SANzyme	H_2O_2	18.80	9.32×10^{-3}	1
FeN_4C	H_2O_2	55.57	9.97×10^{-4}	1
Fe-B/N-C	H_2O_2	16.82	7.33×10^{-4}	2
Co-N_4 SAzyme	H_2O_2	24.00	3.72×10^{-2}	3
Co-N_3P SAzyme	H_2O_2	31.80	3.15×10^{-2}	3
Co-SAN	H_2O_2	13.66	3.70×10^{-2}	This work

Supplementary Table 6. Comparison of OXD-mimic kinetic parameters of Co-SAN with other single-atom nanoenzymes.

Nanozymes	Substrates	K_m (mM)	V_{max} (μM s^{-1})	Ref.
Mo SAzyme	TMB	0.59	6.54×10^{-2}	4
CoN_5 SA/CNF	TMB	0.68	1.77×10^{-1}	5
Ir-N_5 SA	TMB	0.97	6.14×10^{-2}	6
Ir-N_4 SA	TMB	1.08	4.59×10^{-2}	6
Fe-N-C	TMB	0.47	6.83×10^{-2}	2
Co-SAN	TMB	0.41	3.40×10^{-2}	This work

Supplementary Table 7. Comparison of POD-mimic kinetic parameters of Co-SAN with other single-atom nanoenzymes.

Nanozymes	Substrates	K_m (mM)	V_{max} (μM s^{-1})	Ref.
Pt/C	TMB	7.74	1.85	7
	H_2O_2	41.53	4.26×10^{-1}	
FeBNC	TMB	2.22	1.81	8
	H_2O_2	25.24	1.28	
Mn_{SA}-N_4-C	TMB	1.71	4.08×10^{-1}	9
	H_2O_2	3.74×10^{3}	4.99×10^{-1}	

Mn_{SA}-N_3-C	TMB	0.54	6.33×10^{-1}	9
	H_2O_2	1.20×10^{3}	6.27×10^{-1}	
Co-SAN	TMB	0.37	6.90×10^{-2}	This work
	H_2O_2	22.31	3.70×10^{-2}	

MATERIALS AND METHODS

Synthesis of ZIF-8, ZIF-67 and ZnCo-MOF.

Regarding the synthesis of typical ZIF-8, 2-methylimidazole (1.232 g) was first dispersed in methanol solution (100 mL) and subsequently added to methanol solution (100 mL) containing $Zn(NO_3)_2$•$6H_2O$ (1.116 g). The resulting mixture was sonicated for 5 min at room temperature and then left at 37 °C overnight. The precipitate from the reaction was washed three times with methanol and dried in vacuum at 65 °C. ZIF-67 was synthesis by replacing $Zn(NO_3)_2$•$6H_2O$ (1.116 g) with $Co(NO_3)_2$•$6H_2O$ (1.0 g) using a similar synthetic method to that of ZIF-8. To synthesis ZnCo-MOF, $Zn(NO_3)_2$•$6H_2O$ (1.116 g) and $Co(NO_3)_2$•$6H_2O$ (0.5 g) were dispersed in methanol solution (100 mL), followed by sonication at room temperature and simultaneous injection of methanol solution containing 2-methylimidazole (1.232 g) (100 mL) and allowed to stand at 37 °C for 6 h. The precipitate was washed three times with methanol and dried under vacuum at 65 °C.

Synthesis of N/C, Co-NPs and Co-SAN.

The vacuum-dried ZIF-8, ZIF-67 and ZnCo-MOF powders were placed in a tube furnace and then increased to 900 °C at a temperature increase rate of 5 °C /min under the protection of a flowing N_2 atmosphere and maintained 3 h under 900 °C. The samples were cooled to room temperature to collect N/C, Co-NPs and Co-SAN.

***In vitro* toxicity and radioprotective effect.**

Small intestinal epithelium cells of Rat, IEC-6 (ATCC CRL-1592, Yuchi Bioscience, Guangzhou), were cultured in DMEM (Dulbecco's Modified Eagle Medium) with 10% FBS, 1% antibiotics (100U/mL penicillin and 100 μg/mL streptomycin) at 37 °C with 5% CO2. In order to test the toxicity of materials, IEC-6 cells were cultured in 96-well plates (2.5 x 103 per well) over night and then incubated with PBS containing different concentrations of Co-SAN (0,10 ,20 ,40 ,60, 80 μg/mL) for 24 h. The same dose of Co-NPs and N/C were added in the cells of the other two groups. Cells treated with isodose Co-NPs or N/C served as controls. Cell viability was subsequently determined utilizing a Cell Counting Kit-8 (CCK-8) assay (NCM Biotech, Suzhou, China). To assess the radiation protection capability, IEC-6 cells were seeded in 6 cm plates overnight and incubated with DMEM containing PBS, N/C (80 μg/mL), Co-NPs (80 μg/mL) and Co-SAN (80 μg/mL), respectively. After incubation for 24 h, the plates were centrifuged (500 g, 3 min) and the supernatant was softly removed. After the plates were thoroughly washed to remove excess Co-NPs, N/C, Co-SAN, then were exposed to 8 Gy of X-ray at a dose rate of 500 cGy/min (X-RAD, Vital Beam, USA). For the evaluation DNA double-strand breaks (DSB), the irradiated cells were fixed by paraformaldehyde (PFA, 4%, 30 min), treated with Triton X-100 (10 min) and goat serum (2%, 1 h), then incubated with γ-H2AX antibody (Rabbit polyclonal IgG, 1:400, Cell Signaling Technology, USA, Cat. 2577S) (4 °C, 14 h) and secondary antibody (Goat Anti-Rabbit IgG, 1:800, Thermo fisher, Guangzhou China, Cat. A11029) (25 °C, 1 h). Nuclei of the cells were stained by 4-6-diamidino-2-phenylindole (DAPI). The stained cells were visualized by Pannoramic SCAN (3DHISTECH, Hungary). To evaluate if the Co-SAN has a selective protective effect on normal

intestinal cells under various X-ray doses, both IEC-6 cells and CT26 cells (ATCC, CRL-2638, EKBioscience, Shanghai) (mouse colorectal cancer cells) were cultured and treated with different materials as mentioned. Then they were incubated with the renewed medium for 4 h before being exposed to 0, 2, 4, and 8 Gy of X-ray, respectively. The irradiated cells were seeded into six well plates and cultured for 7 days for colony formation assay. Cells were then fixed with methanol and stained with crystal violet staining solution (0.1%) for visualization of colonies. Surviving colonies with more than 50 cells were counted. The surviving fraction was calculated by normalizing the colony counts to the initial seeding density and the plating efficiency of the respective non-irradiated controls. To assess the production of reactive oxygen species (ROS) of irradiated cells, ROS assay kit (Elabscience, Wuhan, China) was used to test ROS production.

***In vivo* biodistribution.**

To observe the biodistribution of the material, a fluorescent cyanine dye, Cy7 (Selleck, 943298-08-6, USA), was loaded into Co-SAN to construct Co-SAN@Cy7. 5 mg of the as-synthesized Co-SAN was dispersed in 5 mL of PBS solution that contains 5 mg of Cy7. Then, the solution was softly shaken in a dark environment at 4 °C for 24 h. Afterward, Co-SAN@Cy7 was separated from the solution by centrifugation (3200 g, 10 min). The sediment was then collected and thoroughly washed for further usage. Five-week-old male C57BL/6J mice were fasted for 12 h, and then given Co-SAN@Cy7 PBS suspension or Cy7 PBS solution by oral gavage at the dose of 5 mg Cy7/kg. After 0, 1, 2, 4, 8 and 12 h. the mice were imaged by an in-Vivo FX Pro (Bruker, Germany) using the channel of Cy7 (excitation wavelength: 740 nm, emission wavelength: 770 nm). The Fluorescence intensity of vital organs (intestines, heart, liver, spleen, kidneys, lungs) was analyzed by Bruker MI SE (Bruker, Germany).

Protective effect against intestinal radiation injury.

Five weeks old male C57BL/6J mice were randomly divided into five groups including PBS (normal control), IR+PBS (irradiation injury group), IR+N/C, IR+Co-NPs, IR+Co-SAN. After 12 h of fasting, the mice in the IR+Co-SAN group were orally administered 5 mg/kg Co-SAN dispersed in the 100 μL of PBS. For comparison, the mice were administered with the same amount of PBS, N/C and Co-NPs group. After 4 h, the abdomen (up to diaphragm, down to pelvis) of mice in IR groups were exposed to 12 Gy of X-ray at the dose rate of 500 cGy/min (X-RAD, Vital Beam, USA). To empirically determine the optimal prophylactic window between Co-SAN administration and irradiation, mice were exposed to 12 Gy of X-ray irradiation at predetermined time intervals post-administration. On day 5 post-irradiation, intestinal tissues were harvested, and the severity of radiation-induced mucosal damage was evaluated via ImageJ-based quantification of intestinal villus parameters. The Peripheral blood of mice and Intestinal tissue from mice were homogenized and analyzed by an enzyme-linked immunosorbent assay (ELISA) kit (ELK Biotechnology CO., Wu Han, China) to quantify the represented pro-inflammatory cytokines in radiation injury, including IL-6, TNF-α and IL-1β. A subset of mice was continuously monitored for body weight changes over a 30-day period. The intestines of mice were removed on the fifth day after radiotherapy, and the degree of intestinal radiation damage was analyzed by HE and PAS staining, and immunofluorescence staining was used to analyze intestinal radiation damage-related indexes, including ZO-1 (Rb polyclonal IgG, Thermo Fisher, Guangzhou, China, Cat. 61-7300), Claudin3 (Rb polyclonal IgG, Thermo Fisher, Guangzhou, China, Cat. 34-1700), Occludin (Ms polyclonal IgG, Thermo Fisher, Guangzhou, China, Cat. 33-1500), γ-H2AX (Rabbit polyclonal IgG, Cell Signaling Technology, USA), TUNEL, CD206 (Rabbit polyclonal IgG, 91992S, Cell Signaling Technology, USA) and F4/80 (Rat monoclonal IgG, ab6640, abcam, USA). To evaluate survival benefits, separate cohorts of five-week-old mice were subjected to a lethal dose of abdominopelvic X-ray

irradiation (16 Gy, 500 cGy/min) following the identical administration protocol, and their survival times were recorded. All radiation doses were selected based on established protocols. [10]

RNA Sequencing and Transcriptomic Analysis

After the mouse oral administration Co-SAN for 4 h for radiation injury modeling, intestinal tissues from different sites were collected on the fifth day and total RNA was extracted with the RNeasy Mini Kit (Qiagen). The RNA samples were then sequenced on the Illumina Novaseq platform. The sequencing quality of fastq files was assessed using FastQC (v 0.11.9) to ensure that all samples exhibited high quality. Illumina adapter sequences were trimmed using Trim galore (v 0.6.7). 150 bp paired-end reads were aligned to mouse reference genome (GRCm39) using hisat2 (v2.2.1) method.[11] Comparative analysis of differentially expressed genes based on reads was performed using DEseq2 (v1.40.2) [12] GSEA and KEGG pathway enrichment analysis were performed using the "clusterProfiler" R package (v 4.8.3) [13]and the data were visualized by the ggplot2 package (v3.4.4).

Tissue Dissociation and Flow Cytometry

For the quantification of ROS and neutrophils, Intestinal tissue was retrieved from the EP tube and placed on crushed ice. Subsequently, an appropriate volume of RPMI-1640 medium was added to wash the tissue twice, followed by weighing of the cleaned tissue. Prepared the pre-digestion solution by mixing 5ml of 1×HBSS, 50μl of 0.5M EDTA, and 1mM DTT for later use. Cut the weighed intestinal tissue into small pieces of 5mm, transfer these tissue pieces into a 50ml centrifuge tube, add the prepared pre-digestion solution, and then place the centrifuge tube in a water bath for digestion at 37°C and 145 rpm for 30 minutes. After the pre-digestion, filter the mixture with a 70μm filter sieve, wash the filtered tissue with RPMI-1640 medium, and then continue to cut the washed tissue into smaller pieces. Add DNase and collagenase to the re-cut tissue pieces, mix them well, and then perform digestion again at 37°C for 1 hour. After the secondary digestion, filter the mixture again with a 70μm filter sieve, wash the filtered tissue with RPMI-1640 medium, and then centrifuge the filtered liquid at 400g for 10 minutes. Discard the supernatant after centrifugation, retain the precipitate, and repeat the centrifugation step (centrifuging the liquid at 400g for 10 minutes, discarding the supernatant and retaining the precipitate). After washing twice, the supernatant was discarded and the pellet was retained for flow cytometry analysis of ROS and neutrophils. An aliquot of the pellet was taken and incubated with antibodies CD45(FITC, 147710, Biolegend, China), CD11b (PE ,101207, Biolegend, China), Ly6g (APC, 164506, Biolegend, China) incubation at 4°C, protected from light, for 30min, next was washed and resuspended in HBSS for flow cytometry acquisition. Tissue sections from different groups were selected for immunofluorescence staining for NETs indicators (Mpo and CitH3). The sections were scanned and analyzed by Pannoramic SCAN (3DHISTECH, Hungary).

In vivo Pharmacological Inhibition and Immunoblotting

To elucidate the in vivo role of the PI3K/AKT axis, Five-week-old male C57BL/6J were randomly divided into five groups including PBS (PBS group), IR+PBS (irradiation injury group), IR+LY294002, IR+Co-SAN, IR+Co-SAN+LY294002. After 12 h of fasting, the mice in the IR+ Co-SAN+LY294002 group were orally administered 5 mg/kg Co-SAN dispersed in the 100 μL PBS, then were exposed to 12 Gy of X-ray at the dose rate of 500 cGy/min (X-RAD, Vital Beam, USA), and LY294002 (MCE, HY-10108, USA, 10 mg/kg) was administered intraperitoneally for 5 consecutive days after radiotherapy to inhibit PI3K/AKT pathway. A subset of mice was monitored for body weight variations over 30 days. The intestines of mice were removed on the fifth day after radiotherapy, and the degree of intestinal radiation damage was analyzed by HE and PAS staining, and immunofluorescence staining was used to analyze intestinal radiation damage-related indexes, including ZO-1, claudin3, Occludin, γ-H2AX,

TUNEL, CD206 and F4/80. The inhibitory effects of inhibitor LY294002 and Co-SAN on the PI3K/Akt pathway, PI3K (Rabbit polyclonal IgG, ab191606, abcam, USA), p-PI3K (Rabbit polyclonal IgG,4228S, Cell Signaling Technology, USA), AKT (Rabbit polyclonal IgG, 9272S, Cell Signaling Technology, USA), p-AKT (Rabbit polyclonal IgG, 9271S, Cell Signaling Technology, USA) *in vitro* and *in vivo* were verified by immunoblotting analysis, Tissues and cells were first lysed by RIPA lysate on ice for 30 min, followed by 12,000 g, centrifugation for 10 min, and collection of supernatant, and BCA determination of protein concentration for immunoblotting analysis.

scRNA-seq Data Processing and Quality Control

Raw sequencing data were processed using the Cell Ranger pipeline (version 2.2.0). With mm10 as the reference genome, sequence alignment, barcode demultiplexing, and UMI counting were performed to construct a gene-cell expression matrix. Subsequent dimensionality reduction, clustering, and downstream analyses were conducted using the Seurat package (v4.3.2) in R. To obtain high-quality single-cell transcriptomic data, strict quality control was implemented for all samples. After quality control filtering, the expression matrix was log-normalized using the NormalizeData function. The FindVariableFeatures function with the vst method was applied to identify 2000 highly variable genes (HVGs). All gene expression levels were further scaled and centered via the ScaleData function.

Dimensionality Reduction, Unsupervised Clustering, and Cell Type Annotation

Based on the identified HVGs, principal component analysis (PCA) was used for linear dimensionality reduction. According to the ElbowPlot analysis, the top 20 principal components (PCs) were selected to construct a k-nearest neighbor (KNN) graph. The Louvain algorithm was adopted for unsupervised cell clustering with the resolution set to 0.3. The uniform manifold approximation and projection (UMAP) algorithm was used for two-dimensional cell visualization.

To identify cluster-specific differentially expressed genes (DEGs), non-parametric Wilcoxon rank-sum tests were employed. The statistical thresholds were stringently set to a minimum $\log_2$(fold change) of 0.25 and an expression frequency of > 0.1 within the corresponding cluster. Combined with canonical cell marker genes, all cell subsets were annotated into 11 major cell types, including enterocytes, goblet cells, fibroblasts, smooth muscle cells (SMCs), endothelial cells, glial cells, and immune cell populations such as T cells, B cells, plasma cells, macrophages, and neutrophils.

Cell Proportion and Differential Gene Expression Analysis

To evaluate the differences in cellular composition within the tissue microenvironment between the control group (con) and the 12 Gy irradiation group (IR), the number of each cell type in each sample was counted, and the proportions of key cell types including B cells, macrophages, neutrophils, and T cells were calculated. The FindMarkers function in the Seurat package (Wilcoxon rank-sum test) was used to compare gene expression profiles of the same cell subpopulations across different experimental groups, so as to screen DEGs significantly upregulated or downregulated after 12 Gy irradiation.

Functional Enrichment Analysis

To elucidate the alterations in biological processes induced by 12 Gy irradiation, significantly upregulated DEGs from targeted cell subpopulations were extracted. Gene Ontology Biological Process (GO-BP) enrichment analysis was performed using the R package clusterProfiler (v4.10.0). A Statistical significance was strictly defined by a Benjamini-Hochberg adjusted P value < 0.05 . Key immune- and inflammation-associated GO terms—notably neutrophil activation and the positive regulation of macrophage cytokine production—were subsequently visualized using dot plots.

Biological Potential Evaluation and Gene Set Scoring

To quantitatively evaluate the functional states of individual cells in the control and irradiated (12 Gy) cohorts, specific transcriptional signatures were curated to represent NETosis and inflammation potentials. Single-cell module enrichment scores for these defined gene sets were calculated utilizing a background-corrected scoring algorithm implemented in the Seurat package. Briefly, this method computes the average expression levels of the target signature per cell and normalizes them against randomly selected background gene bins with comparable baseline expression profiles. This rigorous approach effectively mitigates technical biases, thereby robustly quantifying functional phenotypic shifts at single-cell resolution.

Establishment of the Orthotopic Colorectal Tumor Model.

To establish the orthotopic tumor model, murine MC38 colon adenocarcinoma cells (Fuheng Biology, Shanghai, China) were cultured to the exponential growth phase and resuspended in sterile PBS at a density of 10^7 cells in 1 mL. Following the administration of adequate anesthesia and strict abdominal antisepsis to 5-week-old male C57BL/6J mice, a 0.5-cm midline laparotomy was performed. The cecum was gently exteriorized, and 50 μL of the cell suspension was carefully injected into the cecal serosa. Gentle pressure was applied to the injection site to prevent cell leakage, after which the cecum was relocated into the abdominal cavity, and the abdominal wall was sutured in anatomical layers.

Treatment Protocols and Endpoint Evaluations

Post-engraftment, the tumor-bearing mice were randomized into four cohorts: PBS, Co-SAN, IR + PBS, and IR + Co-SAN. The materials were administered via the previously delineated prophylactic protocols, followed by a localized 16 Gy abdominopelvic X-ray irradiation. To thoroughly evaluate the therapeutic outcomes, separate parallel cohorts were established. For the assessment of intestinal injury, small intestines were harvested on day 5 post-treatment and sectioned for H&E and immunofluorescence staining. To evaluate tumor suppression, orthotopic large intestinal tumors were excised and weighed on day 25. Furthermore, independent cohorts of treated mice were monitored over a 60-day observation period to record overall survival.

Tumor Dissociation and Flow Cytometry

To elucidate the tumor immune microenvironment, orthotopic tumors were harvested on day 10 post-treatment. Excised tumor tissues were washed in RPMI-1640 medium, mechanically minced, and enzymatically digested in a buffer containing collagenase and DNase for 1 h at 37 °C. The resulting suspension was passed through a 70-μm cell strainer, and the cells were isolated via centrifugation (400 × g, 10min, repeated twice). To inhibit protein transport and accumulate intracellular cytokines, the cell pellets were resuspended in complete RPMI-1640 medium (supplemented with 10% FBS) containing 3 μL/mL Brefeldin A (00-4506, Thermo Fisher) and 2 μL/mL Monensin (00-4505, Thermo Fisher, China), followed by incubation for 4 hours. Following incubation, the cells were centrifuged (300 × g, 5 minutes) and washed. For surface marker profiling, the cells were incubated with CD45(APC/Cyanine7, Biolegend, China, Cat. 103116), CD3(FITC, Biolegend, China, Cat. 100204), CD8a (PerCP/cyanine5.5, Biolegend, China, Cat. 100734) incubation at 4°C, protected from light, for 30min. Next, using the Fixation/Permeabilization Buffer Set (Biolegend), the supernatant was discarded according to the instructions, and the cell precipitate was retained for incubation with antibodies GZMB (Brilliant Violet 421TM, Biolegend, China, Cat. 396414), IFN-γ (Brilliant Violet 510TM, Biolegend, China, Cat. 505842,), TNF-α (Brilliant Violet 650TM, Biolegend, China, Cat. 506333) incubation at 4°C and incubation with DCFH-DA at 25°C, protected from light, for 30min, and was washed and resuspended in HBSS prior to flow cytometric acquisition.

Effect of Fractionated Radiotherapy on Orthotopic Colorectal Cancer

Post-engraftment, the mice were randomized into four cohorts: PBS, Co-SAN, IR + PBS, and IR + Co-SAN. Treatments were administered following the previously delineated prophylactic protocols. For the irradiated cohorts, a fractionated abdominopelvic X-ray radiotherapy regimen was applied (5 Gy per fraction, administered every other day for a total of three fractions). To thoroughly evaluate therapeutic outcomes across different endpoints, parallel cohorts were established: on day 25 post-treatment, orthotopic large intestinal tumors were excised and weighed to assess tumor suppression; independently, tumor-bearing cohorts were monitored over a 60-day observation period to record overall survival.

Histological and Immunohistochemical Analyses

Isolated tumor and intestinal tissue samples were fixed in 4% paraformaldehyde (PFA) for 24 h, followed by routine dehydration and paraffin embedding. The tissues were sectioned at a thickness of 4–5 μm. After deparaffinization and rehydration, the sections were subjected to standard HE staining to evaluate tumor necrotic/apoptotic regions (tumor regression) and the microstructural integrity of intestinal tissues. To assess the alleviative effect of Co SAN on tumor microenvironmental hypoxia and its enhancement of radiosensitivity, paraffin embedded tumor sections were processed for immunofluorescence (IF) or immunohistochemistry (IHC) staining. Following deparaffinization, antigen retrieval using sodium citrate buffer (pH 6.0), and blocking, the sections were incubated overnight at 4 °C with primary antibodies against hypoxia markers, including anti HIF-1α (Rabbit polyclonal IgG, 1:400, Cat. 82989-4-RR, Proteintech, China) and anti CA9 (Rabbit polyclonal IgG, 1:400, Cat. 11071-1-AP, Proteintech, China). anti γ-H2AX (Rabbit polyclonal IgG, 1:400, Cat. 2577S, Cell Signaling Technology, USA), was used to evaluate the extent of DNA damage, and Alexa Fluor 488-conjugated anti-rabbit IgG (1:800, Cat. ab150077, abcam, USA) (25 °C, 1 h) or HRP conjugated secondary antibodies (for IHC) at room temperature for 1 h. IF sections were counterstained with DAPI for nuclear staining. IHC sections were developed with DAB chromogen.

Long-term safety profiles.

To assess the long-term safety of Co-SAN, 5-week-old male C57BL/6J mice were administered consecutive daily doses of Co-SAN (5 mg/kg) for 60 days. The mice in other groups were given the same amount of PBS as the Co-SAN group. The body weight of mice was recorded. The survival time was monitored. Following the 60-day administration period, the mice were euthanized, collecting the blood and major organs for the hematological and pathological examinations.

Isolation of Bone Marrow-Derived Neutrophils (BMDNs)

Healthy 5-6-week-old male C57BL/6J mice were euthanized, and their intact femurs and tibias were surgically excised under aseptic conditions. The isolated bones were sequentially immersed in sterile 75% ethanol for 2 min (repeated three times) and subsequently washed three times in sterile PBS supplemented with 1% penicillin-streptomycin. The epiphyses were carefully removed to preserve the marrow cavity, and the bone marrow was gently flushed out using an insulin syringe filled with cold sterile PBS. The resulting marrow suspension was filtered through a cell strainer and collected in a 50-mL centrifuge tube. Following centrifugation (300 × g, 5 min), the cell pellet was treated with 1-2 mL of red blood cell (RBC) lysis buffer for 2-3 min. Lysis was rapidly halted by adding a threefold volume of complete RPMI-1640 medium. The cells were then centrifuged again (300 × g, 5 min), resuspended in sorting buffer, and accurately counted to ensure optimal downstream magnetic labeling.

Magnetic-Activated Cell Sorting (MACS)

BMDNs were highly purified via positive selection utilizing an anti-Ly6G MicroBeads Kit (130-120-337, Miltenyi, Germany) Briefly, the cells were resuspended in sorting buffer (80 μL per 10^7 cells) and incubated with 20 μL of Ly6G MicroBeads for 10 min at 4 °C in the dark. Post-incubation, the cells were

washed with 1-2 mL of buffer, centrifuged (300 × g, 10 min), and resuspended in a minimum of 1 mL of buffer. An LS column was placed in the magnetic field of a MACS separator and pre-conditioned with 3 mL of sorting buffer. The labeled cell suspension was then applied to the column in 1 mL aliquots. To maximize cell yield, the flow-through was re-loaded onto the column twice. The column was subsequently washed with 3 mL of sorting buffer to effectively deplete unlabeled cells. Finally, the column was removed from the magnetic separator, and the magnetically trapped Ly6G$^+$ neutrophils were immediately flushed out utilizing 5 mL of buffer and the supplied plunger. The purified neutrophils were pelleted via centrifugation (300 × g, 10 min) for subsequent assays.

In Vitro NETosis Assay and Immunofluorescence

To evaluate NET formation, Neutrophils were pretreated with the PI3K inhibitor LY294002 (30μM) or Co-SAN (80 μg/ml), followed by incubation with phorbol 12‑myristate 13‑acetate (PMA; MCE, Cas. 16516-29-8) at a final concentration of 100 nM for 4 h to induce neutrophil NETosis and NETs release. Cells in the control group were treated with an equal volume of PBS vehicle only. After 4 h of stimulation, the culture medium was discarded, and cells were fixed with 4% PFA at room temperature for 15 min. Following three washes with PBS, cells were permeabilized with 0.1% Triton X-100 (prepared in PBS) for 10 min. Thereafter, non-specific binding sites were blocked with PBS containing 5% bovine serum albumin (BSA) at room temperature for 1 h. After blocking, cells were incubated overnight at 4 °C with primary antibodies, including anti-myeloperoxidase (Mouse polyclonal IgG, 66177-1-Ig, Proteintech, China, 1:400) and anti-citrullinated histone H3 (Rabbit polyclonal IgG, ab5103, abcam, USA,1:400]). On the next day, after washing, cells were incubated with corresponding fluorophore-conjugated secondary antibodies [Alexa Fluor 488-conjugated anti-rabbit IgG (ab150077, abcam, USA,1:800) and Alexa Fluor 594-conjugated anti-mouse IgG (ab150116, abcam, USA,1:800)] for 1 h at room temperature in the dark. Finally, slides were mounted with DAPI-containing antifade mounting medium for nuclear and extracellular DNA network counterstaining. NETs were defined as extracellular reticular DNA structures positive for co-localized MPO (red), CitH3 (green), and DAPI (blue) staining.

Supplementary References

[1] Zhang, R. et al. Edge-Site Engineering of Defective Fe–N4 Nanozymes with Boosted Catalase-Like Performance for Retinal Vasculopathies. Adv. Mater. 34, 2205324 (2022).

[2] Liu, W. et al. Spatially Axial Boron Coordinated Single-Atom Nanozymes with Boosted Multi-Enzymatic Performances for Periodontitis Treatment. Adv. Funct. Mater. 34, 2403386 (2024).

[3] Chen, Y. et al. Atomic-Level Regulation of Cobalt Single-Atom Nanozymes: Engineering High-Efficiency Catalase Mimics. Angew. Chem. Int. Ed. 135, e202301879 (2023).

[4] Li, Z. et al. Multi-Enzyme Mimetic MoCu Dual-Atom Nanozyme Triggering Oxidative Stress Cascade Amplification for High-Efficiency Synergistic Cancer Therapy. Angew. Chem. Int. Ed. 64, e202413661 (2025).

[5] Huang, L., Chen, J., Gan, L., Wang, J. & Dong, S. Single-atom nanozymes. Sci. Adv. 5, eaav5490 (2019).

[6] Liu, Y. et al. Single-Atom Nanozyme with Asymmetric Electron Distribution for Tumor Catalytic Therapy by Disrupting Tumor Redox and Energy Metabolism Homeostasis. Adv. Mater. 35, 2208512 (2023).

[7] Wu, Y. et al. Cascade Reaction System Integrating Single-Atom Nanozymes with Abundant Cu Sites for Enhanced Biosensing. Anal. Chem. 92, 3373-3379 (2020).

[8] Jiao, L. et al. Boron-doped Fe-N-C single-atom nanozymes specifically boost peroxidase-like activity. Nano Today 35, 100971 (2020).

[9] Wang, Y. et al. Tuning Local Coordination Environments of Manganese Single-Atom Nanozymes with Multi-Enzyme Properties for Selective Colorimetric Biosensing. Angew. Chem. Int. Ed 135, e202300119 (2023).

[10] Zhang, D., et al. Microalgae-based oral microcarriers for gut microbiota homeostasis and intestinal protection in cancer radiotherapy. Nat. Commun. 13, 1413 (2022).

[11] Kim, D., Paggi, J.M., Park, C., Bennett, C. & Salzberg, S.L. Graph-based genome alignment and genotyping with HISAT2 and HISAT-genotype. Nat. Biotechnol. 37, 907-915 (2019).

[12] Love, M.I., Huber, W. & Anders, S. Moderated estimation of fold change and dispersion for RNA-seq data with DESeq2. Genome. Biol. 15, 550 (2014).

[13] Wu, T., et al. clusterProfiler 4.0: A universal enrichment tool for interpreting omics data. The Innovation 2, 100141 (2021).
